# Supplementary material for: Pastoralism in the highest peaks: Role of the traditional grazing systems in maintaining biodiversity and ecosystem function in the alpine Himalaya
Source: PLoS One. 2021 Jan 7;16(1):e0245221. doi: 10.1371/journal.pone.0245221 (PMC7790420; doi:10.1371/journal.pone.0245221)
Supplement: S1 Table — Unidentified taxa were those that we could not identify at a species, genus, or family. From this preliminary list, a refined list was prepared where synonyms were combined under a single epithet, based on the taxonomic literature. Where genus or species was not identified we used a number. For the purpose of the analysis, name of the species was not imperative but making sure they were distinguishable species. (DOCX) [file pone.0245221.s001.docx]

**S1 Table: List of species with details on their presence (+) or absence (-) in grazed and ungrazed plots sampled .** Unidentified taxa were those that we could not identify at a species, genus, or family. From this preliminary list, a refined list was prepared where synonyms were combined under a single epithet, based on the taxonomic literature. Where genus or species was not identified we used a number. For the purpose of the analysis, name of the species was not imperative but making sure they were distinguishable species.

| **Family** | **Species** | **Grazed Plots** | **Ungrazed Plots** |
| --- | --- | --- | --- |
| Amaryllidaceae | *Allium sikkimensis* | - | + |
| Apiaceae | *Chamaesium novem-jugum* | + | - |
| Apiaceae | *Selinum tenuifolium* | + | - |
| Apiaceae | *Silene caespitella* | + | + |
| Apiaceae | *sp. 1 (Apiaceae)* | - | + |
| Apiaceae | *sp. 2(Apiaceae)* | + | - |
| Apiaceae | *sp. 3 (Apiaceae)* | + | + |
| Asteraceae | *Anaphalis sp. 1* | + | - |
| Asteraceae | *Anaphelis sp. 2* | + | - |
| Asteraceae | *Artemisia indica* | + | - |
| Asteraceae | *Aster sp. 1* | + | + |
| Asteraceae | *Aster sp. 2* | + | - |
| Asteraceae | *Aster sp. 3* | - | + |
| Asteraceae | *Cremanthodium sp.* | + | - |
| Asteraceae | *Galinsoga parviflora* | - | + |
| Asteraceae | *Leontopodium haastioides* | + | - |
| Asteraceae | *Leontopodium himalayanum* | - | + |
| Asteraceae | *Saussurea heiracioides* | + | - |
| Asteraceae | *Saussurea katochaete* | + | - |
| Asteraceae | *Saussuria obvallata* | + | - |
| Asteraceae | *Soroseris glomerata* | + | - |
| Asteraceae | *sp. 1 (Asteraceae)* | + | - |
| Asteraceae | *sp. 2 (Asteraceae)* | - | + |
| Asteraceae | *sp. 3 (Asteraceae)* | + | - |
| Asteraceae | *Taraxacum sp* | + | - |
| Balsaminaceae | *Impatiens falcifer* | + | - |
| Balsaminaceae | *Impatiens glandulifera* | + | - |
| Berberidaceae | *Berberis angulosa* | - | + |
| Boraginaceae | *Cynoglossum glochidiatum* | + | - |
| Boraginaceae | *Onosma hookeri* | + | - |
| Campanulaceae | *Cyananthus incanus* | + | - |
| Celastraceae | *Parnassia nubicola* | - | + |
| Cyperaceae | *Carex alpina* | + | - |
| Cyperaceae | *Carex sp. 1* | + | + |
| Cyperaceae | *Carex sp. 2* | + | - |
| Cyperaceae | *Carex sp. 3* | - | + |
| Cyperaceae | *Kobresia nepalensis* | + | + |
| Cyperaceae | *Kobresia pygmaea* | + | + |
| Cyperaceae | *Kobresia sp. 1* | + | - |
| Ericaceae | *Cassiope fastigiata* | - | + |
| Ericaceae | *Corydalis sp* | - | + |
| Ericaceae | *Gaultheria trichophylla* | - | + |
| Euphorbiaceae | *Euphrasia sp.* | + | - |
| Fabaceae | *Gueldenstaedita himalaica* | + | - |
| Fabaceae | *Hedysarum sikkimense* | + | - |
| Fabaceae | *Oxytropis tartarica* | + | - |
| Fabaceae | *Oxytropis williamsii* | + | - |
| Fabaceae | *sp. 1 (Fabaceae)* | + | - |
| Fabaceae | *Stracheya tibetica* | - | + |
| Fabaceae | *Thermopsis barbata* | + | - |
| Gentianaceae | *Gentiana chirata* | + | - |
| Geraniaceae | *Geranium sp. 1* | - | + |
| Geraniaceae | *sp 1(Geraniaceae)* | + | - |
| Juncaceae | *Juncus sp. 1* | + | + |
| Juncaceae | *Juncus sp. 2* | + | - |
| Lamiaceae | *Mentha sp.* | + | - |
| Lamiaceae | *Nepeta nepalensis* | - | + |
| Lamiaceae | *Phlomis rotata* | + | + |
| Liliaceae | *sp. 1 (Liliaceae)* | + | - |
| Mazaceae | *Lancea tibetica* | + | - |
| Morinaceae | *Morina longifolia* | + | + |
| Orchidaceae | *Dactylorhiza hatagirea* | + | - |
| Orchidaceae | *Habernaria aitchisonii* | + | - |
| Orchidaceae | *Neottianthe calcicola* | + | - |
| Orchidaceae | *Ponerorchis chusua* | + | - |
| Orchidaceae | *Satyrium nepalense* | + | - |
| Orchidaceae | *sp. 2 (Orchidaceae)* | + | - |
| Orchidaceae | *sp1 (Orchidaceae)* | + | - |
| Orchidaceae | *Spiranthes sinensis* | + | - |
| Orobanchaceae | *Pedicularis elwesii* | + | - |
| Orobanchaceae | *Pedicularis gracilis* | + | - |
| Orobanchaceae | *Pedicularis integrifolia* | - | + |
| Orobanchaceae | *Pedicularis longiflora var. tubiformis* | + | - |
| Orobanchaceae | *Pedicularis siphonantha* | + | - |
| Oxalidaceae | *sp. 1 (Oxalidaceae)* | + | - |
| Papaveraceae | *sp. 1 (Papaveraceae)* | + | - |
| Plantaginaceae | *Digitalis sp.* | - | + |
| Plantaginaceae | *Picrorhiza kurroa* | + | - |
| Plantaginaceae | *Plantago sp. 1* | + | - |
| Plantaginaceae | *sp. 1 (Plantaginaceae)* | + | - |
| Plantaginaceae | *sp. 12(Plantaginaceae)* | + | - |
| Poaceae | *Agrostis micrantha* | - | + |
| Poaceae | *Anthraxron microphyllus* | + | + |
| Poaceae | *Festuca coelestis* | + | - |
| Poaceae | *Poa alpina* | + | + |
| Poaceae | *sp 1 (Poaceae)* | + | - |
| Poaceae | *sp 2 (Poaceae)* | + | + |
| Poaceae | *sp 3 (Poaceae)* | - | + |
| Polygonaceae | *Bistorts macrophylla* | + | - |
| Polygonaceae | *Perscicaria sp. 1* | + | - |
| Polygonaceae | *Persicaria sp. 2* | + | - |
| Polygonaceae | *Rumex nepalensis* | + | - |
| Primulaceae | *Primula capitata* | + | - |
| Primulaceae | *Primula denticulata* | + | + |
| Primulaceae | *Primula dickeania* | + | - |
| Primulaceae | *Primula glabra* | + | - |
| Primulaceae | *Primula polifera* | + | - |
| Primulaceae | *Primula sikkimesis* | + | + |
| Primulaceae | *Primula sp gloerata* | + | - |
| Primulaceae | *sp. 1 (Primulaceae)* | - | + |
| Ranunculaceae | *Aconitum ferox* | - | + |
| Ranunculaceae | *Anemone rivularis* | - | + |
| Ranunculaceae | *Delphinium sp.* | + | + |
| Ranunculaceae | *Ranunculus tanguticus* | + | - |
| Rosaceae | *Fagaria sp. 1* | + | - |
| Rosaceae | *Fagaria sp. 2* | + | - |
| Rosaceae | *Potentilla sp. 1* | + | - |
| Rosaceae | *Potentilla sp. 2* | - | + |
| Rosaceae | *Potentilla sp. 3* | + | - |
| Rosaceae | *sp.1 (Rosaceae)* | + | - |
| Rosaceae | *sp.2 (Rosaceae)* | + | - |
| Rosaceae | *sp.3 (Rosaceae)* | - | + |
| Rosaceae | *sp.4 (Rosaceae)* | + | - |
| Rosaceae | *sp.5 (Rosaceae)* | + | + |
| Rosaceae | *sp.6 (Rosaceae)* | - | + |
| Rosaceae | *sp.7 (Rosaceae)* | + | + |
| Rosaceae | *sp.8 (Rosaceae)* | + | + |
| Rubiaceae | *sp. 1(Rubiaceae)* | - | + |
| Rubiaceae | *sp. 2(Rubiaceae)* | + | - |
| Rubiaceae | *sp. 3(Rubiaceae)* | - | + |
| Violaceae | *Viola biflora* | - | + |
| Zingerberaceae | *Roscoea pupurea* | - | + |
| Unidentified taxa 1 |  | - | + |
| Unidentified taxa 2 |  | - | + |
| Unidentified taxa 3 |  | - | + |
| Unidentified taxa 4 |  | + | - |
| Unidentified taxa 5 |  | + | - |
| Unidentified taxa 6 |  | + | - |
| Unidentified taxa 7 |  | + | - |
| Unidentified taxa 8 |  | + | - |
| Unidentified taxa 9 |  | - | + |
| Unidentified taxa 10 |  | + | - |
| Unidentified taxa 11 |  | - | + |
| Unidentified taxa 12 |  | - | + |
| Unidentified taxa 13 |  | + | - |
| Unidentified taxa 14 |  | - | + |
| Unidentified taxa 15 |  | - | + |
| Unidentified taxa 16 |  | + | + |
| Unidentified taxa 17 |  | - | + |
| Unidentified taxa 18 |  | + | - |
| Unidentified taxa 19 |  | + | + |
| Unidentified taxa 20 |  | + | - |
| Unidentified taxa 21 |  | + | - |
| Unidentified taxa 22 |  | + | - |
| Unidentified taxa 23 |  | + | + |
| Unidentified taxa 24 |  | + | - |
| Unidentified taxa 25 |  | + | - |
| Unidentified taxa 26 |  | + | - |
| Unidentified taxa 27 |  | + | + |
| Unidentified taxa 28 |  | + | - |
| Unidentified taxa 29 |  | + | - |
| Unidentified taxa 30 |  | + | - |
| Unidentified taxa 31 |  | - | + |
