# Supplementary material for: Pastoralism in the highest peaks: Role of the traditional grazing systems in maintaining biodiversity and ecosystem function in the alpine Himalaya
Source: PLoS One. 2021 Jan 7;16(1):e0245221. doi: 10.1371/journal.pone.0245221 (PMC7790420; doi:10.1371/journal.pone.0245221)
Supplement: Attachment — Submitted filename: PONE-D-20-24843_reviewer.pdf [file pone.0245221.s002.pdf]

PLOS ONE

Pastoralism in the highest peaks: Role of the traditional grazing systems in maintaining biodiversity and ecosystem function in the alpine Himalaya.

--Manuscript Draft--

**Manuscript Number:**	PONE-D-20-24843
**Article Type:**	Research Article
**Full Title:**	Pastoralism in the highest peaks: Role of the traditional grazing systems in maintaining biodiversity and ecosystem function in the alpine Himalaya.
**Short Title:**	Role of the traditional grazing systems in maintaining biodiversity and ecosystem function in alpine Himalaya.
**Corresponding Author:**	tenzing Ingty, MS<br>University of Massachusetts Boston<br>Boston, Massachusetts UNITED STATES
**Keywords:**	grazing ban policy; livestock grazing; species diversity; productivity; Sikkim; alpine pastures
**Abstract:**	Rangelands cover around half of the planet’s land mass and provide vital ecosystem services to over a quarter of humanity. The Himalayan rangelands, part of a global biodiversity hotspot is among the most threatened regions in the world. In rangelands of many developing nations policies banning grazing in protected areas is common practice. In 1998, the Indian state of Sikkim, in the Eastern Himalaya, enacted a grazing ban in response to growing anthropogenic pressure in pastures and forests that was presumably leading to degradation of biodiversity. Studies from the region demonstrate the grazing ban has had some beneficial results in the form of increased carbon stocks and regeneration of some species of conservation value but the ban also resulted in negative outcomes such as reduced household incomes, increase in monocultures in lowlands, decreased manure production in a state that exclusively practices organic farming, spread of gregarious species, and a perceived increase in human wildlife conflict. This paper explores the impact of the traditional pastoral system on high elevation plant species in Lachen valley, one of the few regions of Sikkim where the grazing ban was not implemented. The results demonstrate that grazing significantly contributes to greater plant species diversity (Species richness, Shannon index, Simpson diversity index, and Pielou evenness index) and ecosystem function (using above ground net primary productivity as an indicator). The multidimensional scaling and ANOSIM (Analysis of Similarities) pointed to significant differences in plant species assemblages in grazing and ungrazed areas. Further, ecosystem function is controlled by grazing, rainfall and elevation. Thus, the traditional transhumant pastoral system may enhance biodiversity and ecosystem function. I argue that a complete restriction of open grazing meet neither conservation nor socioeconomic goals. Evidence based policies are required to conserve the rich and vulnerable biodiversity of the region.
**Order of Authors:**	tenzing Ingty, MS
**Additional Information:**	
**Question**	**Response**
**Financial Disclosure**<br><br>Enter a financial disclosure statement that describes the sources of funding for the work included in this submission. Review the submission guidelines for detailed requirements. View published research articles from *PLOS ONE* for specific examples.	Field work was supported by funding from<br>1. Department of Biotechnology, Ministry of Science and Technology Government of India to Ashoka Trust for Research in Ecology and the Environment, Bangalore, India (atree.org)<br>2. ATREE Small Grants for research in Northeast India to TI<br>3. Sanofi Genzyme and Oracle Fellowship to TI<br><br>The funders had no role in study design, data collection and analysis, decision to publish, or preparation of the manuscript

Powered by Editorial Manager® and ProduXion Manager® from Aries Systems Corporation

This statement is required for submission and **will appear in the published article** if the submission is accepted. Please make sure it is accurate.<br><br>**Unfunded studies**<br>Enter: *The author(s) received no specific funding for this work.*<br><br>**Funded studies**<br>Enter a statement with the following details:<br>• Initials of the authors who received each award<br>• Grant numbers awarded to each author<br>• The full name of each funder<br>• URL of each funder website<br>• Did the sponsors or funders play any role in the study design, data collection and analysis, decision to publish, or preparation of the manuscript?<br>• **NO** - Include this sentence at the end of your statement: *The funders had no role in study design, data collection and analysis, decision to publish, or preparation of the manuscript.*<br>• **YES** - Specify the role(s) played.<br><br>* typeset	
**Competing Interests**<br><br>Use the instructions below to enter a competing interest statement for this submission. On behalf of all authors, disclose any competing interests that could be perceived to bias this work—acknowledging all financial support and any other relevant financial or non-financial competing interests.<br><br>This statement **will appear in the published article** if the submission is accepted. Please make sure it is accurate. View published research articles from *PLOS ONE* for specific examples.	No competing interests. This is a single author publicaiton.

**NO authors have competing interests**<br><br>Enter: *The authors have declared that no competing interests exist.*<br><br>**Authors with competing interests**<br><br>Enter competing interest details beginning with this statement:<br><br>*I have read the journal's policy and the authors of this manuscript have the following competing interests: [insert competing interests here]*<br><br>* typeset	
**Ethics Statement**<br><br>Enter an ethics statement for this submission. This statement is required if the study involved:<br><br>• Human participants<br>• Human specimens or tissue<br>• Vertebrate animals or cephalopods<br>• Vertebrate embryos or tissues<br>• Field research<br><br>Write "N/A" if the submission does not require an ethics statement.<br><br>General guidance is provided below. Consult the submission guidelines for detailed instructions. **Make sure that all information entered here is included in the Methods section of the manuscript.**	Field Research.<br>Permit number: 03592280402<br>Department of Forest, Environment & Wildlife Management, Government of Sikkim, India.

Powered by Editorial Manager® and ProduXion Manager® from Aries Systems Corporation

**Format for specific study types**<br><br>**Human Subject Research (involving human participants and/or tissue)**<br>• Give the name of the institutional review board or ethics committee that approved the study<br>• Include the approval number and/or a statement indicating approval of this research<br>• Indicate the form of consent obtained (written/oral) or the reason that consent was not obtained (e.g. the data were analyzed anonymously)<br><br>**Animal Research (involving vertebrate animals, embryos or tissues)**<br>• Provide the name of the Institutional Animal Care and Use Committee (IACUC) or other relevant ethics board that reviewed the study protocol, and indicate whether they approved this research or granted a formal waiver of ethical approval<br>• Include an approval number if one was obtained<br>• If the study involved *non-human primates*, add *additional details* about animal welfare and steps taken to ameliorate suffering<br>• If anesthesia, euthanasia, or any kind of animal sacrifice is part of the study, include briefly which substances and/or methods were applied<br><br>**Field Research**<br><br>Include the following details if this study involves the collection of plant, animal, or other materials from a natural setting:<br>• Field permit number<br>• Name of the institution or relevant body that granted permission	
**Data Availability**<br><br>Authors are required to make all data underlying the findings described fully available, without restriction, and from the time of publication. PLOS allows rare exceptions to address legal and ethical concerns. See the PLOS Data Policy and FAQ for detailed information.	Yes - all data are fully available without restriction

Powered by Editorial Manager® and ProduXion Manager® from Aries Systems Corporation

A Data Availability Statement describing where the data can be found is required at submission. Your answers to this question constitute the Data Availability Statement and **will be published in the article**, if accepted.<br><br>**Important:** Stating 'data available on request from the author' is not sufficient. If your data are only available upon request, select 'No' for the first question and explain your exceptional situation in the text box.<br><br>Do the authors confirm that all data underlying the findings described in their manuscript are fully available without restriction?	
**Describe where the data may be found in full sentences. If you are copying our sample text, replace any instances of XXX with the appropriate details.**<br><br>• If the data are **held or will be held in a public repository**, include URLs, accession numbers or DOIs. If this information will only be available after acceptance, indicate this by ticking the box below. For example: *All XXX files are available from the XXX database (accession number(s) XXX, XXX.).*<br>• If the data are all contained **within the manuscript and/or Supporting Information files**, enter the following: *All relevant data are within the manuscript and its Supporting Information files.*<br>• If neither of these applies but you are able to provide **details of access elsewhere**, with or without limitations, please do so. For example:<br><br>*Data cannot be shared publicly because of [XXX]. Data are available from the XXX Institutional Data Access / Ethics Committee (contact via XXX) for researchers who meet the criteria for access to confidential data.*<br><br>*The data underlying the results presented in the study are available from (include the name of the third party*	All relevant data are within the manuscript.

and contact information or URL).<br>• This text is appropriate if the data are owned by a third party and authors do not have permission to share the data.<br><br>* typeset	
Additional data availability information:	

Manuscript
Click here to access/download;Manuscript;Pastoralism in the highest peaks_MS.docx

Pastoralism in the highest peaks: Role of the traditional grazing systems in maintaining biodiversity and ecosystem function in the alpine Himalaya.

Tenzing Ingty

Department of Biology, University of Massachusetts, Boston, MA

tenzingingty@gmail.com

Abstract

Rangelands cover around half of the planet's land mass and provide vital ecosystem services to over a quarter of humanity. The Himalayan rangelands, part of a global biodiversity hotspot is among the most threatened regions in the world. In rangelands of many developing nations policies banning grazing in protected areas is common practice. In 1998, the Indian state of Sikkim, in the Eastern Himalaya, enacted a grazing ban in response to growing anthropogenic pressure in pastures and forests that was presumably leading to degradation of biodiversity. Studies from the region demonstrate the grazing ban has had some beneficial results in the form of increased carbon stocks and regeneration of some species of conservation value but the ban also resulted in negative outcomes such as reduced household incomes, increase in monocultures in lowlands, decreased manure production in a state that exclusively practices organic farming, spread of gregarious species, and a perceived increase in human wildlife conflict. This paper explores the impact of the traditional pastoral system on high elevation plant species in Lachen valley, one of the few regions of Sikkim where the grazing ban was not implemented. The results demonstrate that grazing significantly contributes to greater plant species diversity (Species richness, Shannon index, Simpson diversity index, and Pielou evenness index) and ecosystem function (using above ground net primary productivity as an indicator). The multidimensional scaling and ANOSIM (Analysis of Similarities) pointed to significant differences in plant species assemblages in grazing and ungrazed areas. Further, ecosystem function is controlled by grazing, rainfall and elevation. Thus, the traditional transhumant pastoral system may enhance biodiversity and ecosystem function. I argue that a complete restriction of open grazing meet neither conservation nor socioeconomic goals. Evidence based policies are required to conserve the rich and vulnerable biodiversity of the region.

Keywords

grazing ban policy, rangelands, species diversity, productivity, Himalaya, alpine pastures

Introduction

Rangelands cover approximately half of earth's land surface [1,2] and over a quarter of humanity depend on these land types for their livelihoods and numerous ecosystem services [3,4]. The ecosystem services include provisioning services like food, fiber and water, and supporting services such as carbon storage (roughly 30% of global soil carbon) among many others [5–8]. Rangelands are also recognized for their high biodiversity, including numerous endangered and endemic species, as well as habitat connectivity between protected forests [6,7,9,10]. In much of the developing world, rangelands are inhabited by pastoral communities. Pastoralism that has

existed for millennia has impacted the evolution of many rangelands, from the biodiverse rich African savanna [11] to the highest elevations in the Tibetan steppe [12]. Pastoralists and the rangelands they inhabit thus form complex socio-ecological systems that have co-evolved across different geographies, biodiversity and climates [11,13]. Many pastoralists have been blamed, often wrongly, for rangeland degradation [14,15] and targeted for "*modernization*" programs such as sedenterization. While some of these programs have been beneficial [6,16–18], most have shown to have socially and ecologically disastrous impacts resulting in a decline in wildlife, decreased grassland diversity and productivity and increased desertification [14,19,20]. Moreover, the abandonment of rangelands by pastoralists and climate change is leading to rapid shrub invasion, further increasing habitat loss [21,22]. Grazing by herbivores such as the semi domesticated reindeer has been shown to counteract the effect of warming on shrub expansion [23–26]. This suggests that grazing management is an important factor to maintain alpine areas in response to climate change driven shrub invasion.

Rangelands in the Himalaya, a global biodiversity hotspot [27], form the largest land use system and provides numerous ecosystem services to pastoral communities [28]. The Himalaya is the source of 10 of the largest rivers in Asia whose basins feed about one fifth of humanity. The alpine rangelands of the Himalaya thus play a vital role in retaining critical ecosystem services such as carbon sequestration, water storage and provisioning, and maintaining biodiversity, food security and livelihoods. Despite its importance a report on high altitude rangelands of the Himalaya [28] underline numerous threats such as invasive species, inappropriate management and development policies, overgrazing and climate change. Studies show that climate change has significantly impacted the Himalaya. These include an increase in average temperatures three times higher than the global average, altered phenology and shifts in distribution ranges of biodiversity [29–33]. Upward shifts in treelines and shrubs have led to woody ingression into alpine pastures [34–36].

The alpine rangelands of the tiny state of Sikkim in the Eastern Himalaya are among the most biodiverse regions in the world. They occupy just 0.006% of global alpine rangelands but harbor as much as 60% of the global alpine plant families and 10% (297 genera) of global alpine genera with more than twice the number of vascular plants than the Alps and Rockies [37]. In 1998 the government of Sikkim adopted a grazing exclusion policy in response to growing anthropogenic pressure on grazing lands and protected forests leading to perceived degradation. Open grazing was phased out over the next six years in all reserved forests with exception of Lachen and Lachung valley and alpine pastures in the Kangchenzonga National Park. The forest cover of the state between 1999 to 2017 increased by about 4% from 3118 km^2 to 3379 km^2. Studies in Barsey Rhododendron Sanctuary in West Sikkim estimate that twelve years after the grazing ban was declared carbon stocks increased by about 585 thousand tonnes and the regeneration of species of high conservation value such as *Rhododendron barbetum* [38,39]. At the same time, Bhagwat et al. [38] also warned of insignificant regeneration of important fodder species and other species of high conservation value such as *Quercus spp.* coupled with a spread of gregarious species such as *Arundinaria maling* and *Viburum cordifolium*. Restriction on free access to forest resources have had significant socio-economic impacts on the herders who were dependent on grazing pastures in the sanctuary. Their average net income from livestock was reduced by more than half the pre-ban period, with the source of majority of the incomes now

coming from government subsidies and schemes like the National Rural Employment Guarantee Act programme (MGNREGA) for unskilled labor [38,40]. A study on the perception of sedenterized ex-herders from West Sikkim identified loss of traditional livelihoods leading to decreased income and limited land holdings as their main protestations. In addition to socio-economic impacts to herders the grazing ban has had a cascading impact on non-herders[38]. Perceptions of local villagers indicate an increase in in human wildlife conflict in the form of crop raids and livestock depredation with over 85% of the households experiencing greater losses in agricultural outputs after the grazing ban [38,40].

Here, I explore the impact of the present pastoral system on high elevation plant species in Lachen valley, one of the few regions of Sikkim where the grazing ban was not implemented. The region is of significance for two reasons. First, it falls in one of the most biodiverse regions of the world, the alpine zone of the Sikkim Himalaya [37]. Second transhumant pastoralism has been practiced in the region for centuries with the indigenous communities directly dependent on the biodiversity of their surrounding for livestock husbandry, water storage and provisioning, and the collection of medicinal, aromatic, and edible plants [41–43].

I address 2 broad questions in the alpine pastures of the Himalaya.

1. What is the impact of the traditional grazing system on plant biodiversity and function in the high-altitude alpine Himalaya?
2. How does grazing maintain biodiversity and ecosystem function in the region?

I first analyze the impact of grazing on species diversity and productivity. I then discuss the role of grazing in maintaining high biodiversity and ecosystem function. Finally, I conclude by identifying further knowledge needs and propose recommendations for an improved grazing policy to protect the rich yet vulnerable biodiversity of the region.

The results demonstrate that grazing enhances species diversity and productivity indicating that the pastures in the region have benefited from grazing by livestock. The results are significant in two respects. First, the results suggest that a complete ban on grazing, a common practice in protected areas of developing countries [44–46] may not meet its primary objective of conserving biodiversity. Second, the socioeconomic losses because of the grazing ban can be reduced by reintroducing grazing after sufficient research on altered herd sizes and the impacts of specific livestock and grazing intensities. The results suggest that a management strategy that is mutually beneficial for conservation and livelihoods is not just possible but desirable to maintain higher local and regional biodiversity.

Materials and methods

Study area: The study was conducted in Lachen valley in the North district of the state of Sikkim in India. Sikkim, in the Eastern Himalayan region (fig. 1), is the second smallest (7096 km2) and the least populous state of India. The alpine zone of Sikkim has unique biodiversity since it also falls in the transition zone of 2 biogeographic regions, namely the central Himalaya region and the southern fringe of the Palearctic region [47]. Lachen valley lies within the alpine zone of Sikkim and shares an international border with the Tibet Autonomous Region (TAR) of China. The valley is among the few places where open grazing is permitted in the state of Sikkim. It is inhabited by two pastoral communities— the yak and sheep herders, the Dokpas

and the agro-pastoral Lachenpas. According to the states 2012 livestock census the communities rear 951 cattle, 2588 yaks, 141 horses, 230 sheep (indigenous variety) and 90 goats[48]. Historically both communities conducted trade and practiced pastoralism [49,50]. They used to take their yak and sheep upto Khambazong in TAR, China in summer and traded oil, sugar and fir planks for wool, barley flour (*tsampa*), salt, carpets, mutton and sheep fat. After the 1962 Sino-Indian war the international border was closed off. Today, both communities practice seasonal transhumance. The Lachenpas move to the high elevation pastures in the summer and retreat to lower altitudes in the winter, while the Dokpas do the opposite, moving onto the Tibetan Plateau where they find wind-blown, snow-free pastures in winter. Both communities rely on common property resources (pastures, forests, and natural waters) and some pastures on state forest department and army property.

Vegetation sampling: Plant species were sampled in grazing pastures and ungrazed areas. Grazed areas were defined as pastures most often in use and that have been grazed for atleast two decades. The ungrazed areas were defined as areas with a history of no grazing for ateast 10 years with permanent fences around them. These areas are used by the locals to make hay. The herbs and grass are left to grow in these areas throughout the year and removed at the end of the growing season. Local pastoralists and village representatives were consulted to select grazed and ungrazed areas. I followed a randomized block design with three replicates laid along an elevation gradient (lower elevation zone =3000-3500m; middle elevation zone =3500-400m; and higher elevation zone= 4000-4500m asl). Each block contained both grazed and ungrazed areas. Within each block a 30x30 m plot was demarcated. It was difficult to separate the effects of different grazers since there was much overlap in the grazing areas shared by the different livestock and wild species. Cows, bulls, goats, horses and a few yak shared the same pastures in the lower elevation zone (3000-3500 m asl). Sheep, yak, cows, bulls, goats, and horses shared the middle elevation zones (3500-4000 m asl) and yak, sheep and a few horses grazed in the higher elevation zone (4000-4500 m asl). Thus, I studied the combined effects of different grazing species.

Impacts of grazing on species diversity and above ground net primary productivity (ANPP)

In each 30X30 m plot 24 quadrates (1X1 m) were randomly laid (12 in grazed areas and 12 in ungrazed areas). Thus, total of 72 1x1 m quadrats with 24 quadrates were laid in each elevation zone. Data on species composition and percentage cover was collected from each quadrat.

I followed the difference method by McNaughton et al. [51] to calculate ANPP in the grazed area. Twelve temporary movable exclosure cages, 1x1m in size, were set up in the grazed area. After each sampling these cages were moved to a new grazed location to account for biomass growth (over a month). The standing biomass of was sampled inside and outside exclosure cages. The collected plant material was oven dried 24 hours at 60°C and then weighed. The ANPP of grazing plots was calculated using the following formula [51] $ANPP= W1g+(W2u-W1g)+(W3u-W2g)+(W4u-W3g)+(W5u-W4g)$

where Wi is the dry mass weight of standing biomass at sample time ti (i=1, 2, 3, 4, 5: beginning of June, July, August, September, October respectively). u (ungrazed) and g (grazed) represent samplings inside and outside the exclosure cages, respectively. In ungrazed areas twelve 1x1m quadrats were laid and biomass was collected at peak standing crop.

Data analysis: Diversity indices—species richness (S), shannon index (H) , simpson diversity index (D) and pielous's evenness (J) were calculated for plant species in grazed and ungrazed quadrates in each elevation zone and for all quadrates (all elevations and grazing treatments combined). Proportional dominance (PD) of a species was calculated as a function of relative density and frequency (cover). PD = (Ni/N) *fi

where PD is the proportional dominance of species i, Ni is the percent cover of species i in all locations, N is the percent cover of all species in all locations, and fi is the frequency of plots at which species i occurs. The Welch's two Sample t-test was used to calculate significance for difference between diversity indices and ANPP in grazed and ungrazed areas (table 1). An analysis of variance (ANOVA) was used to determine the effect of grazing and elevation on species diversity indices and ANPP (table 2). The ANOVA was conducted as a complete randomized block design with grazing treatment, elevation and their interaction taken as fixed effects. The Shapiro-Wilk test and Levene's test were used to check the residuals of analysis for normality and for homogeneity, respectively.

To explore the response of species composition to grazing treatments and elevation a triangular similarity matrix was constructed using Bray Curtis similarity coefficients based on cover data. The similarity matrix was displayed in an ordination plot using non-metric multidimensional scaling (NMDS). The stress function assesses how well the two-dimensional plot represent actual sample relationships calculated. A lower value (<0.2 commonly accepted) represents a more accurate ordination [52,53].
The Analysis of similarity (ANOSIM) was used to check the degree of dissimilarity of species across grazing treatments, elevation zones, and treatments within each elevation zone. ANOSIM based on the Bray Curtis similarity matrix was used to check significance levels using a permutation test with 999 simulations. R values close to 1 suggest sites within a group are similar to each other and dissimilar to sites in other groups. While an R value close to "0" suggests an even distribution of high and low ranks within and between groups. A linear regression was used to examine the relationship between diversity and productivity.
All analyses were performed on R. The NMDS and ANOSIM analysis were performed using the vegan function in R [54].

Results

Species diversity indices: A total of 153 species between 3000-4500 m asl were sampled. This included the 66,56 and 47 species found in the lower, middle, and higher elevation zones, respectively. A significantly ($p<0.001$) higher number of species was found in grazed areas (114 species) than in ungrazed areas (61 species). 51,48 and 30 species were sampled in grazed areas and 22,19 and 22 species in ungrazed areas in lower, middle, and higher elevation zones, respectively. The analysis showed all diversity indices (S,H,D and J) were significantly higher ($p<0.001$) in grazed quadrates than ungrazed quadrates in all elevation zones combined and in each elevation zone (fig 2A-D, table1). Grazing had a significant (ANOVA $p<0.001$)effect all diversity indices in each elevation zone and all elevation zones combined (table 2). Unlike grazing, elevation did not have a significant effect (ANOVA $p<0.05$)diversity indices (fig. 2G-I, table 2) except S (fig. 2F, table 2).

Results of the ANOSIM indicate statistically significant differences in species composition. There is a significant difference in species composition between grazing treatments within each elevation zone (Lower: R=0.0.911 p<0.001;mid: R= 0.786 p<0.001; higher R=0.878 p<0.001) (fig. 3). As would be expected species composition in different elevation zones showed a significant difference (R=0.844 p<0.001) (fig. 3). The ANOSIM R value for grazing treatment across all elevations was relatively lower (R=0.199 p<0.001) suggesting comparatively more overlap in species compositions in grazed and ungrazed areas (fig. 3). Overall, grazed and ungrazed plots had 14.38%(22) species in common. In the lower, middle and higher zones they shared 7(10.61%), 11 (19.64%) and 5 (10.64%) species, respectively.

Species proportional dominance: All plots were dominated by Graminoid species (Cyperaceae, Juncaceae and Poaceae). They represented 70.58%, 44.34% and 78.19% of PD in all quadrates combined, grazed quadrates and ungrazed quadrates, respectively. These included sixteen species (Cyperaceae n=7, Juncaceae n=2, Poaceae n=7) in total. In grazed quadrates I found thirteen graminoid species (Cyperaceae n=6, Juncaceae n=2, Poaceae n=5) and ten graminoid species (Cyperaceae n=4, Juncaceae n=1, Poaceae n=5) in ungrazed quadrates. Forbs showed high PD in grazed quadrates than ungrazed quadrates with Polygonaceae, Primulaceae, Rosaceae, Asteraceace and Orchidaceae families representing 37.75% of PD.

At the species level *Kobresia nepalensis* (Cyperaceae) was the most dominant species in grazed and ungrazed plots. The 5 most dominant species in all quadrates combined represented 55.95% of PD. In grazed and ungrazed quadrates the five most dominant species represented 48.09% and 61.18% of dominance, respectively. These included graminoid species in all quadrates combined and ungrazed plots. In grazed plots *Rumex nepalensis* and *Primula dendiculata* were the only forbs representing the five most dominant species the remaining 3 were represented by graminoid species *Kobresia nepalensis*, *Festuca coelestis* (Poaceae) and *Juncus sp* (Juncaceae).

ANPP: The mean ANPP in all quadrates combined was 756.46 g/m^2 (table 1). Productivity showed a decreasing trend with elevation with the maximum (829.77 g/m^2) in lower elevation zones and minimum (674.93 g/m^2) in the higher elevation quadrates (Fig 2E, table 1). The analysis for variance showed that elevation had a significant (ANOVA p<0.001) effect on ANPP (table 2). The mean ANPP for all grazed quadrates (all elevation zones combined), lower and middle elevation zone grazed quadrates was highest in July (Fig 2K) and in the higher elevation zone grazed quadrates in August (Fig 2K).

Productivity was significantly higher in grazed areas than in ungrazed areas (table 1). Overall, the average ANPP in grazed areas was 32.07 % higher than in ungrazed areas. Maximum ANPP (955.27 g/m^2) was found in quadrats laid in the grazed areas in the lower elevation zone and minimum (595.9 g/m^2) in ungrazed areas in the higher elevation zone (table 1). Grazing significantly increased ANPP by 35.64%, 33.28% and 26.52% in the lower, middle and higher elevation zones, respectively. The analysis showed that grazing had a statistically significant effect (ANOVA P<0.001) on productivity within each elevation zone and all elevation zones combined (table 2). The linear regression revealed a statistically significant ($p<0.001$, R^2= 0.457) relationship between species richness and ANPP (Fig 2L).

Discussion

The discussion is divided into three parts. The first two parts address the question how does grazing maintain high species diversity and ecosystem function. First, I discuss how grazing may be a driver for higher species diversity. The second part discusses the role of species diversity on productivity. Finally, I discuss the socioecological dynamics in the region that have resulted in fewer families investing in pastoralism.

Impact of grazing on species diversity and ANPP: The results demonstrate the importance of the traditional grazing system in maintaining high diversity and productivity. Significantly higher diversity indices and productivity in grazed areas suggest plant community composition, structure and function benefit or depend indirectly on grazing for their persistence in the high-altitude alpine pastures of the Himalaya. Grazing may be a driver for higher species diversity in three ways.

First, in free grazing systems like in the study site, higher diversity could be facilitated by the important role livestock play as seed dispersers. Seed dispersal by grazing animals is important in grasslands throughout the world, being vital for conservation of not only species but genetic diversity [55–57]. Grazing animals are dispersal vectors through epizoochory (transporting outside the body e.g. on fur) or endozoochory (transporting after ingestion). Studies have found higher survival of small seeds in the digestive tract of mammals increasing effectiveness of endozoochory [58–60]. This could explain higher PD of small seeded less palatable species such as Orchidaceae and Asteraceae (especially *Anaphalis lactea* and *Lentopodium leontopodioides)* in grazed plots.

Second, grazing results in the reduction in abundance of dominant species by selectively removing palatable species [61]. This explains higher PD for graminoid species (that are palatable) in ungrazed areas. The results show that graminoid species represent 78.19% of the total PD in ungrazed quadrates as compared to 44.34% in grazed quadrates. Additionally, higher PD of less palatable families such as Asteraceace, Primulaceae and Rosaceae (dominated by *Potentilla* spp) was found in grazed plots.

Third, grazing can increase spatial heterogeneity thus increasing habitat diversity. Free ranging livestock may modify soil properties directly by recycling nutrients and concentrating it from a larger area into small patches through their dung and urine deposition [62–65]. Grazing may also affect soil properties indirectly through their forage preference and grazing intensity. This may selectively affect the survival of some plants that affect soil properties through their rhizosphere [66,67].

Grazing mediated dispersal, foraging preferences and spatial heterogeneity would increase niche breadth and overlap and may promote patches of rarer species thus increasing diversity.

A strong positive correlation between productivity and rainfall has been well documented across the globe [63,68–70]. In the study site monthly productivity in grazed plots were closely associated with rainfall. The monsoon season in the region starts in May, peaking in July and gradually ending in September and October. Peak ANPP in lower and middle elevation quadrates

was reached in July and gradually tapered off with the receding monsoon winds. The monsoons gradually travels northwards, towards the higher elevation explaining why the higher elevation quadrates showed a slight delay in peak ANPP (in August). Interestingly the migration pattern of the local communities is closely associated to temporal trends in productivity with the Lachenpas moving up to the lower and middle elevation zones in June and July and the Dokpas moving down to the higher elevation zones around mid-July. Higher ANPP in grazed plots suggests grazing mediated species diversity is an important driver of ANPP.

Relationship between species diversity and ANPP: A strong positive correlation between diversity and productivity (fig 2L) suggests higher biodiversity is an important factor in driving ecosystem functions. The results demonstrating that biomass production increased with diversity conforms to numerous studies from other parts of the world [71–74]. There are three explanations proposed based on experimental studies to understand the role of diversity on productivity. Firstly, complementarity effects occur when more diverse communities facilitate greater niche partitioning allowing for a more complete utilization of resources resulting in increased biomass production [71,73,75]. Secondly, facilitative effects of some species promote the establishment and survival of other co-occurring species influencing biomass production [72,76]. Thirdly, sampling or selection effects suggesting that more diverse plots have a higher probability of containing species with high biomass[72,74,77,78]. The three effects are not mutually exclusive, rather a combined influence of complementarity, facilitative and selection effect of biodiversity influence biomass in a community.

Studies on the impact of grazing on ecosystems have shown mixed results. Negative effects of grazing have been attributed to high grazing intensity owing to overgrazing and high stocking rates or to low grazing intensity including the abandonment of grazing [2,21,22,63,79,80]. The general consensus is that a light to medium grazing intensity is most beneficial [2,63,81]. This study demonstrates that the pastures in Lachen valley have benefitted from grazing of various livestock suggesting the stocking rates are within or below "carrying capacity". The traditional rotational grazing system practiced by the communities allow for pasture regrowth and recovery, facilitates resilience and limits degradation. Similar results have been shown by studies from other regions underlining the benefits of rotational grazing [82–85].

Socio ecological systems: Driven by external constraints and the benefits of urbanization, the local communities have diversified toward other sectors, particularly the tourism industry and as porters for the Indian army. The most immediate reason for this change was the complete closure of the international border after the war of 1962 between India and China. This not only ended trade with Tibet, but also led to a loss of vast areas of pastures in TAR. In addition to this the establishment of large army encampments and areas for land mines further reduced areas for grazing to an estimated 40% of the original area [86]. Previously, the transboundary migration also ensured the interaction of a larger pool of pastoral people resulting in intermarriages from a larger region as well as more opportunities to cross breed livestock. Today, most Dokpa children are sent to schools in the city and few return to the rigors of a pastoralist's life. Some Dokpas prefer working as porters and laborers for the Indian army stationed in the region or in the tourism industry as guides, porters, and taxi drivers. Lachenpas too are investing more into the growing tourism industry and less into animal husbandry that show diminishing monetary returns. The loss of grazing pastures and access to trade, reduced opportunities for marriage and cross-breeding livestock, and the exposure to modern education and urbanization have resulted

in a reduction in Dokpa population living in the region from 30 households (130 persons) in the 1950's [86,87] to fewer than a dozen families today. Fewer Dokpas and Lachenpas investing in pastoralism would eventually result in lesser livestock numbers.

Conclusion

The crucial role of the traditional grazing system on biodiversity and ecosystem function, coupled with disinvestment in pastoralism by the locals poses an imminent threat to the landscape that is an intricate socio-ecological system with a long history of co-adaptation with ruminant animal. Pastoralism is associated with other spillover effects such as illegal timber and fuelwood collection, medicinal plants (often rare and endemic) collection and the negative effects of overgrazing as enumerated by Bhagwat et al. [38]. At the same time this study shows, the absence of grazing may result in loss of biodiversity and reduced ecosystem function. The benefits of the traditional free ranging pastoralism cannot be ignored, and a holistic approach is needed to conserve the rich biodiversity of the region. As studies from the region demonstrate the grazing ban has resulted in reduced incomes [38], increase in monocultures in lowlands, decreased manure production in a state that exclusively practices organic farming [88], spread of gregarious species and perceived increase in human wildlife conflict [38,40].
I conclude with research and policy recommendations that should be part of an improved grazing management policy in the state.

1. Experimental studies reintroducing grazing in small areas in regions where the grazing ban was enacted need to be conducted. Studies with the aim to understand the role of different livestock species and different grazing intensities on biodiversity structure and function need to be conducted.
2. Climate change is among the biggest threats to Himalayan rangelands, a region of immense significance for ecosystem services provided to atleast one fifth of humanity and among the most among the most biodiverse in the world [34,37]. Further research on the role of grazing on combating shrub encroachment in response to climate change that threatens habitat loss is imperative.
3. The present grazing ban policy needs to be revisited with a more holistic approach
    a. To understand the role of altered herd sizes on diversity and ecosystem function. A complete ban of grazers meets neither conservation nor livelihood goals. A comprehensive approach taking into account social, ecological, and economic strategies would be required to meet sustainable goals.
    b. Explore a payment for ecosystem services schemes for the services provided by pastoralists in maintaining biodiversity and ecosystem function. These schemes should be aimed at pastoralists moving away from practicing traditional pastoralism. Similar schemes have been used in Switzerland where "*general direct payments*" are made for upland farmers to encourage grazing livestock in more difficult conditions [89].
    c. Explore the link between the grazing ban policy and increasing incidences of human wildlife conflict as reported by Bhagwat et al. [38]. An efficient compensation

scheme for human wildlife conflict that is affecting marginalized communities needs to be developed.

d. Enact reforms to improve livelihoods of ex-herders who are products of the grazing ban policy. While the government has made efforts to reduce this economic impact yet Gupta et al. [88] report frustration among ex-herders in response to "*a lack of reform to improve ex-herders' livelihoods*" post ban.

Acknowledgements

I would like to thank Dr. Kamaljit Bawa for offering vital comments and edits and Dr. Mahesh Sankaran for his advice on the experimental design. Dharmendra Lamsal assisted with parts of the data collection. Field work was supported by funding from Department of Biotechnology Government of India, ATREE Small Grants for research in Northeast India and Sanofi Genzyme and Oracle Fellowship.

References

1. Williams RE, Allred BW, DeNio RM, Paulsen Jr HA. Conservation, Development, and Use of the World's Rangelands. J Range Manag. 1968;21: 355–360. Available: https://www.jstor.org/stable/pdf/3896081.pdf

2. Zhang R, Wang Z, Han G, Schellenberg MP, Wu Q, Gu C. Grazing induced changes in plant diversity is a critical factor controlling grassland productivity in the Desert Steppe, Northern China. Agric Ecosyst Environ. 2018;265: 73–83. doi:10.1016/J.AGEE.2018.05.014

3. Havstad KM, Peters DC, Allen-Diaz B, Bartolome J, Bestelmeyer BT, Briske D, et al. The western United States rangelands, a major resource. In: Wedin WF, Fales SL, editors. Grassland Quietness and Strength for a New American Agriculture. Madison, WI: American Society of Agronomy Inc., Crop Science Society of America Inc., and Soil Science Society of America Inc; 2009. pp. 75–93.

4. Neely C, Bunning S, Wilkes A. Review of evidence on drylands pastoral systems and climate change. Rome: Food and Agriculture Organization of the United Nations, 2009; 2009. Available: http://citeseerx.ist.psu.edu/viewdoc/download?doi=10.1.1.469.5069&rep=rep1&type=pdf

5. Costanza R, d'Arge R, de Groot R, Farber S, Grasso M, Hannon B, et al. The value of the world's ecosystem services and natural capital. Nature. 1997;387: 253–260. doi:10.1038/387253a0

6. Sayre NF, McAllister RR, Bestelmeyer BT, Moritz M, Turner MD. Earth Stewardship of rangelands: coping with ecological, economic, and political marginality. Front Ecol Environ. 2013;11: 348–354. doi:10.1890/120333

7. Farley KA, Walsh K, Levine AS. Opportunities and obstacles for rangeland conservation in San Diego County, California, USA. Ecol Soc. 2017;22: 38.

8. Sala OE, Yahdjian L, Havstad K, Aguiar MR. Rangeland ecosystem services: nature's supply and humans' demand. In: Briske DD, editor. Rangeland systems: Processes,

management and challenges. Springer International Publishing, Cham; 2017. pp. 467–489.

9. Brunson MW, Huntsinger L. Ranching as a Conservation Strategy: Can Old Ranchers Save the New West? Rangel Ecol Manag. 2008;61: 137–147. doi:10.2111/07-063.1

10. Alkemade R, Reid RS, van den Berg M, de Leeuw J, Jeuken M. Assessing the impacts of livestock production on biodiversity in rangeland ecosystems. Proc Natl Acad Sci U S A. 2013;110: 20900–5. doi:10.1073/pnas.1011013108

11. Little PD. Pastoralism, biodiversity, and the shaping of savanna landscapes in East Africa. Africa (Lond). 1996;66: 37–51. doi:10.2307/1161510

12. Miller DJ. Nomads of the Tibetan Plateau Rangelands in Western China. Part One: Pastoral History. Rangelands. 1998;20: 24–29. Available: https://www-jstor-org.ezproxy.lib.umb.edu/stable/4001402?seq=1#metadata_info_tab_contents

13. Niamir-Fuller M, Huber-Sannwald E. Pastoralism and Achievement of the 2030 Agenda for Sustainable Development: A Missing Piece of the Global Puzzle. In: Lucatello S, Huber-Sannwald E, Espejel I, Martínez-Tagüeña N, editors. Stewardship of Future Drylands and Climate Change in the Global South. Springer, Cham; 2020. pp. 41–55. doi:10.1007/978-3-030-22464-6_3

14. Niamir-Fuller M. Managing mobility in African rangelands. Niamir-Fuller M, editor. London, UK: Intermediate Technology Publications; 1999.

15. Goldman M, Nadasdy P, Turner M, editors. Knowing nature: conversations at the intersection of political ecology and science studies. Chicago, IL: University of Chicago Press; 2011.

16. Sandford S, Scoones I. Improving the efficiency of opportunism: new directions for pastoral development. In: Scoones I, editor. Living with uncertainty: new directions in pastoral development in Africa. London, UK: Intermediate Technology Publications; 1994.

17. Banks T. Property Rights Reform in Rangeland China: Dilemmas On the Road to the Household Ranch. World Dev. 2003;31: 2129–2142. doi:10.1016/J.WORLDDEV.2003.06.010

18. Liu X, Ma Z, Huang X, Li L. How does grazing exclusion influence plant productivity and community structure in alpine grasslands of the Qinghai-Tibetan Plateau? Glob Ecol Conserv. 2020;23: e01066. doi:10.1016/J.GECCO.2020.E01066

19. Western D, Groom R, Worden J. The impact of subdivision and sedentarization of pastoral lands on wildlife in an African savanna ecosystem. Biol Conserv. 2009;142: 2538–2546. doi:10.1016/J.BIOCON.2009.05.025

20. Weber KT, Horst S. Desertification and livestock grazing: The roles of sedentarization, mobility and rest. Pastor Res Policy Pract. 2011;1: 19. doi:10.1186/2041-7136-1-19

21. Camacho O, Dobremez L, Capillon A. Shrub encroachment in pastures in the Alps. Rev géographie Alp. 2008; 89–100. doi:10.4000/rga.575

22. Zhang W, Miller PA, Smith B, Wania R, Koenigk T, Döscher R. Tundra shrubification and tree-line advance amplify arctic climate warming: results from an individual-based dynamic vegetation model. Environ Res Lett. 2013;8: 034023. doi:10.1088/1748-9326/8/3/034023

23. Post E, Pedersen C. Opposing plant community responses to warming with and without herbivores. Proc Natl Acad Sci U S A. 2008;105: 12353–8. doi:10.1073/pnas.0802421105

24. Olofsson J, Oksanen L, Callaghan T, Hulme P., Oksanen T, Suominen O. Herbivores inhibit climate-driven shrub expansion on the tundra. Glob Chang Biol. 2009;15: 2681–2693. doi:10.1111/j.1365-2486.2009.01935.x

25. Myers-Smith IH, Forbes BC, Wilmking M, Hallinger M, Lantz T, Blok D, et al. Shrub expansion in tundra ecosystems: dynamics, impacts and research priorities. Environ Res Lett. 2011;6: 045509. doi:10.1088/1748-9326/6/4/045509

26. Verma M, Schulte to Bühne H, Lopes M, Ehrich D, Sokovnina S, Hofhuis SP, et al. Can reindeer husbandry management slow down the shrubification of the Arctic? J Environ Manage. 2020;267: 110636. doi:10.1016/J.JENVMAN.2020.110636

27. Mittermeier RA, Turner WR, Larsen FW, Brooks TM, Gascon C. Global Biodiversity Conservation: The Critical Role of Hotspots. In: Zachos FE, Habel JC, editors. Biodiversity Hotspots: Distribution and Protection of Conservation Priority Areas. Berlin, Heidelberg: Springer Berlin Heidelberg; 2011. pp. 3–22. doi:10.1007/978-3-642-20992-5_1

28. Ning W, Rawat G, Joshi S, Ismail M, Sharma E, editors. High-altitude rangelands and their interfaces in the Hindu Kush Himalayas. Kathmandu: ICIMOD; 2013.

29. Shrestha UB, Gautam S, Bawa KS. Widespread climate change in the Himalayas and associated changes in local ecosystems. PLoS One. 2012;7: e36741. doi:10.1371/journal.pone.0036741

30. Telwala Y, Brook BW, Manish K, Pandit MK. Climate-induced elevational range shifts and increase in plant species richness in a Himalayan biodiversity epicentre. PLoS One. 2013;8: e57103. doi:10.1371/journal.pone.0057103

31. Pandey R, Khadka KK, Ghimire A, Jha P, Pathak U. Elevational distribution of butterflies in the Himalayas: a case study from Langtang National Park, Nepal. J Mt Sci. 2017;14: 1384–1390. doi:10.1007/s11629-017-4360-9

32. Tiwari A, Fan Z-X, Jump AS, Li S-F, Zhou Z-K. Gradual expansion of moisture sensitive Abies spectabilis forest in the Trans-Himalayan zone of central Nepal associated with climate change. Dendrochronologia. 2017;41: 34–43. doi:10.1016/J.DENDRO.2016.01.006

33. Anderson K, Fawcett D, Cugulliere A, Benford S, Jones D, Leng R. Vegetation expansion in the subnival Hindu Kush Himalaya. Glob Chang Biol. 2020; gcb.14919. doi:10.1111/gcb.14919

34. Xu J, Grumbine RE, Shrestha A, Eriksson M, Yang X, Wang Y, et al. The melting Himalayas: cascading effects of climate change on water, biodiversity, and livelihoods.

Conserv Biol. 2009;23: 520–30. doi:10.1111/j.1523-1739.2009.01237.x

35. Singh C., Paniprahy S, Thapliyal A, Kimothi M., Soni P, Parihar J. Monitoring the alpine treeline shift in parts of the Indian Himalayas using remote sensing. Curr Sci. 2012;102: 559–562.

36. Gaire N., Koirala M, Bhuju D., Borgaonkar H. Treeline dynamics with climate change at the central Nepal Himalaya. Clim Past. 2014;10: 1277–1290. Available: http://moeseprints.incois.gov.in/876/1/Treeline dynamics.pdf

37. Telwala Y. Climate change and alpine flora in Sikkim Himalaya. Climate Change in Sikkim - Patterns, Impacts and Initiatives. 2012. pp. 103–124.

38. Bhagwat S, Diwan M, Venkataramani V. Study of ecological, eocio-economic and livelihood dimensions of grazing exclusion in preotected forests of West Sikkim. Chennai; 2012.

39. Bhagwat S, Diwan M, Venkataramani V. Analysis of Grazing Exclusion Policy through a Climate Change Mitigation Lens: Case Studies from Barsey Rhododendron Sanctuary,West Sikkim. In: Arrawatia ML, Tambe S, editors. Climate Change in Sikkim - Patterns, Impacts and Initiatives. Gangtok: Information and Public Relations Department Government of Sikkim; 2012. pp. 351–368. Available: http://www.sikkimforest.gov.in/climate-change-in-sikkim/21-Chapter_Analysis of Grazing Exclusion Policy through the climate change.pdf

40. Tambe S, Bhutia N, Arrawatia M. People's opinion on the Impacts of "Ban on Grazing" in Barsey Rhododendron Sanctuary. Sikkim, India. Gangtok, Sikkim; 2005.

41. Singh P, Chauhan A. Plant Diversity in Sikkim Himalaya. In: Hajra P, Mugda V, editors. Plant Diversity and Hotspots in India. Calcutta: BSI; 1997. pp. 137–162.

42. Tambe S, Rawat GS. Ecology, Economics, and Equity of the Pastoral Systems in the Khangchendzonga National Park, Sikkim Himalaya, India. Ambio. 2009;38: 95–100.

43. Ingty T, Bawa KS. Climate change and indigenous people. In: Arrawatia ML, Tambe S, editors. Climate Change in Sikkim Patterns, Impacts and Initiatives. Information and Public Relations Department,Government of Sikkim, Gangtok; 2012. pp. 275–290.

44. Lewis M. Cattle and Conservation at Bharatpur: A Case Study in Science and Advocacy. Conserv Soc. 2003;1: 1–21. Available: https://www.jstor.org/stable/26396448?seq=1#metadata_info_tab_contents

45. Dong SK, Gao HW, Xu GC, Hou XY, Long RJ, Kang MY, et al. Farmer and professional attitudes to the large-scale ban on livestock grazing of grasslands in China. Environ Conserv. 2007;34: 246–254. Available: http://www.jstor.org/stable/44521238

46. Gurung B, Nelson KC, Smith JLD. Impact of Grazing Restrictions on Livestock Composition and Husbandry Practices in Madi Valley, Chitwan National Park, Nepal. Environ Conserv. 2009;36: 338–347. Available: https://www.jstor.org/stable/44520031?seq=1#metadata_info_tab_contents

47. Rodgers WA, Panwar HS. Planning a wildlife protected area network in India. Wildlife

Institute of India, Dehradun; 1988. Available: http://agris.fao.org/agris-search/search.do?recordID=XF9093847

48. Government of Sikkim. 19th Livestock Census, 2012. II. Gangtok, Sikkim; 2012. Available: http://www.sikkim-ahvs.gov.in/Download/Report2.pdf

49. Ingty T. High mountain communities and climate change: adaptation, traditional ecological knowledge, and institutions. Clim Change. 2017;145: 41–55. doi:10.1007/s10584-017-2080-3

50. Joshi S, Shrestha L, Bisht N, Wu N, Ismail M, Dorji T, et al. Ethnic and Cultural Diversity amongst Yak Herding Communities in the Asian Highlands. Sustainability. 2020;12: 957. doi:10.3390/su12030957

51. McNaughton SJ, Milchunas DG, Frank DA. How can net Primary Productivity be Measured in Grazing Ecosystems? Ecology. 1996;77: 974–977. doi:10.2307/2265518

52. Tyler CL, Kowalewski M. Utility of Marine Benthic Associations as a Multivariate Proxy of Paleobathymetry: A Direct Test from Recent Coastal Ecosystems of North Carolina. PLoS One. 2014;9: e95711. doi:10.1371/JOURNAL.PONE.0095711

53. Dexter E, Rollwagen-Bollens G, Bollens SM. The trouble with stress: A flexible method for the evaluation of nonmetric multidimensional scaling. Limnol Oceanogr Methods. 2018;16: 434–443. doi:10.1002/lom3.10257

54. Oksanen J, Blanchet FG, Kindt R, Legendre P, Minchin P., O'Hara R., et al. Vegan: Community Ecology Package. R Package Version 2.2-0. 2014.

55. Janzen DH. Dispersal of Small Seeds by Big Herbivores: Foliage is the Fruit. Am Nat. 1984;123: 338–353. Available: http://copa.acguanacaste.ac.cr:8080/bitstream/handle/11606/1223/DISPERSAL OF SMALL SEEDS BY BIG HERBIVORES FOLIAGE IS THE FRUIT.pdf?sequence=1

56. Couvreur M, Christiaen B, Kris V, Martin V. Large herbivores as mobile links between isolated nature reserves through adhesive seed dispersal. Appl Veg Sci. 2004;7: 229–236. Available: https://www.biw.kuleuven.be/lbh/lbnl/ecology/pdf-files/pdf-art/martine/Couvreuretal_Epizoochory_2004_AVS7 1 .229-236.pdf

57. Plue J, Aavik T, Cousins SAO. Grazing networks promote plant functional connectivity among isolated grassland communities. van Kleunen M, editor. Divers Distrib. 2019;25: 102–115. doi:10.1111/ddi.12842

58. Pakeman RJ, Digneffe G, Small JL. Ecological Correlates of Endozoochory by Herbivores. Funct Ecol. 2002;16: 296–304. Available: http://www.jstor.org/stable/826582

59. Cocyns E, Delporte A, Lens L, Hoffman M. Germination success of temperate grassland species after passage through ungulate and rabbit guts. J Ecol. 2005;93: 353–361. doi:10.1111/j.0022-0477.2005.00982.x

60. Wu G-L, Li X-P, Cheng J-M, Wei X-H, Sun L. Grazing Disturbances Mediate Species Composition of Alpine Meadow Based on Seed Size. Isr J Ecol Evol. 2009;55: 369–379. doi:10.1560/IJEE.55.4.369

61. Lu X, Kelsey KC, Yan Y, Sun J, Wang X, Cheng G, et al. Effects of grazing on ecosystem structure and function of alpine grasslands in Qinghai-Tibetan Plateau: a synthesis. Ecosphere. 2017;8: e01656. doi:10.1002/ecs2.1656

62. Carran RA, Ball PR, Theobald PW, Collins MEG. Soil nitrogen balances in urine-affected areas under two moisture regimes in Southland. New Zeal J Exp Agric. 1982;10: 377–381. doi:10.1080/03015521.1982.10427902

63. McNaughton SJ. Ecology of a Grazing Ecosystem: The Serengeti. Ecol Monogr. 1985;55: 259–294. doi:10.2307/1942578

64. Steinauer EM, Collins SL. Effects of Urine Deposition on Small-Scale Patch Structure in Prairie Vegetation. Ecology. 1995;76: 1195–1205. doi:10.2307/1940926

65. Liu C, Song X, Wang L, Wang D, Zhou X, Liu J, et al. Effects of grazing on soil nitrogen spatial heterogeneity depend on herbivore assemblage and pre-grazing plant diversity. Wan S, editor. J Appl Ecol. 2016;53: 242–250. doi:10.1111/1365-2664.12537

66. Hamilton EW, Frank DA. Can plants simulate soil microbes and their own nutrient supply? Evidence from a grazing tolerant grass. Ecology. 2001;82: 2397–2402. doi:10.1890/0012-9658(2001)082[2397:CPSSMA]2.0.CO;2

67. Wilson CH, Strickland MS, Hutchings JA, Bianchi TS, Flory SL. Grazing enhances belowground carbon allocation, microbial biomass, and soil carbon in a subtropical grassland. Glob Chang Biol. 2018;24: 2997–3009. doi:10.1111/gcb.14070

68. Pandey CB, Singh JS. Rainfall and Grazing Effects on Net Primary Productivity in a Tropical Savanna, India. Ecology. 1992;73: 2007–2021. doi:10.2307/1941451

69. Fay PA, Carlisle JD, Knapp · Alan K., Blair JM, Collins SL. Productivity responses to altered rainfall patterns in a C4-dominated grassland. Oecologia. 2003;137: 245–251. Available: https://www.researchgate.net/profile/John_Blair2/publication/10674171_Productivity_responses_to_altered_rainfall_patterns_in_a_C_4_-dominated_grassland/links/0fcfd50855628aff47000000.pdf

70. Castro SM, Sanchez-Azofeifa GA, Sato H. Effect of drought on productivity in a Costa Rican tropical dry forest. Environ Res Lett. 2018;13: 045001. Available: https://iopscience.iop.org/article/10.1088/1748-9326/aaacbc/pdf

71. Hector A. The Effect of Diversity on Productivity: Detecting the Role of Species Complementarity. Oikos. 1998;82: 597. doi:10.2307/3546380

72. Kelemen A, Török P, Valkó O, Deák B, Tóth K, Tóthmérész B. Both facilitation and limiting similarity shape the species coexistence in dry alkali grasslands. Ecol Complex. 2015;21: 34–38. doi:10.1016/J.ECOCOM.2014.11.004

73. Guerrero-Ramírez NR, Craven D, Reich PB, Ewel JJ, Isbell F, Koricheva J, et al. Diversity-dependent temporal divergence of ecosystem functioning in experimental ecosystems. Nat Ecol Evol. 2017;1: 1639–1642. doi:10.1038/s41559-017-0325-1

74. Sonkoly J, Kelemen A, Valkó O, Deák B, Kiss R, Tóth K, et al. Both mass ratio effects

and community diversity drive biomass production in a grassland experiment. Sci Rep. 2019;9: 1848. doi:10.1038/s41598-018-37190-6

75. Reich PB, Tilman D, Isbell F, Mueller K, Hobbie SE, Flynn DFB, et al. Impacts of Biodiversity Loss Escalate Through Time as Redundancy Fades. Science (80-). 2012;336: 589 LP – 592. doi:10.1126/science.1217909

76. Stachowicz JJ. Mutualism, Facilitation, and the Structure of Ecological CommunitiesPositive interactions play a critical, but underappreciated, role in ecological communities by reducing physical or biotic stresses in existing habitats and by creating new habitats on which many species depend. Bioscience. 2001;51: 235–246. doi:10.1641/0006-3568(2001)051[0235:mfatso]2.0.co;2

77. Huston MA. Hidden treatments in ecological experiments: re-evaluating the ecosystem function of biodiversity. Oecologia. 1997;110: 449–460. doi:10.1007/s004420050180

78. Loreau M. Separating Sampling and Other Effects in Biodiversity Experiments. Oikos. 1998;82: 600–602. Available: https://www.jstor.org/stable/pdf/3546381.pdf?casa_token=Tyj4lVMsMtYAAAAA:nQeur7AJMI64xit1hB_n_XPixj3AEELNPF4PmPgtHD1KvXtkMnKTZgAfh-FSOZ4MYlzvwdkrfc8FvwFn323FiQNq8r_WKQtDz7BvpaM_M9MS8pyeWA

79. Haynes MA, Fang Z, Waller DM. Grazing impacts on the diversity and composition of alpine rangelands in Northwest Yunnan. J Plant Ecol. 2013;6: 122–130. doi:10.1093/jpe/rts021

80. Li X, Liu Z, Wang Z, Wu X, Li X, Hu J, et al. Pathways of Leymus chinensis Individual Aboveground Biomass Decline in Natural Semiarid Grassland Induced by Overgrazing: A Study at the Plant Functional Trait Scale. Wang W, editor. PLoS One. 2015;10: e0124443. doi:10.1371/journal.pone.0124443

81. Pulungan MA, Suzuki S, Gavina MKA, Tubay JM, Ito H, Nii M, et al. Grazing enhances species diversity in grassland communities. Sci Rep. 2019;9: 11201. doi:10.1038/s41598-019-47635-1

82. Brundage AL, Petersen WE. A Comparison Between Daily Rotational Grazing and Continuous Grazing. J Dairy Sci. 1952;35: 623–630. doi:10.3168/jds.S0022-0302(52)93747-8

83. Teague WR, Dowhower SL. Patch dynamics under rotational and continuous grazing management in large, heterogeneous paddocks. J Arid Environ. 2003;53: 211–229. doi:10.1006/JARE.2002.1036

84. Vecchio MC, Bolaños VA, Golluscio RA, Rodríguez AM. Rotational grazing and exclosure improves grassland condition of the halophytic steppe in Flooding Pampa (Argentina) compared with continuous grazing. Rangel J. 2019;41: 1–12. Available: https://doi.org/10.1071/RJ18016

85. Jacobo EJ, Rodríguez AM, Bartoloni N, Deregibus VA. Rotational Grazing Effects on Rangeland Vegetation at a Farm Scale. Rangel Ecol Manag. 2006;59: 249–257. doi:10.2111/05-129R1.1

86. Sharma G, Tambe S, Rawat GS, Arrawatia M. Yak Herding and Associated Transboundary Issues in the Sikkim Himalaya, India. Transboundary challenges and opportunities for yak raising in a changing Hindu Kush Himalayan region. Kathmandu, Nepal: ICIMOD; 2016. pp. 93–112.

87. NBSAP. Sikkim State Biodiversity Strategy and Action Plan. Gangtok, Sikkim; 2002.

88. Gupta R, Haider LJ, Österblom H. The theory of cross-scale interactions: an illustration from remote villages in Sikkim, India. Environ Dev Sustain. 2020;22: 3777–3804. doi:10.1007/s10668-019-00329-0

89. Swiss Federal Office for Agriculture. Swiss Agricultural Policy: Objectives, tools, prospects. Bern, Switzerland; 2004. Available: https://www.cbd.int/financial/pes/swiss-pesagriculturalpolicy.pdf

Click here to access/download;Figure;Pastoralism in the highest peaks_Figures&tables with titles.docx

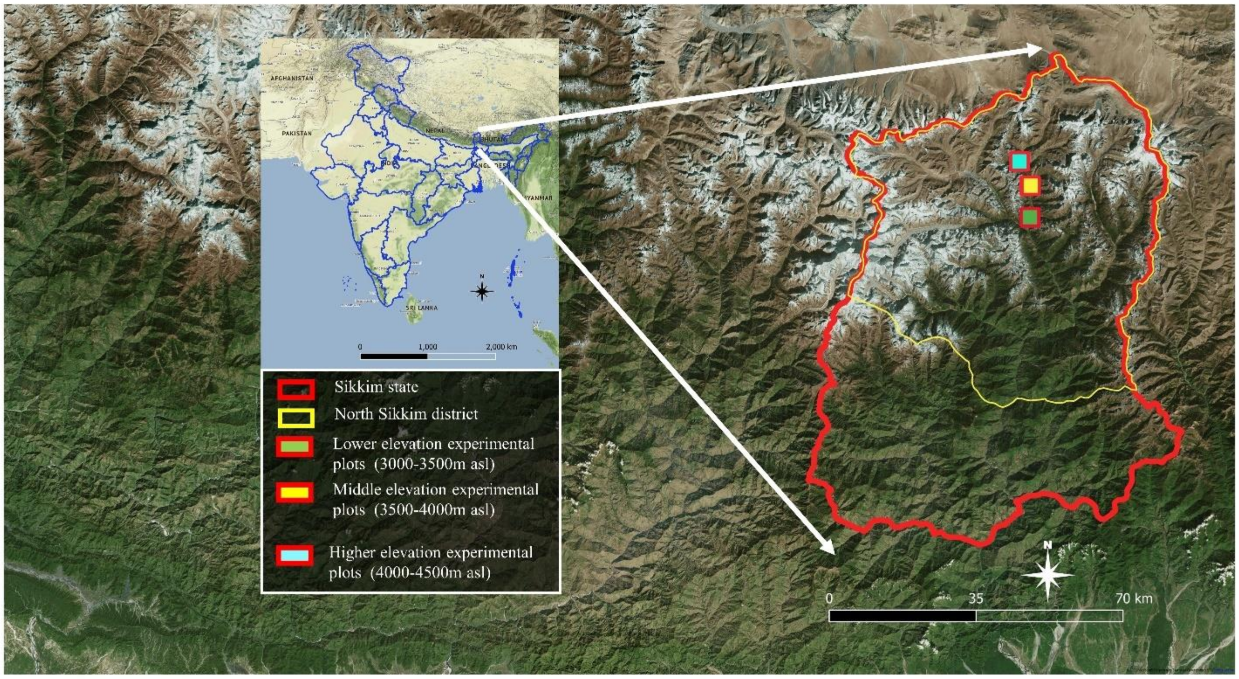

Figure 1: Map of study site with experimental plots.

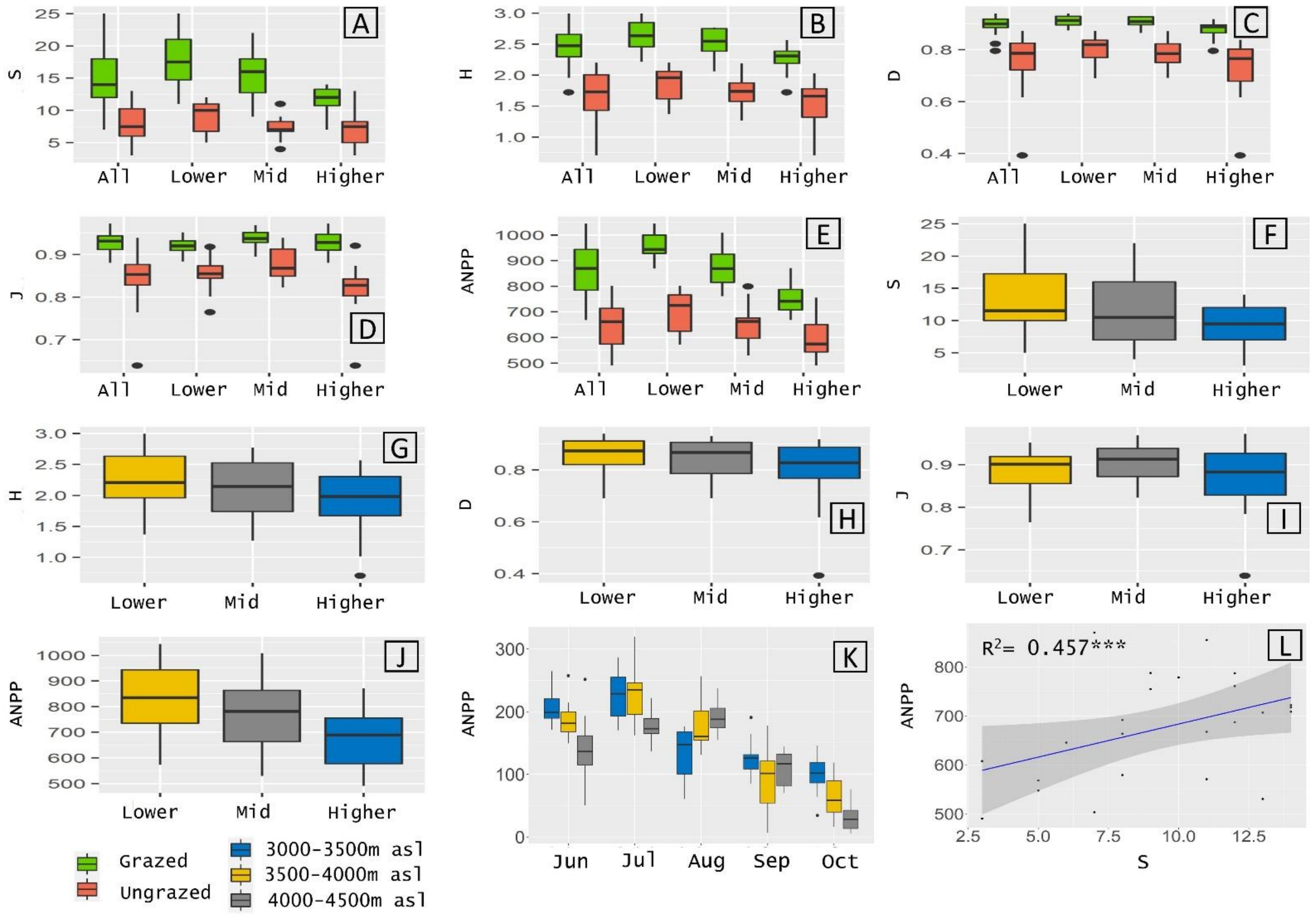

S= Species richness; H= Shannon index; D=Simpsons diversity index; J= Pielou's evenness; ANPP= Above ground net primary productivity.

Figure 2: A-J Boxplots of diversity indices and above ground net primary productivity (ANPP) for 153 species sampled from 72 plots. A-E diversity indices and ANPP in grazed vs ungrazed plot in all elevations combined (All) and in each elevation zone (lower, middle and higher). F-J diversity indices and ANPP in each elevation zone for all species. K -ANPP in each elevation zone per month (June to October). L -Linear relationship between species richness (quadrate scale) and ANPP in lower, middle and higher elevation zone quadrates. R^2 values indicated. *** represents statistical significance ($p<0.01$).

Table 1: Diversity indices and above ground net primary productivity (average per quadrate ±standard deviation) for plots in grazed and ungrazed areas in the three elevation zones and all elevations combined. P-values (represented by asterixis) indicate difference between grazed and ungrazed areas based on Welch's two sample t-test .

Elevation Zone	Quadrates	S	H	D	J	ANPP
All elevations combined	All	11.39 ±5.01	2.08 ±0.5	0.83 ±0.09	0.89 ±0.06	756.46 ± 143.33
	Grazed (G)	14.92 ±4.3	2.47 ±0.28	0.9 ±0.03	0.93 ±0.02	861 ±105.63
	Ungrazed (U)	7.86 ±2.61	1.70 ±0.35	0.77 ±0.09	0.84 ±0.05	651.92 ±89.65
	Difference (G-U)	**7.06*** **	**0.77*** **	**0.13*** **	**0.08*** **	**209.08*** **
Lower elevations	All	13.38 ±5.71	2.23 ±0.49	0.85 ±0.07	0.89 ±0.05	829.77 ±144.73
	Grazed (G)	17.83 ±4.3	2.63 ±0.26	0.91 ±0.02	0.92 ±0.02	955.27 ±52.24
	Ungrazed (U)	8.92 ±2.5	1.84 ±0.3	0.80 ±0.58	0.86 ±0.04	704.26 ± 81.87
	Difference (G-U)	**8.91*** **	**0.79*** **	**0.11*** **	**0.06*** **	**251.01*** **
Middle elevations	All	11.42 ±5.01	2.13 ±0.48	0.85 ±0.07	0.90 ±0.04	764.68 ±135.45
	Grazed (G)	15.33 ±3.8	2.53 ±0.24	0.90 ±0.02	0.94 ±0.03	873.76 ±79.28
	Ungrazed (U)	7.50 ±2.11	1.73 ±0.28	0.79 ±0.06	0.88 ±0.04	655.60 ±78.18
	Difference (G-U)	**7.83*** **	**0.8*** **	**0.11*** **	**0.06*** **	**218.16*** **
Higher elevations	All	9.38 ±3.41	1.90 ±0.49	0.80 ±0.12	0.87 ±0.08	674.93 ±107.29
	Grazed (G)	11.58 ±2.15	2.26 ±0.23	0.88 ±0.04	0.93 ±0.03	753.96 ±64.13
	Ungrazed (U)	7.17 ±3.01	1.54 ±0.41	0.72 ±0.13	0.82 ±0.07	595.90 ±79.56
	Difference (G-U)	**4.41*** **	**0.72*** **	**0.16*** **	**0.11*** **	**158.06*** **

S= Species richness; H= Shannon index; D=Simpsons diversity index; J= Pielou's evenness; ANPP= Above ground net primary productivity. P values- <0.001 "***" <0.01 '**' <0.05 '*'.

Table 2: F values from the analysis of variance for effects of grazing and elevation and their interactive effect on plant diversity and productivity.

		Grazing Treatment (T)				Elevation (E)	T X E
		All elevation zones	Lower	Mid	Higher		
		F	F	F	F	F	F
Diversity	S	70.55***	38.5***	39***	17.1	4.159*	24.15***
	H	105***	48.56***	55.16***	28.19	3.018	28.28***
	D	65.82***	38.07***	49.68***	17.01	2.687	17.29***
	J	59.03***	22.69***	20.69***	27.06	2.249	16.65***
Productivity	ANPP	81.99***	80.17***	46.07***	28.71***	8.566***	41.04***

P values- <0.001 "***" <0.01 '**' <0.05 '*'

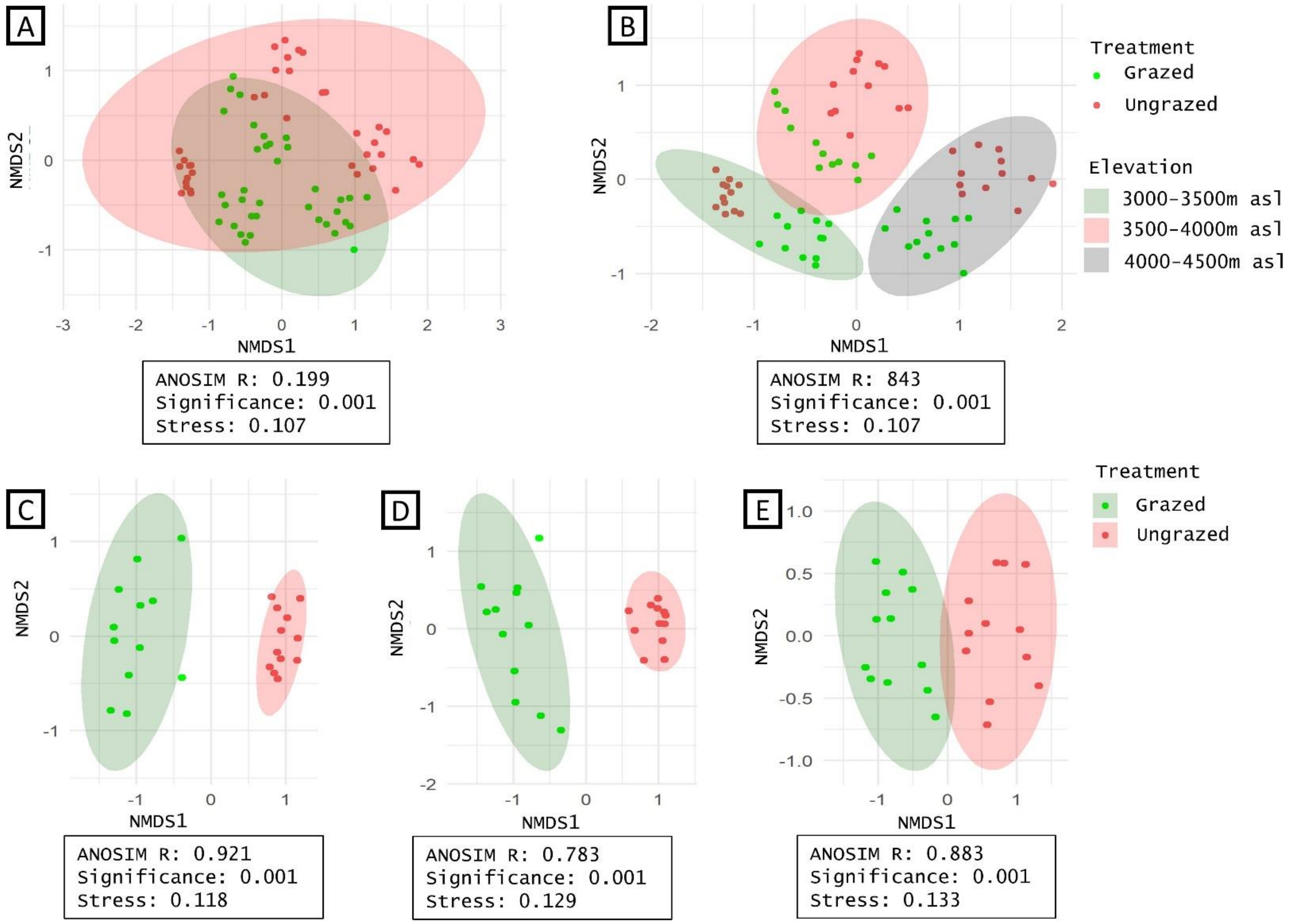

Figure 3: Non-metric multidimensional scaling (NMDS) based on Bray Curtis similarity matrix of plant communities. Analysis of similarity (ANOSIM) statistic R and its significance are indicated in boxes. Groups are represented by ellipses (95% CI around centroids). NMDS ordination of 153 species grouped according to grazing treatment (A) and elevation gradient (B); NMDS ordination of species in lower (C), middle (D), and higher elevation (E) zones respectively grouped according to grazing treatment.
