# Supplementary material for: Pastoralism in the highest peaks: Role of the traditional grazing systems in maintaining biodiversity and ecosystem function in the alpine Himalaya
Source: PLoS One. 2021 Jan 7;16(1):e0245221. doi: 10.1371/journal.pone.0245221 (PMC7790420; doi:10.1371/journal.pone.0245221)
Supplement: Attachment — Submitted filename: PONE-D-20-24843_R1_Editor Comments.pdf [file pone.0245221.s007.pdf]

PLOS ONE

Pastoralism in the highest peaks: Role of the traditional grazing systems in maintaining biodiversity and ecosystem function in the alpine Himalaya.

--Manuscript Draft--

**Manuscript Number:**	PONE-D-20-24843R1
**Article Type:**	Research Article
**Full Title:**	Pastoralism in the highest peaks: Role of the traditional grazing systems in maintaining biodiversity and ecosystem function in the alpine Himalaya.
**Short Title:**	Role of the traditional grazing systems in maintaining biodiversity and ecosystem function in alpine Himalaya.
**Corresponding Author:**	tenzing Ingty, MS<br>University of Massachusetts Boston<br>Boston, Massachusetts UNITED STATES
**Keywords:**	grazing ban policy; livestock grazing; Species diversity; Productivity; Sikkim; alpine pastures
**Abstract:**	Rangelands cover around half of the planet’s land mass and provide vital ecosystem services to over a quarter of humanity. The Himalayan rangelands, part of a global biodiversity hotspot is among the most threatened regions in the world. In rangelands of many developing nations policies banning grazing in protected areas is common practice. In 1998, the Indian state of Sikkim, in the Eastern Himalaya, enacted a grazing ban in response to growing anthropogenic pressure in pastures and forests that was presumably leading to degradation of biodiversity. Studies from the region demonstrate the grazing ban has had some beneficial results in the form of increased carbon stocks and regeneration of some species of conservation value but the ban also resulted in negative outcomes such as reduced household incomes, increase in monocultures in lowlands, decreased manure production in a state that exclusively practices organic farming, spread of gregarious species, and a perceived increase in human wildlife conflict. This paper explores the impact of the traditional pastoral system on high elevation plant species in Lachen valley, one of the few regions of Sikkim where the grazing ban was not implemented. Experimental plots were laid in along an elevation gradient in grazed and ungrazed areas. Ungrazed areas are part of pastures that have been fenced off (preventing grazing) for over a decade and used by the locals for hay formation. I quantified plant species diversity (Species richness, Shannon index, Simpson diversity index, and Pielou evenness index) and ecosystem function (above ground net primary productivity ANPP). The difference method using movable exlosure cages was used in grazing areas to account for plant ANPP eaten and regrowth between grazing periods). The results demonstrate that grazing significantly contributes to greater plant species diversity (Species richness, Shannon index, Simpson diversity index, and Pielou evenness index) and ecosystem function (using above ground net primary productivity as an indicator). The multidimensional scaling and ANOSIM (Analysis of Similarities) pointed to significant differences in plant species assemblages in grazed and ungrazed areas($P<0.001$). Further, ecosystem function is controlled by grazing, rainfall and elevation. Thus, the traditional transhumant pastoral system may enhance biodiversity and ecosystem function. I argue that a complete restriction of open grazing meet neither conservation nor socioeconomic goals. Evidence based policies are required to conserve the rich and vulnerable biodiversity of the region.
**Order of Authors:**	tenzing Ingty, MS
**Opposed Reviewers:**	
**Response to Reviewers:**	1.Editors comment:<br>Check the spelling<br>Response<br>Spelling of Rhododendron barbatum corrected<br>2.Editors comment:<br>repetition<br>Response

Powered by Editorial Manager® and ProduXion Manager® from Aries Systems Corporation

	Corrected throughout the paper<br>3.Editors comment:<br>Ecosystem function?<br>In the paper I use above ground net primary productivity (ANPP) an integrative measure of ecosystem function. This is a well-established practice where ANPP is used as a proxy for ecosystem function.<br>I had mentioned it in the abstract and have added it to the 2 broad questions in the Introduction section with citations.<br><br>4.Editors comment:<br>This part is more appropriate for conclusion rather than Introduction. Place it appropriately<br>Response<br>I have made the edit and placed it in the conclusion section<br>5.Editors comment:<br>Does the notification says so? It is better if you cite the notification stating the relaxation on grazing ban given to Lachen. Can you mention the rationale why Lachen and Lachung were excluded?<br>Response<br>I have added citations for two papers discussing grazing being allowed in selected areas of KNP and one citation for grazing in Lachen and Lachung.<br>I have not seen the exact notification and do not know why Lachen and Lachung have been excluded.<br>6.Editors comment:<br>Do we have more recent data?<br><br>Response<br>This is the most recent I found. I am assuming the livestock census like the general census is published every decade.<br>7.Editors comment:<br>But these areas also have effect on species diversity due to hay extraction.<br><br>Response<br>Yes. That is why I explicitly define what I mean by ungrazed areas. These are “areas with a history of no grazing for ateast 10 years with permanent fences around them. These areas are used by the locals to make hay.”<br><br>8.Editors comment:<br>Remove the word elevation and retain lower zone, middle zone and upper zone. Follow it throughout the text<br>Response<br>Changes made throughout the manuscript.<br>9.Editors comment:<br>How big were the blocks? Were they large enough to have sufficient distance between the grazed and ungrazed areas?<br>I have made changes to the Vegetation sampling subsection to clarify the experimental design. There were six 30mX30m plots. This included 3 in grazed areas (one for each elevation zone) and 3 in ungrazed areas (one for each elevation zone). In each plot twelve 1mX1m quadrats were laid to quantify diversity. Thus, for each elevation zone I had 24 quadrats (12 in grazed and 12 in ungrazed) with a total of 72 quadrats in all elevations and treatments where I collected data for species diversity. This is explained in the edited lines as<br>“I used a randomized block design with two treatments (grazing and ungrazed) and three replicates (lower zone =3000-3500m; middle zone =3500-400m; and higher zone= 4000-4500m) making a total of six 30mX 30m plots (2 treatments X 3 replicates)”<br>I made further corrections in the subsection- Impacts of grazing on species diversity and above ground net primary productivity (ANPP)<br>“Diversity: In each 30mX30 m plot 12 quadrats (1mX1 m) were randomly laid. We had 36 quadrates in grazed areas (12X 3elevation zones) and 36 in ungrazed areas. Thus, a total of 72 quadrats were laid (24 in each zone/ replicate). Data on species composition and percentage cover was collected from each quadrat for herb species only (forbs and graminoids).”<br>10.Editors comment:

Powered by Editorial Manager® and ProduXion Manager® from Aries Systems Corporation

Within this small area of 30m x 30 m, how it is possible to have grazed and ungrazed areas. It is not convincing because grazing animals are not restricted within 15 m (roughly as per your design) are for grazing

Response
Please see response comment above. The grazed areas were infact much larger than 30mX30m but laid our 12 plots within that plot (30mX30m) only.
11.Editors comment:
You estimated only herbs or woody vegetation were also included in the quadrat? If woody vegetation were included, your quadrat size is too small and if they are not included, how did you avoid them because in pastures also there are few woody species sparsely grown?

Response
Only herbs (forbs and graminoids) species were sampled in each quadrat. I have added this point in the subsection- Impacts of grazing on species diversity and above ground net primary productivity (ANPP). The grazing pastures we sampled (Phemkaru, Yathang and Chopta valley) had a negligible number of woody vegetation and none fell in our quadrats. In the ungrazed plots (fenced areas) there were few woody vegetation and only along the edges which we anyway avoided to avoid for edge effect.
12.Editors comment:
How did you do it in the ungrazed area?
Response
In the ungrazed areas ANPP was collected at peak standing crop once a year. I have edited the last line in the subsection Impacts of grazing on species diversity and above ground net primary productivity (ANPP) to
"In ungrazed areas twelve 1mX1m quadrats were laid and biomass was collected at peak standing crop (annually)."
13.Editors comment:
Was there a facility to do this in Lachen??
Response
No, there was no facility to oven dry in Lachen. The biomass collected was stored with blotting paper and was sent occasionally (about every 1 week to Gangtok where we had an oven to dry it).

14.Editors comment:
Please see the comments of Reviewer 3 and modify the analysis based on the comments
Response
I have conducted the analysis suggested by reviewer 3. I used the DER algorithm comparing the differences between assemblages in in terms of four components of diversity (1) rarity (Leroy's rarity index), (2) heterogeneity of the grassland community(Menhinick index), (3)evenness (McIntosh index)and (4) taxonomic distinctness (Dstar). I appreciate reviewer 3's suggestion and feel the new analysis greatly enhances the paper. I also used the Kruskal-Wallis test to test the difference between grazing treatments and between different elevations zones for diversity indices and ANPP (table1). Table 1. needs no changes since the results (significance categories) are the same. Table 2 has been omitted since the section on ANOVA has been deleted.

15.Editors comment:
Mention the packages used
Response
I have mentioned it at the end of the data analysis subsection. All analyses were performed on R. The NMDS and ANOSIM analysis were performed using the vegan package in R. I corrected my mistake- vegan is a package in R not a function.
I have also mentioned the package EcoIndR for the DER algorithm.

16.Editors comment:
Provide the list of plant species observed during sampling as supplementary material
Response
Added.
17.Editors comment:
Do not start sentence with numbers

Powered by Editorial Manager® and ProduXion Manager® from Aries Systems Corporation

Response
Changes made.
18.Editors comment:
In my viewpoint this analysis does not have any meaning. Also see the comments of Reviewer 3.
Response
Please see response to comment 14. In addition to this comparing diversity indices in grazed versus ungrazed plots is a well-established practice as is evidenced from several published papers for many years and I have compared them using a non-parametric Kruskal-Wallis test as suggested by reviewer 3. The results were the same (significance categories) and table one needs no changes.

19.Editors comment:
This analysis is not clear. Did you compare the composition among among quadrates? If so, what is the relevance to the objective of this study?
Response
Yes, the Analysis of similarity (ANOSIM) was used to check the degree of dissimilarity of species compositions. As a stand alone the results of the analysis are very interesting but in conjunction with the analysis on species richness, other diversity indices and ANPP, it does give a clearer idea of the role of grazing in diversity and ecosystem function. While without the ANOSIM the results show higher diversity and ANPP in grazed areas, the ANOSIM indicates grazing mediated difference in species assemblages/compositions may be driving these differences.

20.Editors comment:
This is an amazing finding. Hope you have highlighted the reason in the discussion section
Response
Please refer to response to comment 28

21.Editors comment:
Fig.
Number below ten are written in words
Response
Changes made throughout the paper
22.Editors comment:
plant diversity??
Response
Change made

23.Editors comment:
Use elevation uniformly throughout the text. Altitude and elevation are represented in different ways
Response
Changes made

24.Editors comment:
This sentence contradicts with your previous sentence
Response
I can see how the words “less palatable” can be confusing in this point. The species are less palatable but not unpalatable. They are still eaten by livestock unlike the species in the ungrazed plots and the PD values of these species are lower in ungrazed plots. The point I am making is small seeded species that studies show have higher survival during endozoochory have higher PD vlues in grazed versus ungrazed plots. I am not comparing it within grazed plots.
I have removed the words “less palatable” to remove any confusion. It can be reinserted if you feel otherwise.
25.Editors comment:
This paragraph looks odd here and away from the objectives of the study
Response
The first and second objective both look at the effects of grazing on plant diversity and productivity. This paragraph primarily discusses ANPP. Not acknowledging the role of rainfall in ANPP trends would suggest grazing is the single most important factor and

presents an incomplete picture hence this paragraph is important for the discussion.

26.Editors comment:
But is is nowhere shown in the result section
Response
Thank you for pointing this out. I have a reference to “Fig 2K” in this section. This paragraph discusses Fig 2K and shows how ANPP is different in different elevation zones and follows the general rainfall patterns. The results section does refer to figure 2K stating that the ANPP is highest in July (in lower, middle, zones and all combined) and August (in higher zone).
While rainfall data is not shown as it was not available for the study site. In the next sentences we do discuss the rainfall pattern for the region.

27.Editors comment:
…the Dokpas moving down to the higher elevation zones ..
Response
Sentence edited as below to clarify
“….Dokpas moving down to the lower elevations (study design’s higher zones) around mid-July”
28.Editors comment:

I did not see any explanation on why the species composition similarity was very low between grazed and ungrazed areas. It is an interesting findings but need through discussion.
Response
I agree that it is a very interesting result. I do not have an in-depth explanation although the three major points in the discussion, subsection- Impact of grazing on species diversity and ANPP touch upon this finding. The second and third point especially puts forth an argument for this. “grazing results in the reduction in abundance of dominant species by selectively removing palatable species” and “grazing can increase spatial heterogeneity thus increasing habitat diversity”. Higher pressure to reduce dominant species and higher habitat diversity in grazed areas would facilitate species unique to grazed areas. It is also understandable that a plot where no grazing has occurred for over a decade will develop different species compositions.
Showing differences in species composition is not the objective of the study but the result greatly enhances the discussion on grazing impact on diversity and productivity the primary objective of the study.

29.Editors comment:
How? “This not only ended trade with Tibet, but also led to a loss of vast areas of pastures in TAR.”
Response
I have added the word “access” to read better.
“This not only ended trade with Tibet, but also led to a loss of access to vast areas of pastures in TAR”
Here, I refer to the international border being closed after the war thus the local communities lost access to vast pastures across the border in Tibetan Autonomous region that they used before the border was closed.
30.Editors comment:
What could improve the dairy farming and pastoralism in the region? Highlight some points here
Response
I do not think a step towards improving dairy farming should be taken without sufficient knowledge on the impact that may have. In a region with very dynamic social and economic structures in addition to relatively new pressures due to the burgeoning tourism industry, there are numerous unintended consequences a top down approach may have. Will improved dairy farming incentivize the present and future generations of Dokpas from moving away from nomadic pastoralism? Will a financially successful dairy farming design prevent elite capture and deteriorate the social capital in the present cohorts? These are questions that need to be addressed before investing in improved dairy farming techniques.
To protect pastoralism, I have 2 points (that are an urgent need) discussed in the conclusion, reiterated below. In addition to this market access and reinstating pride in a traditional livelihood that is often looked at as a last resort by the present generation of

Powered by Editorial Manager® and ProduXion Manager® from Aries Systems Corporation

local communities would go a long way.
1.“payment for ecosystem services schemes for the services provided by pastoralists in maintaining biodiversity and ecosystem function”. This has been practiced in other parts of the world notably in Switzerland.
2.“An efficient compensation scheme for human wildlife conflict that is affecting marginalized communities needs to be developed.”

31.Editors comment:
Highlight – in citation
Response
Citation format has been corrected

Reviewer #1: comment

Abstract:
In the abstract you mentioned “This paper explores the impact of the traditional pastoral system on high elevation plant species in Lachen valley, one of the few regions of Sikkim where the grazing ban was not implemented.” Clearly mention in the abstract which were the current grazing stands and which were the ungrazed forests or plots or treatments you selected in your study. You have not mentioned this and thus following your previous sentence of the study site in the Lachen Valley your sentence speaking about your result does not match in the subsequent sentences.

Response
I have added 2 lines in the abstract clearing this point. The second line clarifies “Ungrazed areas are part of pastures that have been fenced off (preventing grazing) for over a decade and used by the locals for hay formation.”

Reviewer #1: comment
Please give a clear recommendation as to what the policy makers should do. Do they need to revisit their policy….
Response
Conclusion-point 3 explicitly states that the grazing ban policy needs to be revisited. “The present grazing ban policy needs to be revisited with a more holistic approach”
Reviewer #1: comment
….develop strategic short-term and long term strategic action plans through government notification and initiate regulated grazing allowing the herders to graze livestock??

Response
As discussed in the conclusion (point 1) “Experimental studies reintroducing grazing in small areas in regions where the grazing ban was enacted need to be conducted. Studies with the aim to understand the role of different livestock species and different grazing intensities on biodiversity structure and function need to be conducted.”
Further studies built on our research would be needed to gather more data on different grazing intensities and different livestock species before initiating “regulated grazing allowing the herders to graze livestock”
Reviewer #1: comment
Introduction

The last paragraph should go to the discussion section.
Response
Thank you for pointing this out. It has been moved to the conclusion section on the recommendation of the editor.

Powered by Editorial Manager® and ProduXion Manager® from Aries Systems Corporation

	Reviewer #1: comment<br><br>1. The author can develop a GIS based Hyposometric map with the location of the sampled elevations map along the elevation 3000-4500 m with drainage systems, name of the study sites etc. in a DEM.<br>Response<br>Thank you very much for the recommendation. Figure 1 has been redone using a Digital Elevation Model (DEM) derived from ASTER data<br>Reviewer #1: comment<br>2. How did you differentiate the grazing with the domestic ungulates and the wild ungulates ?<br>Response<br>As stated in the sub-section under Vegetation sampling. "I studied the combined effects of different grazing species" (wild and domestic).<br>"It was difficult to separate the effects of different grazers since there was much overlap in the grazing areas shared by the different livestock and wild species. Cows, bulls, goats, horses and a few yak shared the same pastures in the lower elevation zone (3000-3500 m asl). Sheep, yak, cows, bulls, goats, and horses shared the middle elevation zones (3500-4000 m asl) and yak, sheep and a few horses grazed in the higher elevation zone (4000-4500 m asl). Thus, I studied the combined effects of different grazing species."<br>Reviewer #1: comment<br>3. When and where was the fencing laid, was that laid in all the study sites ?<br>Response<br>The fenced (ungrazed areas) were laid in all 3 elevation zones. Within Lachen valley there are several areas fenced off by the locals (for atleast 10 years before the study was conducted). These areas are used by the locals to make hay. The herbs are left to grow in these areas throughout the year and removed at the end of the growing season<br>"Local pastoralists and village representatives were consulted to select grazed and ungrazed areas" One fenced area was in Phemkaru (lower zone), Yathang (middle zone) and Chopta (higher zone). The name of places is mentioned in the edited map (figure 1). The details of fencing is described in the MS in the third sentence of the section on Methods-vegetation sampling<br>"The ungrazed areas were defined as areas with a history of no grazing for ateast 10 years with permanent fences around them. These areas are used by the locals to make hay. The herbs are left to grow in these areas throughout the year and removed at the end of the growing season."<br>Reviewer #1: comment<br>4. What was the study period of this research ?<br>Response<br>The study was initiated in 2010 but due to the earthquake of 2011 data collection was disrupted. Preliminary data was recollected in 2012 August- October. Finalized data was collected over a three-year period starting from the growing season of 2013 to the end of the growing season in 2015. I have added this to the materials and methods section under the sub-section Vegetation sampling.<br>Reviewer #1: comment<br>5. Which were the livestock, domestic as well as wild ungulates, season of grazing, and the season of your study, is not clear.<br>Response<br>Domestic livestock is described in detail in the vegetation sampling section (MS quoted below). We did not monitor wild ungulate population. As stated in this section "I studied the combined effects of different grazing species" this include the domestic livestock mentioned as well as wild ungulates.<br>"Cows, bulls, goats, horses and a few yak shared the same pastures in the lower elevation zone (3000-3500 m asl). Sheep, yak, cows, bulls, goats, and horses shared the middle elevation zones (3500-4000 m asl) and yak, sheep and a few horses grazed in the higher elevation zone (4000-4500 m asl). Thus, I studied the combined effects of different grazing species. "<br>Reviewer #1: comment<br>6. While the grazed stands were found to be with higher species richness in all the elevation study sites, please give a list of common palatable and unpalatable species found in both the grazed and unglazed sites. This will help policy maker to understand the conservation of important, or the threatened species.

Powered by Editorial Manager® and ProduXion Manager® from Aries Systems Corporation

Response
Please refer to reply to comment no. 12
Reviewer #1: comment
7. Please give number of Dokpa households under Lachen Dzumsa (Gurudongmar-Tsholhamu, and Muguthang), as you have said the population is declining. You have mentioned in the last paragraph before the conclusion section that over a dozen Dokpas are there (???).
Response
The number of households has been revised to an older (2002) source with a citation. The number mentioned in the previous version of the manuscript was a result of conversations with Dokpas who mentioned that there were fewer than a dozen families left actively practicing nomadic pastoralism.
Reviewer #1: comment
8. It is important to note the stocking density to identify the impact of grazing, please justify your result by showing the stocking density in the study elevations. Did you come across any location of overstocking ??
Response
While I agree with the reviewer that calculating stocking density would add value to the study we collected data on type of livestock in plots and not number of individual livestock, and wildlife species were also unaccounted for. If we estimated of stocking densities based on government census data (available for the whole valley and not blocks) it would be inaccurate and misleading to policy makers.
Moreover as Laca in Rangeland Ecol Manage.(2009) point out productivity will be affected by grazing method, bite rates and the defoliation process in addition to stocking density. My study focuses on the vegetation response to traditional grazing system comparing grazed areas versus ungrazed (traditionally fenced areas)

Reviewer #1: comment

9. Which species were dominant in the three study elevations, as you said the dominant species were reduced due to grazing. Were they all palatable ?
Response
Graminoid species were most dominant with Kobresia nepalensis (Cyperaceae) being the most common species in all 3 elevations. Yes, the graminoid species were all palatable.
I have made no changes to the manuscript as this information is present in paragraph 4 of the discussion and paragraph three of the results section.
Reviewer #1: comment

10. Any information on Pray-Predator relationship, impact of grazing on wildlife, livestock depredation etc. will add value to the MS.
Response
I completely agree, unfortunately this was outside the objective and scope of the study and we did not collect information on wildlife.

Reviewer #1: comment
11. There is a notion that the Human-Wildlife conflict has increased after ban on grazing, please justify if any such information could be generated during the course of this study.
Response
I did not come across such information since the study was conducted in areas where grazing was not banned. I have cited papers from parts of Sikkim that have seen a perceived increase in human wildlife conflict after the grazing ban (reference 38[Bhagwat et al.2012], 40 [Tambe et al. 2005], 88 [Gupta et al. 2020])
Reviewer #1: comment

12. What was the species composition in the grazed plots, how did you identify the species as the palatable species are already grazed by animals?
Response
Species were identified as palatable based on literature reviews, consultations with locals and observations. Species were deemed palatable if they met all 3 above criteria.
I sampled herbs (forbs and graminoid species only). (This has been added in the methods section). Based on the 3 criteria above we were able to confirm that

graminoid species were indeed palatable. Especially cyperaceae and poaceae. Juncaceae was less palatable but was still grazed when more palatable species were eaten. (This has been added to the Discussion section- Impacts of grazing on species diversity and ANPP- paragraph 3). I did not classify other species as less palatable unless they did not meet all 3 criteria and grazers avoided these species eg. Anaphalis lactea Lentopodium leontopodioides and Morina longifolia.

Reviewer #1: comment

13. Clearly mention in your result the species composition based on their season of appearances, snowfall period
Response
Data was collected only during the growing season (mid-April to late October) and not during the snowfall period. I have added as supplementary material a table on species composition.
Reviewer #1: comment
14. It would be nice to give high resolution pictures of the stand of both grazed and un-grazed stands.

Reviewer #1: comment
15. What kind anthro0pogenic pressure were there in grazing stands other than grazing ?
Response
Although close to the road the grazing pastures had no other anthropogenic pressure and maintained solely for grazing. Occasional visits by tourists do occur resulting in trampling only but no construction.
Reviewer #1: comment
16. Also show in your result what is the species richness in the three elevations ?
Response
It is present in table 1. I have added it to the results section too.
Reviewer #1: comment
17. How did you measure ANPP in the grazed and ungrzaed plots, as the plant biomass of grazed plots were eaten up by animals ?
Response
As mentioned in the methods section I followed the difference method by McNaughton et al. (1996) (ref.51). This is a standard method that is most preferred to measure the effect of grazing on plants "since moveable exclosures account for the amount eaten and the amount that regrows between feeding bouts" (McNaughton et al. 1996). Since this is a highly cited paper there is no need to repeat the details of the method more than what is already detailed in the methods section.
Reviewer #1: comment
18. Which species do you think are dispersed by animals?
Response
This is a very interesting question. Any names here would be only conjecture and a separate study focusing on epizoochory and endozoochory would be needed to confirm. Unfortunately, this was outside the objective and scope of the study. If I would have to guess Asteraceae species that may be principally wind pollinated may be occasionally dispersed long distances by endozoochory.

Reviewer #2: General Comments:

1. Author should give special attention in the spellings of the botanical names of the plant species, grammar and punctuation

Response
Thank you. I have made the edits and corrections
Reviewer #2: comment
2. Repetition of words encountered in places which needs to be checked

Powered by Editorial Manager® and ProduXion Manager® from Aries Systems Corporation

Response
Thank you. I have made the edits and corrections

Specific Comments:

Reviewer #2: comment
1. Abstract

• Such long introduction in abstract is not required. Just restrict to one or two sentences.
• Point out the methods applied for the study in one or two sentences
Response
Thank you. Changes made.
Reviewer #2: comment
• Highlight the most significant findings in the result section in one or two sentences instead of generalizing it. For eg., The multidimensional scaling and ANOSIM (Analysis of Similarities) pointed to significant differences in plant species assemblages in grazing and ungrazed areas. Significant at what level?
Response
Thank you. Changes made.

Reviewer #2: comment

2. Introduction

• The author have mentioned in third paragraph (line 6 – 8) that "Open grazing was phased out over the next six years in all reserved forests with exception of Lachen and Lachung valley and alpine pastures in the Kangchenzonga National Park". Logically, grazing is strictly not allowed in National Parks. This statement may negatively impact the UNESCO's World Heritage Site status of the KNP. Author should avoid using such statement or should provide supporting references.
Response
I have added citations for two papers discussing grazing being allowed in selected areas of KNP.
Reviewer #2: comment

• The last paragraph in the introduction section looks more like a part of the discussion based on the present study. Hence, move the paragraph to the discussion section.
Response
I have made the edit and placed it in the conclusion section
Reviewer #2: comment

3. Materials and Methods

• Normally, above 2000 m asl elevation are considered as higher elevation. Therefore, it would not be appropriate to label the elevation above 3000 m asl as lower elevation zone, middle elevation zone or high elevation zone. Try to find some suitable terminology or replace it with lower zone , middle zone and upper zone duly mentioning the elevation range in the text as well as in the map.
Response
Thank you for the suggestion, I have made the changes
Reviewer #2: comment

• Method is vague. How is it possible to have both grazed and un-grazed area in the same plot of 30 x 30 m? Needs to be clarified. Further, the unit must be accurate. Either write 30 m x 30 m or replace it with 30 x 30 sq. m.

Response
I have made changes to the Materials and methods section under sub-section vegetation sampling
Reviewer #2: comment

• Author have mentioned the establishment of 12 movable enclosure cages to study the biomass growth. It is not clear weather all the cages were set up in the same plot at

Powered by Editorial Manager® and ProduXion Manager® from Aries Systems Corporation

a time. If not then, it is important to mention as how many cages were set up in each plot.

Response
I have made changes to the Materials and methods section under sub-section vegetation sampling
Reviewer #2: comment
• Major rewriting of the methods under the sub-heading "Impact of grazing…..", a part of the vegetation sampling section is needed to clear the confusion.
Response
Thank you. Changes made

Reviewer #2: comment

• Under the subheading 1, 2 para, line 4: The standing biomass of was……..what does it mean? please specify or correct the statement.
Response
I have changed it to "aboveground standing biomass (vegetation)"
Reviewer #2: comment

• Data analysis: Table1 & 2 is the part of the result section; hence, remove the mention of table 1 & 2 from the data analysis section.
Response
Changes made

Reviewer #2: comment

• What is the logic behind calculating Shannon Index and Simpson diversity index? As both the indexes calculates the diversity taking into account the species richness and the relative abundance of the species. It is better if the author calculates the dominance index.
Response
As suggested by reviewer #3 I compared the differences between assemblages in terms of rarity (Leroy's rarity index), heterogeneity of the grassland community(Menhinick index), evenness (McIntosh index)and taxonomic distinctness (Dstar). The four the components of diversity were combined into a single measurement using the DER_algorithm in the EcoINDR package in R. In addition to this I also calculated proportional dominance.
If permitted, I would prefer to leave both Simpson diversity and Shannon indices if not I would prefer to leave in the Shannon index. Having both indices may be useful as part of a larger meta-analysis. Recent publications (eg. Lu et al. 2017;Ecosphere) have used both indices as part of their meta-analysis. I think meta-analyses play an important role as a policy tools and my primary data would help feed into larger analyses.

Reviewer #2: comment

4. Results

Species diversity indices:

• Why the same result is depicted in table as well as the box plot (Fig 2 A-D, Table 1). Either use table or figure.
Response
I am fine with fig 2A-D being removed. Although I would prefer it being retained since the boxplots give an idea of the spread of data that the standard deviations (in the table) fail to display.
Reviewer #2: comment

• Para 1, line no.: 8 & 9. Author have mentioned that "Grazing had a significant (ANOVA p<0.001) effect all diversity indices in each elevation zone and all elevation zones combined but from table 2, it is clear that the effect of grazing is not significant for diversity indices in higher elevation. Address this mistake.
Response

Thank you for catching this. Changes have been made to table 2.

Reviewer #2: comment

• Para 1, last 3 lines. Rewrite the sentence “Unlike grazing, elevation did not have a significant effect (ANOVA p<0.05)diversity indices (fig. 2G-I, table 2) except S (fig. 2F, table 2)” as “Unlike grazing, elevation did not have a significant effect on diversity indices (Fig 2G-I) except S (p<0.05) [Fig. 2F, table 2)”.
Response
Changes made

Reviewer #2: comment

• Para 2. Line no. 2-3. The sentence is unclear whether the author is trying to compare the species composition between grazed and un-grazed treatment in different zones or amongst the replicates of grazing treatment in different zones. Needs rewriting.
Response
Thank you. I have rewritten the 2 lines
Reviewer #2: comment

• ANPP: Rewrite the sentence “The mean ANPP for all grazed quadrates (all elevation zones combined), lower and middle elevation zone grazed quadrates was highest in July (Fig 2K) and in the higher elevation zone grazed quadrates in August (Fig 2K)” as “The mean ANPP was highest in July for all grazed quadrates in lower zone, middle zone and all zones combined but it was highest in August in higher zone (Fig 2 K)”.
Response
Changes made
Reviewer #2: comment

5. Discussion

• It should focus more on discussing the result of the present study duly supported by the relevant studies in other Himalayan region. Needs major rewriting especially the first two parts.

Reviewer #3: comment

The manuscript has been written well and the research design appears to be rigorous. However, the description I believe is a little confusing. The way I understood the design is:
Elevation: Low, Middle, High
Blocks: G (grazed) and U (ungrazed)
Replicates: 3/elevation [3x3 = 9]
Plots: 30x30m, one per block [total = 9] This is confusing – was the 30x30m plot equally straddling G and U areas?
Sampling quadrats: 24 per block (12G and 12U) [24, 1x1m quadrats/elevation, and a total of 216 for the study – while implied (perhaps), this should be mentioned in the text]
If this is correct, a simple chronological statement or a schematic of the levels and

Powered by Editorial Manager® and ProduXion Manager® from Aries Systems Corporation

	arrangement will make following the experimental set-up easy for readers.<br>Response<br>I have made changes to the Materials and methods section under sub-section vegetation sampling that should clarify the study design<br><br>Reviewer #3: comment<br><br>One of my major concerns with the manuscript is the analyses carried out with the diversity values. Here the author has used a variety of diversity measures – Shannon entropy, Simpson concentration, and Pielous's evenness. Nothing is alarming about these indices by themselves, but the problem arises once these values are further analyzed, say using an ANOVA. First off, these indices are limited in range – usually between 0 and 1, for Shannon's entropy when normalized, for example; exhibiting a floor-ceiling effect and violating the assumptions of a traditional ANOVA. This often leads to wrong results and comparisons. Details of this problem may be further understood by reviewing the paper by Lou Jost in Oikos 2006 – Diversity and Entropy. There are methods suggested in this paper that makes comparing entropies more meaningful. Therefore, instead of using an ANOVA to tease apart the difference among treatments, I suggest that the author use a more comprehensive and inclusive algorithm such as the DER – for comparing species diversity. See Guisande et al. 2017. This can be done in R (package EcoIndR). These are more contemporary techniques and overcome the flaws of other traditional comparison techniques.<br><br>Please see attached an annotated content extracted pdf (lit rev not included) of the manuscript for minor edits that will help the paper read well.<br><br>Other than that, the research represented by the manuscript is timely and important in understanding ways of restoring the plant diversity of the alpine regions of the Himalaya and elsewhere, especially using adaptive management policies.<br>Response<br>Thank you very much for the suggestion. I have done the analysis using the DER algorithm comparing the differences between assemblages in in terms of rarity (Leroy's rarity index), heterogeneity of the grassland community(Menhinick index), evenness (McIntosh index)and taxonomic distinctness (Dstar). I also used the Kruskal-Wallis test in place of the t-test used earlier. I also used the Kruskal-Wallis test to test the difference between grazing treatment and between different elevations zones for diversity indices and ANPP and removed the ANOVA analysis.
**Additional Information:**	
**Question**	**Response**
**Financial Disclosure**<br><br>Enter a financial disclosure statement that describes the sources of funding for the work included in this submission. Review the submission guidelines for detailed requirements. View published research articles from *PLOS ONE* for specific examples.<br><br>This statement is required for submission and **will appear in the published article** if the submission is accepted. Please make sure it is accurate.	Field work was supported by funding from<br>1. Department of Biotechnology, Ministry of Science and Technology Government of India to Ashoka Trust for Research in Ecology and the Environment, Bangalore, India (atree.org)<br>2. ATREE Small Grants for research in Northeast India to TI<br>3. Sanofi Genzyme and Oracle Fellowship to TI<br><br>The funders had no role in study design, data collection and analysis, decision to publish, or preparation of the manuscript

Powered by Editorial Manager® and ProduXion Manager® from Aries Systems Corporation

**Unfunded studies**<br>Enter: *The author(s) received no specific funding for this work.*<br><br>**Funded studies**<br>Enter a statement with the following details:<br>• Initials of the authors who received each award<br>• Grant numbers awarded to each author<br>• The full name of each funder<br>• URL of each funder website<br>• Did the sponsors or funders play any role in the study design, data collection and analysis, decision to publish, or preparation of the manuscript?<br>• **NO** - Include this sentence at the end of your statement: *The funders had no role in study design, data collection and analysis, decision to publish, or preparation of the manuscript.*<br>• **YES** - Specify the role(s) played.<br><br>* typeset	
**Competing Interests**<br><br>Use the instructions below to enter a competing interest statement for this submission. On behalf of all authors, disclose any competing interests that could be perceived to bias this work—acknowledging all financial support and any other relevant financial or non-financial competing interests.<br><br>This statement **will appear in the published article** if the submission is accepted. Please make sure it is accurate. View published research articles from *PLOS ONE* for specific examples.	No competing interests. This is a single author publicaiton.

Powered by Editorial Manager® and ProduXion Manager® from Aries Systems Corporation

**NO authors have competing interests**<br><br>Enter: *The authors have declared that no competing interests exist.*<br><br>**Authors with competing interests**<br><br>Enter competing interest details beginning with this statement:<br><br>*I have read the journal's policy and the authors of this manuscript have the following competing interests: [insert competing interests here]*<br><br>* typeset	
**Ethics Statement**<br><br>Enter an ethics statement for this submission. This statement is required if the study involved:<br><br>• Human participants<br>• Human specimens or tissue<br>• Vertebrate animals or cephalopods<br>• Vertebrate embryos or tissues<br>• Field research<br><br>Write "N/A" if the submission does not require an ethics statement.<br><br>General guidance is provided below. Consult the submission guidelines for detailed instructions. **Make sure that all information entered here is included in the Methods section of the manuscript.**	Field Research.<br>Permit number: 03592280402<br>Department of Forest, Environment & Wildlife Management, Government of Sikkim, India.

Powered by Editorial Manager® and ProduXion Manager® from Aries Systems Corporation

**Format for specific study types**<br><br>**Human Subject Research (involving human participants and/or tissue)**<br>• Give the name of the institutional review board or ethics committee that approved the study<br>• Include the approval number and/or a statement indicating approval of this research<br>• Indicate the form of consent obtained (written/oral) or the reason that consent was not obtained (e.g. the data were analyzed anonymously)<br><br>**Animal Research (involving vertebrate animals, embryos or tissues)**<br>• Provide the name of the Institutional Animal Care and Use Committee (IACUC) or other relevant ethics board that reviewed the study protocol, and indicate whether they approved this research or granted a formal waiver of ethical approval<br>• Include an approval number if one was obtained<br>• If the study involved *non-human primates*, add *additional details* about animal welfare and steps taken to ameliorate suffering<br>• If anesthesia, euthanasia, or any kind of animal sacrifice is part of the study, include briefly which substances and/or methods were applied<br><br>**Field Research**<br><br>Include the following details if this study involves the collection of plant, animal, or other materials from a natural setting:<br>• Field permit number<br>• Name of the institution or relevant body that granted permission	
**Data Availability**<br><br>Authors are required to make all data underlying the findings described fully available, without restriction, and from the time of publication. PLOS allows rare exceptions to address legal and ethical concerns. See the PLOS Data Policy and FAQ for detailed information.	Yes - all data are fully available without restriction

A Data Availability Statement describing where the data can be found is required at submission. Your answers to this question constitute the Data Availability Statement and **will be published in the article**, if accepted.<br><br>**Important:** Stating 'data available on request from the author' is not sufficient. If your data are only available upon request, select 'No' for the first question and explain your exceptional situation in the text box.<br><br>Do the authors confirm that all data underlying the findings described in their manuscript are fully available without restriction?	
**Describe where the data may be found in full sentences. If you are copying our sample text, replace any instances of XXX with the appropriate details.**<br><br>• If the data are **held or will be held in a public repository**, include URLs, accession numbers or DOIs. If this information will only be available after acceptance, indicate this by ticking the box below. For example: *All XXX files are available from the XXX database (accession number(s) XXX, XXX.).*<br>• If the data are all contained **within the manuscript and/or Supporting Information files**, enter the following: *All relevant data are within the manuscript and its Supporting Information files.*<br>• If neither of these applies but you are able to provide **details of access elsewhere**, with or without limitations, please do so. For example:<br><br>*Data cannot be shared publicly because of [XXX]. Data are available from the XXX Institutional Data Access / Ethics Committee (contact via XXX) for researchers who meet the criteria for access to confidential data.*<br><br>*The data underlying the results presented in the study are available from (include the name of the third party*	All relevant data are within the manuscript.

and contact information or URL).<br>• This text is appropriate if the data are owned by a third party and authors do not have permission to share the data.<br><br>* typeset	
Additional data availability information:	

Dear Dr. Acharya,

I would like to thank you and the three reviewers for your comments and suggestions. I found them very useful and as a result the revised version represents a considerable improvement over the first version.

I have addressed all the comments from you and the reviewers and made edits where suggested (please see the document Response to Reviewers). I would like to highlight three major edits made in this version.

1. Figure 1. The map of the study site has been revised using a Digital Elevation Model (DEM) derived from ASTER data. The appropriate citation is also given in the manuscript.
2. The study design has been rewritten to clarify the queries raised.
3. The analysis using ANOVA has been removed and I conducted the analysis suggested by reviewer 3. I used the DER algorithm comparing the differences between assemblages in in terms of four components of diversity (1) rarity (Leroy's rarity index), (2) heterogeneity of the grassland community(Menhinick index), (3)evenness (McIntosh index)and (4) taxonomic distinctness (Dstar). Table 2 (F values from the ANOVA) has been deleted.
4. As suggested by reviewer 3 I compared diversity indices and ANPP in different grazing treatments and different elevation zones using a non-parametric Kruskal-Wallis test. The results (significance categories) were the same as the previous analysis and table one needs no changes.
5. As supporting information, I have added the list of species detailing whether found in grazed/ungrazed or both treatments.

I received graduate student scholarship from Sanofi Genzyme and Oracle fellowship (a commercial source). This does not alter our adherence to PLOS ONE policies on sharing data and materials.

In closing I am hoping you will accept the revised version since the reviewers' comments were largely positive, and I have made their suggested changes.

Kind Regards,

Tenzing Ingty

Manuscript Click here to access/download;Manuscript;Manuscript.docx

Pastoralism in the highest peaks: Role of the traditional grazing systems in maintaining biodiversity and ecosystem function in the alpine Himalaya.

Tenzing Ingty[1]
[1]*Department of Biology, University of Massachusetts, Boston, MA*

tenzingingty@gmail.com

Abstract

Rangelands cover around half of the planet's land mass and provide vital ecosystem services to over a quarter of humanity. The Himalayan rangelands, part of a global biodiversity hotspot is among the most threatened regions in the world. In rangelands of many developing nations policies banning grazing in protected areas is common practice. In 1998, the Indian state of Sikkim, in the Eastern Himalaya, enacted a grazing ban in response to growing anthropogenic pressure in pastures and forests that was presumably leading to degradation of biodiversity. Studies from the region demonstrate the grazing ban has had some beneficial results in the form of increased carbon stocks and regeneration of some species of conservation value but the ban also resulted in negative outcomes such as reduced household incomes, increase in monocultures in lowlands, decreased manure production in a state that exclusively practices organic farming, spread of gregarious species, and a perceived increase in human wildlife conflict. This paper explores the impact of the traditional pastoral system on high elevation plant species in Lachen valley, one of the few regions of Sikkim where the grazing ban was not implemented. Experimental plots were laid in along an elevation gradient in grazed and ungrazed areas. Ungrazed areas are part of pastures that have been fenced off (preventing grazing) for over a decade and used by the locals for hay formation. I quantified plant species diversity (Species richness, Shannon index, Simpson diversity index, and Pielou evenness index) and ecosystem function (above ground net primary productivity ANPP). The difference method using movable exlosure cages was used in grazing areas to account for plant ANPP eaten and regrowth between grazing periods). The results demonstrate that grazing significantly contributes to greater plant species diversity (Species richness, Shannon index, Simpson diversity index, and Pielou evenness index) and ecosystem function (using above ground net primary productivity as an indicator). The multidimensional scaling and ANOSIM (Analysis of Similarities) pointed to significant differences in plant species assemblages in grazed and ungrazed areas($P<0.001$). Further, ecosystem function is controlled by grazing, rainfall and elevation. Thus, the traditional transhumant pastoral system may enhance biodiversity and ecosystem function. I argue that a complete restriction of open grazing meet neither conservation nor socioeconomic goals. Evidence based policies are required to conserve the rich and vulnerable biodiversity of the region.

Keywords

grazing ban policy, rangelands, species diversity, productivity, Himalaya, alpine pastures

Introduction

Rangelands cover approximately half of earth's land surface [1,2] and over a quarter of humanity depend on these land types for their livelihoods and numerous ecosystem services [3,4]. The ecosystem services include provisioning services like food, fiber and water, and supporting services such as carbon storage (roughly 30% of global soil carbon) among many others [5–8]. Rangelands are also recognized for their high biodiversity, including numerous endangered and endemic species, as well as habitat connectivity between protected forests [6,7,9,10]. In much of the developing world, rangelands are inhabited by pastoral communities. Pastoralism that has existed for millennia has impacted the evolution of many rangelands, from the biodiverse rich African savanna [11] to the highest elevations in the Tibetan steppe [12]. Pastoralists and the rangelands they inhabit thus form complex socio-ecological systems that have co-evolved across different geographies, biodiversity and climates [11,13]. Many pastoralists have been blamed, often wrongly, for rangeland degradation [14,15] and targeted for "*modernization*" programs such as sedenterization. While some of these programs have been beneficial [6,16–18], most have shown to have socially and ecologically disastrous impacts resulting in a decline in wildlife, decreased grassland diversity and productivity, and increased desertification [14,19,20]. Moreover, the abandonment of rangelands by pastoralists and climate change is leading to rapid shrub invasion, further increasing habitat loss [21,22]. Grazing by herbivores such as the semi domesticated reindeer has been shown to counteract the effect of warming on shrub expansion [23–26]. This suggests that grazing management is an important factor to maintain alpine areas in response to climate change driven shrub invasion.

Rangelands in the Himalaya, a global biodiversity hotspot [27], form the largest land use system and provides numerous ecosystem services to pastoral communities [28]. The Himalaya is the source of 10 of the largest rivers in Asia whose basins feed about one fifth of humanity. The alpine rangelands of the Himalaya thus play a vital role in retaining critical ecosystem services such as carbon sequestration, water storage and provisioning, maintaining biodiversity, and food security and livelihoods. Despite its importance a report on high elevation rangelands of the Himalaya [28] underline numerous threats such as invasive species, inappropriate management and development policies, overgrazing and climate change. Studies show that climate change has significantly impacted the Himalaya. These include an increase in average temperatures three times higher than the global average, altered phenology and shifts in distribution ranges of biodiversity [29–33]. Upward shifts in treelines and shrubs have led to woody ingression into alpine pastures [34–36].

The alpine rangelands of the tiny state of Sikkim in the Eastern Himalaya are among the most biodiverse regions in the world. They occupy just 0.006% of global alpine rangelands but harbor as much as 60% of the global alpine plant families and 10% (297 genera) of global alpine genera with more than twice the number of vascular plants than the Alps and Rockies [37]. In 1998 the government of Sikkim adopted a grazing exclusion policy in response to growing anthropogenic pressure on grazing lands and protected forests leading to perceived degradation. Open grazing was phased out over the next six years in all reserved forests with exception of alpine pastures in

the Kangchenzonga National Park [38,39], Lachen and Lachung valleys [40].. The forest cover of the state between 1999 to 2017 increased by about 4% from 3118 km^2 to 3379 km^2. Studies in Barsey Rhododendron Sanctuary in West Sikkim estimate that twelve years after the grazing ban was declared carbon stocks increased by about 585 thousand tonnes and the concurrent increase in regeneration of species of high conservation value such as *Rhododendron barbatum*[41,42]. At the same time, Bhagwat et al. [41] also warned of insignificant regeneration of important fodder species and other species of high conservation value such as *Quercus spp.* coupled with a spread of gregarious species such as *Arundinaria maling* and *Viburum cordifolium*. Restriction on free access to forest resources have had significant socio-economic impacts on the herders who were dependent on grazing pastures in the sanctuary. Their average net income from livestock was reduced by more than half the pre-ban period, with the source of majority of the incomes now coming from government subsidies and schemes like the National Rural Employment Guarantee Act programme (MGNREGA) for unskilled labor [41,43]. A study on the perception of sedenterized ex-herders from West Sikkim identified loss of traditional livelihoods leading to decreased income and limited land holdings as their main protestations. In addition to socio-economic impacts to herders the grazing ban has had a cascading impact on non-herders[41]. Perceptions of local villagers indicate an increase in human wildlife conflict in the form of crop raids and livestock depredation with over 85% of the households experiencing greater losses in agricultural outputs after the grazing ban [41,43].

Here, I explore the impact of the present pastoral system on high elevation plant species in Lachen valley, Sikkim. The region is of significance for two reasons. First, it falls in one of the most biodiverse regions of the world, the alpine zone of the Sikkim Himalaya [37]. Second transhumant pastoralism has been practiced in the region for centuries with the indigenous communities directly dependent on the biodiversity of their surrounding for livestock husbandry, water storage and provisioning, and the collection of medicinal, aromatic, and edible plants [38,44,45].

I address 2 broad questions:

1. What is the impact of the traditional grazing system on plant diversity and ecosystem function (using aboveground net primary productivity as an integrative measure of ecosystem function[46,47]) in the high-elevation alpine Himalaya?
2. How does grazing maintain biodiversity and ecosystem function in the region?

I first analyze the impact of grazing on diversity and productivity. I then discuss the role of grazing in maintaining high biodiversity and ecosystem function. Finally, I conclude by identifying further knowledge needs and propose recommendations for an improved grazing policy to protect the rich yet vulnerable biodiversity of the region.

Materials and methods

Study area

(Field Research. Permit number: 03592280402 Department of Forest, Environment & Wildlife Management, Government of Sikkim, India.)

The study was conducted in Lachen valley in the North district of the state of Sikkim in India. Sikkim, in the Eastern Himalayan region (Fig 1), is the second smallest (7096 km2) and the least populous state of India. The alpine zone of Sikkim has unique biodiversity since it also falls in the transition zone of 2 biogeographic regions, namely the central Himalaya region and the southern fringe of the Palearctic region [48]. Lachen valley lies within the alpine zone of Sikkim and shares an international border with the Tibet Autonomous Region (TAR) of China. The valley is among the few places where open grazing is permitted in the state of Sikkim. It is inhabited by two pastoral communities— the yak and sheep herders, the Dokpas and the agro-pastoral Lachenpas. According to the states 2012 livestock census the communities rear 951 heads of cattle, 2588 yaks, 141 horses, 230 sheep (indigenous variety) and 90 goats[49]. Historically both communities conducted trade and practiced pastoralism [50,51]. They used to take their yak and sheep upto Khambazong in TAR, China in summer and traded oil, sugar and fir planks for wool, barley flour (*tsampa*), salt, carpets, mutton and sheep fat. After the 1962 Sino-Indian war the international border was closed off. Today, both communities practice seasonal transhumance. The Lachenpas move to the high elevation pastures in the summer and retreat to lower elevations in the winter, while the Dokpas do the opposite, moving onto the Tibetan Plateau where they find wind-blown, snow-free pastures in winter. Both communities rely on common property resources (pastures, forests, and natural waters) and some pastures on state forest department and military property.

Fig 1: Map of study site with experimental plots. Elevation classes displayed were prepared using Digital Elevation Model (DEM) derived from ASTER data[52].

Vegetation sampling

Herb and graminoid species were sampled in grazing pastures and ungrazed areas. Grazed areas were defined as pastures most often in use and that have been grazed for atleast two decades. The ungrazed areas were defined as areas with a history of no grazing for ateast 10 years with permanent fences around them. These areas are used by the locals to make hay. The herbs s are left to grow in these areas throughout the year and removed at the end of the growing season. Local pastoralists and village representatives were consulted to select grazed and ungrazed areas. I used a randomized block design with two treatments (grazing and ungrazed) and three replicates (lower zone =3000-3500m; middle zone =3500-400m; and higher zone= 4000-4500m) making a total of six 30mX 30m plots (2 treatments X 3 replicates). All experimental data was collected over a two-year period starting from the growing season of 2013 to the end of the growing season in 2015. It was difficult to separate the effects of different grazers since there was much overlap in the grazing areas shared by the different livestock and wild species. Cows, bulls, goats, horses and a few yak shared the same pastures in the lower zone (3000-3500 m asl). Sheep, yak, cows, bulls, goats, and horses shared the middle zones (3500-4000 m asl) and yak, sheep and a few horses grazed in the higher zone (4000-4500 m asl). Thus, I studied the combined effects of different grazing species.

Impacts of grazing on species diversity and above ground net primary productivity (ANPP)

Diversity: In each 30mX30 m plot 12 quadrats (1mX1 m) were randomly laid. We had 36 quadrates in grazed areas (12X 3elevation zones) and 36 in ungrazed areas. Thus, a total of 72 quadrats were laid (24 in each zone/ replicate). Data on species composition and percentage cover was collected from each quadrat for herb species only (forbs and graminoids). The grazing pastures we sampled had a negligible amount of woody vegetation and none fell in our quadrats. In the ungrazed plots there were few shrubs, only along the edges which we anyway avoided to avoid for edge effect

ANPP*:* I followed the difference method by McNaughton et al. [53] to calculate ANPP in the grazed area. Twelve temporary movable exclosure cages, 1mX1m in size, were set up in each grazed plot. Thus a total of 36 movable exclosure cages were setup every month (12 cages in 3 elevation zones). After each sampling effort (monthly) these cages were moved to a new location within the grazed plot to account for biomass growth (over a month). The above ground standing biomass (vegetation) was clipped to ground level and collected inside and outside exclosure cages. The collected plant material was oven dried 24 hours at 60°C and then weighed. The ANPP of grazing plots was calculated using the following formula [53] ANPP= W1g+(W2u-W1g)+(W3u-W2g)+(W4u-W3g)+(W5u-W4g)

where Wi is the dry mass weight of above ground standing biomass at sample time ti (i=1, 2, 3, 4, 5: beginning of June, July, August, September, October respectively), u (ungrazed) and g (grazed) represent samplings inside and outside the exclosure cages, respectively. In ungrazed areas twelve 1mX1m quadrats were laid and biomass was collected at peak standing crop (annually).

Data analysis

Diversity indices—species richness (S), shannon index (H) , simpson diversity index (D) and pielous's evenness (J) were calculated for plant species in grazed and ungrazed quadrats in each elevation zone and for all quadrats (all elevations and grazing treatments combined). Proportional dominance (PD) of a species was calculated as a function of relative density and frequency (cover). $PD = \left(\frac{Ni}{N}\right) * fi$where PD is the proportional dominance of species i, Ni is the percent cover of species i in all locations, N is the percent cover of all species in all locations, and fi is the frequency of plots at which species i occurs. The Kruskal-Wallis test was used to calculate significance for difference between diversity indices and ANPP in grazed and ungrazed areas and different elevation zones.

To compare the differences between assemblages in terms of rarity (Leroy's rarity index [54,55]), heterogeneity of the grassland community(Menhinick index [56]), evenness (McIntosh index [57])and taxonomic distinctness [58,59] (Dstar) the four the components of diversity were combined into a single measurement using the DER_algorithm in the EcoIndR package in R [60]. The algorithm calculates the polar coordinates of all samples with all possible combinations for the four indices and then calculates the convex hull and mean Euclidean distances between

samples. Each circle represents a quadrat (1mX1m), the size of the circles represents species richness and the color rarity.

To explore the response of species composition to grazing treatments and elevation a triangular similarity matrix was constructed using Bray Curtis similarity coefficients based on cover data. The similarity matrix was displayed in an ordination plot using non-metric multidimensional scaling (NMDS). The stress function assesses how well the two-dimensional plot represent actual sample relationships calculated. A lower value (<0.2 commonly accepted) represents a more accurate ordination [61,62].
The Analysis of similarity (ANOSIM) was used to check the degree of dissimilarity of species across grazing treatments, elevation zones, and treatments within each elevation zone. ANOSIM based on the Bray Curtis similarity matrix was used to check significance levels using a permutation test with 999 simulations. R values close to 1 suggest sites within a group are similar to each other and dissimilar to sites in other groups. While an R value close to "0" suggests an even distribution of high and low ranks within and between groups. A linear regression was used to examine the relationship between diversity and productivity.
All analyses were performed on R. The NMDS and ANOSIM analysis were performed using the vegan package in R [63].

Results

Species diversity indices

A total of 153 plant species between 3000-4500 m asl were sampled. This included the 66 (S=13.38 ±5.71),56 (S= 11.42 ±5.01) and 47 (S= 9.38 ±3.41) species found in the lower, middle, and higher zones, respectively. A significantly ($p<0.001$) higher number of species was found in grazed areas (114 species) than in ungrazed areas (61 species). I sampled 51,48 and 30 species in grazed areas and 22,19 and 22 species in ungrazed areas in lower, middle, and higher zones, respectively. The analysis showed all diversity indices (S,H,D and J) were significantly higher ($p<0.001$) in grazed quadrats than ungrazed quadrats in all elevation zones combined and in each elevation zone (Fig 2A-D, table1). Grazing had a significant ($p<0.001$) effect on all diversity indices in each elevation zone and all elevation zones combined (table 1). Unlike grazing, elevation did not have a significant effect on diversity indices (Fig 2F-I))

Table 1: Diversity indices and above ground net primary productivity (average per quadrate ±standard deviation) for plots in grazed and ungrazed areas in the three elevation zones and all elevations combined. P-values (represented by asterixis) indicate difference between grazed and ungrazed areas based on the Kruskal-Wallis. S= Species richness; H= Shannon index; D=Simpsons diversity index; J= Pielou's evenness; ANPP= Above ground net primary productivity.

Elevation Zone	Quadrates	S	H	D	J	ANPP
All elevations combined	All	11.39 ±5.01	2.08 ±0.5	0.83 ±0.09	0.89 ±0.06	756.46 ± 143.33
	Grazed (G)	14.92 ±4.3	2.47 ±0.28	0.9 ±0.03	0.93 ±0.02	861 ±105.63
	Ungrazed (U)	7.86 ±2.61	1.70 ±0.35	0.77 ±0.09	0.84 ±0.05	651.92 ±89.65
	Difference (G-U)	**7.06*** **	**0.77*** **	**0.13*** **	**0.08*** **	**209.08*** **
Lower elevations	All	13.38 ±5.71	2.23 ±0.49	0.85 ±0.07	0.89 ±0.05	829.77 ±144.73
	Grazed (G)	17.83 ±4.3	2.63 ±0.26	0.91 ±0.02	0.92 ±0.02	955.27 ±52.24
	Ungrazed (U)	8.92 ±2.5	1.84 ±0.3	0.80 ±0.58	0.86 ±0.04	704.26 ± 81.87
	Difference (G-U)	**8.91*** **	**0.79*** **	**0.11*** **	**0.06*** **	**251.01*** **
Middle elevations	All	11.42 ±5.01	2.13 ±0.48	0.85 ±0.07	0.90 ±0.04	764.68 ±135.45
	Grazed (G)	15.33 ±3.8	2.53 ±0.24	0.90 ±0.02	0.94 ±0.03	873.76 ±79.28
	Ungrazed (U)	7.50 ±2.11	1.73 ±0.28	0.79 ±0.06	0.88 ±0.04	655.60 ±78.18
	Difference (G-U)	**7.83*** **	**0.8*** **	**0.11*** **	**0.06*** **	**218.16*** **
Higher elevations	All	9.38 ±3.41	1.90 ±0.49	0.80 ±0.12	0.87 ±0.08	674.93 ±107.29
	Grazed (G)	11.58 ±2.15	2.26 ±0.23	0.88 ±0.04	0.93 ±0.03	753.96 ±64.13
	Ungrazed (U)	7.17 ±3.01	1.54 ±0.41	0.72 ±0.13	0.82 ±0.07	595.90 ±79.56
	Difference (G-U)	**4.41*** **	**0.72*** **	**0.16*** **	**0.11*** **	**158.06*** **

P values- <0.001 "***" <0.01 '**' <0.05 '*'.

Results of the ANOSIM indicate statistically significant differences in species composition. There is a significant difference in species composition between grazing treatments in each elevation zone (Lower: R=0.0.911 p<0.001;mid: R= 0.786 p<0.001; higher R=0.878 p<0.001) (Fig 3C-E). As would be expected elevation plays a significant role in species composition. Species composition (grazed and ungrazed combined) in different elevation zones showed a significant difference (R=0.844 p<0.001) (Fig 3B). The ANOSIM R value for grazing treatment across all elevations was relatively lower (R=0.199 p<0.001) suggesting comparatively more overlap in species compositions in grazed and ungrazed areas (Fig 3). Overall, grazed and ungrazed plots had 14.38% (22) species in common. In the lower, middle and higher zones they shared 7(10.61%), 11 (19.64%) and 5 (10.64%) species, respectively.

In addition to S,H,D and J grazing also affected the other components of diversity (Fig 4). Figure 4. shows high dispersion between grazed quadrats and ungrazed quadrats. Grazed quadrat number 5 in the lower zone (G5) showed the highest values of rarity, heterogeneity, evenness and taxonomic distinctness whereas U14 – ungrazed quadrat number 14 in the mid zone showed the lowest values for all indices (Fig 4). As is evidenced from the size of the circle G5 has markedly higher species richness than U14. The color and size of the circles indicate species richness and rarity among grazed plots compared to ungrazed plots. Rarity and richness was higher in grazed quadrats (G) than ungrazed quadrats (Fig 4).

Fig 2. A-J Boxplots of diversity indices and above ground net primary productivity (ANPP) for 153 species sampled from 72 plots. A-E diversity indices and ANPP in grazed vs ungrazed plot in all elevations combined (All) and in each elevation zone (lower, middle and higher). F-J diversity indices and ANPP in each elevation zone for all species. K -ANPP in each elevation zone per month (June to October). L -Linear relationship between species richness (quadrate scale) and ANPP in lower, middle and higher elevation zone quadrates. R^2 values indicated. *** represents statistical significance ($p<0.01$).

Fig 3: Non-metric multidimensional scaling (NMDS) based on Bray Curtis similarity matrix of plant communities. Analysis of similarity (ANOSIM) statistic R and its significance are indicated in boxes. Groups are represented by ellipses (95% CI around centroids). NMDS ordination of 153 species grouped according to grazing treatment (A) and elevation gradient (B); NMDS ordination of species in lower (C), middle (D), and higher elevation (E) zones respectively grouped according to grazing treatment.

Fig 4: Spatial distribution of 72 quadrates based on four diversity indices (rarity- Leroy's rarity index, Menhinick -heterogeneity, Dstar-taxonomic distinctness and McIntosh index-evenness). The size of the circles increases with richness, and a color-code (gradient) is used to indicate the rarity index. Circles are labelled G for grazed quadrates and U for ungrazed quadrates. Numbers in circles represent quadrate numbers (1-12 Lower zone, 13-24 mid zone, 25-36 Higher zone)

Species proportional dominance

All plots were dominated by Graminoid species (Cyperaceae, Juncaceae and Poaceae). They represented 70.58%, 44.34% and 78.19% of PD in all quadrats combined, grazed quadrats and ungrazed quadrats, respectively. These included sixteen species (Cyperaceae n=7, Juncaceae n=2, Poaceae n=7) in total. In grazed quadrats I found thirteen graminoid species (Cyperaceae n=6, Juncaceae n=2, Poaceae n=5) and ten graminoid species (Cyperaceae n=4, Juncaceae n=1, Poaceae n=5) in ungrazed quadrats. Forbs showed high PD in grazed quadrats than ungrazed quadrats with Polygonaceae, Primulaceae, Rosaceae, Asteraceace and Orchidaceae families representing 37.75% of PD.

At the species level *Kobresia nepalensis* (Cyperaceae) was the most dominant species in grazed and ungrazed plots. The five most dominant species in all quadrats combined represented 55.95% of PD. In grazed and ungrazed quadrats the five most dominant species represented 48.09% and 61.18% of dominance, respectively. These included graminoid species in all quadrats combined and ungrazed plots. In grazed plots *Rumex nepalensis* and *Primula dendiculata* were the only forbs representing the five most dominant species the remaining three were represented by graminoid species *Kobresia nepalensis*, *Festuca coelestis* (Poaceae) and *Juncus sp* (Juncaceae).

ANPP

The mean ANPP in all quadrats combined was 756.46 g/m^2 (table 1). Productivity showed a decreasing trend with elevation with the maximum (829.77 g/m^2) in lower zones and minimum (674.93 g/m^2) in the higher elevation quadrats (Fig 2E, table 1). The analysis showed that elevation had a significant ($p<0.001$) role on ANPP.).The mean ANPP was highest in July for all grazed quadrats in lower zone, middle zone and all zones combined but it was highest in August in higher zone (Fig 2K).

quadratsquadratsProductivity was significantly higher in grazed areas than in ungrazed areas (table 1). Overall, the average ANPP in grazed areas was 32.07 % higher than in ungrazed areas. Maximum ANPP (955.27 g/m^2) was found in quadrats laid in the grazed areas in the lower zone and minimum (595.9 g/m^2) in ungrazed areas in the higher zone (table 1). Grazing significantly increased ANPP by 35.64%, 33.28% and 26.52% in the lower, middle and higher zones, respectively. The analysis showed that grazing had a statistically significant effect ($p<0.001$) on productivity within each elevation zone and all elevation zones combined (table 1). The linear regression revealed a statistically significant ($p<0.001$, R^2= 0.457) relationship between species richness and ANPP (Fig 2L).

Discussion

The discussion is divided into three parts. The first two parts address the question how does grazing maintain high species diversity and ecosystem function. First, I discuss how grazing may be a driver for higher species diversityand secondly I discusses the role of species diversity on productivity. Finally, I discuss the socioecological dynamics in the region that have resulted in fewer families investing in pastoralism.

Impact of grazing on species diversity and ANPP

The results demonstrate the importance of the traditional grazing system in maintaining high plant diversity and productivity. Significantly higher diversity indices and productivity in grazed areas suggest plant community composition, structure and function benefit or depend indirectly on grazing for their persistence in the high-elevation alpine pastures of the Himalaya. Grazing may be a driver for higher species diversity in three ways.

First, in free grazing systems like in the study site, higher diversity could be facilitated by the important role livestock play as seed dispersers. Seed dispersal by grazing animals is important

in grasslands throughout the world, being vital for conservation of not only species but genetic diversity [64–66]. Grazing animals are dispersal vectors through epizoochory (transporting outside the body e.g. on fur) or endozoochory (transporting after ingestion). Studies have found higher survival of small seeds in the digestive tract of mammals increasing effectiveness of endozoochory [67–69]. This could explain higher PD of small seeded species such as Orchidaceae and Asteraceae (especially *Anaphalis lactea* and *Lentopodium leontopodioides)* in grazed plots than ungrazed plots

Second, grazing results in the reduction in abundance of dominant species by selectively removing palatable species [70]. This explains higher PD for graminoid species (that are palatable) in ungrazed areas. The results show that graminoid species represent 78.19% of the total PD in ungrazed quadrats as compared to 44.34% in grazed quadrats. Graminoid species of Cyperaceae and Poaceae families were more palatable than Juncaceae which was grazed after most palatable species were eaten. Additionally, higher PD of less palatable families such as Asteraceace, Primulaceae and Rosaceae (dominated by *Potentilla* spp) was found in grazed plots.

Third, grazing can increase spatial heterogeneity thus increasing habitat diversity. Free ranging livestock may modify soil properties directly by recycling nutrients and concentrating it from a larger area into small patches through their dung and urine deposition [71–74]. Grazing may also affect soil properties indirectly through their forage preference and grazing intensity. This may selectively affect the survival of some plants that affect soil properties through their rhizosphere [75,76].

Grazing mediated dispersal, foraging preferences and spatial heterogeneity would increase niche breadth and overlap and may promote patches of rarer species thus increasing diversity.

A strong positive correlation between productivity and rainfall has been well documented across the globe [72,77–79]. In the study site monthly productivity in grazed plots were closely associated with rainfall. The monsoon season in the region starts in May, peaking in July and gradually ending in September and October. Peak ANPP in lower and middle elevation quadrats was reached in July and gradually tapered off with the receding monsoon winds (Fig. 2K). The monsoons gradually travels northwards, towards the higher elevation explaining why the higher elevation quadrats showed a slight delay in peak ANPP (in August) (Fig. 2K). Interestingly the migration pattern of the local communities is closely associated to temporal trends in productivity with the Lachenpas moving up to the lower and middle zones in June and July and the Dokpas moving down to the lower elevations (study design's higher zones) around mid-July. Higher ANPP in grazed plots suggests grazing mediated species diversity is an important driver of ANPP.

Relationship between species diversity and ANPP

A strong positive correlation between diversity and productivity (Fig. 2L) suggests higher biodiversity is an important factor in driving ecosystem functions. The results demonstrating that biomass production increased with diversity conforms to numerous studies from other parts of the world [80–83]. There are three explanations proposed based on experimental studies to

understand the role of diversity on productivity. Firstly, complementarity effects occur when more diverse communities facilitate greater niche partitioning allowing for a more complete utilization of resources resulting in increased biomass production [80,82,84]. Secondly, facilitative effects of some species promote the establishment and survival of other co-occurring species influencing biomass production [81,85]. Thirdly, sampling or selection effects suggesting that more diverse plots have a higher probability of containing species with high biomass[81,83,86,87]. The three effects are not mutually exclusive, rather a combined influence of complementarity, facilitative and selection effect of biodiversity influence biomass in a community.

Studies on the impact of grazing on ecosystems have shown mixed results. Negative effects of grazing have been attributed to high grazing intensity owing to overgrazing and high stocking rates or to low grazing intensity including the abandonment of grazing [2,21,22,72,88,89]. The general consensus is that a light to medium grazing intensity is most beneficial [2,72,90]. This study demonstrates that the pastures in Lachen valley have benefitted from grazing of various livestock suggesting the stocking rates are within or below “carrying capacity”. The traditional rotational grazing system practiced by the communities allow for pasture regrowth and recovery, facilitates resilience and limits degradation. Similar results have been shown by studies from other regions underlining the benefits of rotational grazing [91–94].

Socio ecological systems

Driven by external constraints and the benefits of urbanization, the local communities have diversified toward other sectors, particularly the tourism industry and as porters for the Indian army. The most immediate reason for this change was the complete closure of the international border after the war of 1962 between India and China. This not only ended trade with Tibet, but also led to a loss of access to vast areas of pastures in TAR. In addition to this the establishment of large army encampments and areas for land mines further reduced areas for grazing to an estimated 40% of the original area [95]. Previously, the transboundary migration also ensured the interaction of a larger pool of pastoral people resulting in intermarriages from a larger region as well as more opportunities to cross breed livestock. Today, most Dokpa children are sent to schools in the city and few return to the rigors of a pastoralist’s life. Some Dokpas prefer working as porters and laborers for the Indian army stationed in the region or in the tourism industry as guides, porters, and taxi drivers. Lachenpas too are investing more into the growing tourism industry and less into animal husbandry that show diminishing monetary returns. The loss of grazing pastures and access to trade, reduced opportunities for marriage and cross-breeding livestock, and the exposure to modern education and urbanization have resulted in a reduction in Dokpa population living in the region from 30 households (130 persons) in the 1950’s [95,96] to just sixteen families [96]. Fewer Dokpas and Lachenpas investing in pastoralism would eventually result in lesser livestock numbers.

Conclusion

The paper demonstrates that grazing enhances species diversity and productivity indicating that the pastures in the region have benefited from grazing by livestock. The results are significant in two respects. First, it suggests that a complete ban on grazing, a common practice in protected areas of developing countries [97–99] may not meet its primary objective of conserving

biodiversity. Second, the socioeconomic losses because of the grazing ban can be reduced by reintroducing grazing after sufficient research on altered herd sizes and the impacts of specific livestock and grazing intensities. The results suggest that a management strategy that is mutually beneficial for conservation and livelihoods is not just possible but desirable to maintain higher local and regional biodiversity.

Moreover, crucial role of the traditional grazing system on biodiversity and ecosystem function, coupled with disinvestment in pastoralism by the locals poses an imminent threat to the landscape that is an intricate socio-ecological system with a long history of co-adaptation with ruminant animal. Pastoralism is associated with other spillover effects such as illegal timber and fuelwood collection, medicinal plants (often rare and endemic) collection and the negative effects of overgrazing as enumerated by Bhagwat et al. [41]. At the same time this study shows, the absence of grazing may result in loss of biodiversity and reduced ecosystem function. The benefits of the traditional free ranging pastoralism cannot be ignored, and a holistic approach is needed to conserve the rich biodiversity of the region. As studies from the region demonstrate the grazing ban has resulted in reduced incomes [41], increase in monocultures in lowlands, decreased manure production in a state that exclusively practices organic farming [39], spread of gregarious species and perceived increase in human wildlife conflict [41,43].
I conclude with research and policy recommendations that should be part of an improved grazing management policy in the state.

1. Experimental studies reintroducing grazing in small areas in regions where the grazing ban was enacted need to be conducted. Studies with the aim to understand the role of different livestock species and different grazing intensities on biodiversity structure and function need to be conducted.
2. Climate change is among the biggest threats to Himalayan rangelands, a region of immense significance for ecosystem services provided to atleast one fifth of humanity and among the most among the most biodiverse in the world [34,37]. Further research on the role of grazing on combating shrub encroachment in response to climate change that threatens habitat loss is imperative.
3. The present grazing ban policy needs to be revisited with a more holistic approach
    a. To understand the role of altered herd sizes on diversity and ecosystem function. A complete ban of grazers meets neither conservation nor livelihood goals. A comprehensive approach taking into account social, ecological, and economic strategies would be required to meet sustainable goals.
    b. Explore a payment for ecosystem services schemes for the services provided by pastoralists in maintaining biodiversity and ecosystem function. These schemes should be aimed at pastoralists moving away from practicing traditional pastoralism. Similar schemes have been used in Switzerland where "*general direct payments*" are made for upland farmers to encourage grazing livestock in more difficult conditions [100].
    c. Explore the link between the grazing ban policy and increasing incidences of human wildlife conflict as reported by Bhagwat et al. [41]. An efficient compensation

scheme for human wildlife conflict that is affecting marginalized communities needs to be developed.

d. Enact reforms to improve livelihoods of ex-herders who are products of the grazing ban policy. While the government has made efforts to reduce this economic impact yet Gupta et al. [39] report frustration among ex-herders in response to "*a lack of reform to improve ex-herders' livelihoods*" post ban.

Acknowledgements

I would like to thank Dr. Kamaljit Bawa for offering vital comments and edits and Dr. Mahesh Sankaran for his advice on the experimental design. Dharmendra Lamsal assisted with parts of the data collection.. I would like to thank the Lachenpa and Dokpa communities of Lachen valley and the Dzumsa for their support and enthusiasm. I would also like to thank reviewer 3 for suggesting the DER algorithm and offering vital comments.

References

1. Williams RE, Allred BW, DeNio RM, Paulsen Jr HA. Conservation, Development, and Use of the World's Rangelands. J Range Manag. 1968;21: 355–360. Available: https://www.jstor.org/stable/pdf/3896081.pdf

2. Zhang R, Wang Z, Han G, Schellenberg MP, Wu Q, Gu C. Grazing induced changes in plant diversity is a critical factor controlling grassland productivity in the Desert Steppe, Northern China. Agric Ecosyst Environ. 2018;265: 73–83. doi:10.1016/J.AGEE.2018.05.014

3. Havstad KM, Peters DC, Allen-Diaz B, Bartolome J, Bestelmeyer BT, Briske D, et al. The western United States rangelands, a major resource. In: Wedin WF, Fales SL, editors. Grassland Quietness and Strength for a New American Agriculture. Madison, WI: American Society of Agronomy Inc., Crop Science Society of America Inc., and Soil Science Society of America Inc; 2009. pp. 75–93.

4. Neely C, Bunning S, Wilkes A. Review of evidence on drylands pastoral systems and climate change. Rome: Food and Agriculture Organization of the United Nations, 2009; 2009.

5. Costanza R, d'Arge R, de Groot R, Farber S, Grasso M, Hannon B, et al. The value of the world's ecosystem services and natural capital. Nature. 1997;387: 253–260. doi:10.1038/387253a0

6. Sayre NF, McAllister RR, Bestelmeyer BT, Moritz M, Turner MD. Earth Stewardship of rangelands: coping with ecological, economic, and political marginality. Front Ecol Environ. 2013;11: 348–354. doi:10.1890/120333

7. Farley KA, Walsh K, Levine AS. Opportunities and obstacles for rangeland conservation in San Diego County, California, USA. Ecol Soc. 2017;22: 38.

8. Sala OE, Yahdjian L, Havstad K, Aguiar MR. Rangeland ecosystem services: nature's supply and humans' demand. In: Briske DD, editor. Rangeland systems: Processes, management and challenges. Springer International Publishing, Cham; 2017. pp. 467–489.

9. Brunson MW, Huntsinger L. Ranching as a Conservation Strategy: Can Old Ranchers Save the New West? Rangel Ecol Manag. 2008;61: 137–147. doi:10.2111/07-063.1

10. Alkemade R, Reid RS, van den Berg M, de Leeuw J, Jeuken M. Assessing the impacts of livestock production on biodiversity in rangeland ecosystems. Proc Natl Acad Sci U S A. 2013;110: 20900–5. doi:10.1073/pnas.1011013108

11. Little PD. Pastoralism, biodiversity, and the shaping of savanna landscapes in East Africa. Africa (Lond). 1996;66: 37–51. doi:10.2307/1161510

12. Miller DJ. Nomads of the Tibetan Plateau Rangelands in Western China. Part One: Pastoral History. Rangelands. 1998;20: 24–29.

13. Niamir-Fuller M, Huber-Sannwald E. Pastoralism and Achievement of the 2030 Agenda for Sustainable Development: A Missing Piece of the Global Puzzle. In: Lucatello S, Huber-Sannwald E, Espejel I, Martínez-Tagüeña N, editors. Stewardship of Future Drylands and Climate Change in the Global South. Springer, Cham; 2020. pp. 41–55. doi:10.1007/978-3-030-22464-6_3

14. Niamir-Fuller M. Managing mobility in African rangelands. Niamir-Fuller M, editor. London, UK: Intermediate Technology Publications; 1999.

15. Goldman M, Nadasdy P, Turner M, editors. Knowing nature: conversations at the intersection of political ecology and science studies. Chicago, IL: University of Chicago Press; 2011.

16. Sandford S, Scoones I. Improving the efficiency of opportunism: new directions for pastoral development. In: Scoones I, editor. Living with uncertainty: new directions in pastoral development in Africa. London, UK: Intermediate Technology Publications; 1994.

17. Banks T. Property Rights Reform in Rangeland China: Dilemmas On the Road to the Household Ranch. World Dev. 2003;31: 2129–2142. doi:10.1016/J.WORLDDEV.2003.06.010

18. Liu X, Ma Z, Huang X, Li L. How does grazing exclusion influence plant productivity and community structure in alpine grasslands of the Qinghai-Tibetan Plateau? Glob Ecol Conserv. 2020;23: e01066. doi:10.1016/J.GECCO.2020.E01066

19. Western D, Groom R, Worden J. The impact of subdivision and sedentarization of pastoral lands on wildlife in an African savanna ecosystem. Biol Conserv. 2009;142: 2538–2546. doi:10.1016/J.BIOCON.2009.05.025

20. Weber KT, Horst S. Desertification and livestock grazing: The roles of sedentarization, mobility and rest. Pastor Res Policy Pract. 2011;1: 19. doi:10.1186/2041-7136-1-19

21. Camacho O, Dobremez L, Capillon A. Shrub encroachment in pastures in the Alps. Rev géographie Alp. 2008; 89–100. doi:10.4000/rga.575

22. Zhang W, Miller PA, Smith B, Wania R, Koenigk T, Döscher R. Tundra shrubification and tree-line advance amplify arctic climate warming: results from an individual-based dynamic vegetation model. Environ Res Lett. 2013;8: 034023. doi:10.1088/1748-9326/8/3/034023

23. Post E, Pedersen C. Opposing plant community responses to warming with and without herbivores. Proc Natl Acad Sci U S A. 2008;105: 12353–8. doi:10.1073/pnas.0802421105

24. Olofsson J, Oksanen L, Callaghan T, Hulme P., Oksanen T, Suominen O. Herbivores inhibit climate-driven shrub expansion on the tundra. Glob Chang Biol. 2009;15: 2681–2693. doi:10.1111/j.1365-2486.2009.01935.x

25. Myers-Smith IH, Forbes BC, Wilmking M, Hallinger M, Lantz T, Blok D, et al. Shrub expansion in tundra ecosystems: dynamics, impacts and research priorities. Environ Res Lett. 2011;6: 045509. doi:10.1088/1748-9326/6/4/045509

26. Verma M, Schulte to Bühne H, Lopes M, Ehrich D, Sokovnina S, Hofhuis SP, et al. Can reindeer husbandry management slow down the shrubification of the Arctic? J Environ Manage. 2020;267: 110636. doi:10.1016/J.JENVMAN.2020.110636

27. Mittermeier RA, Turner WR, Larsen FW, Brooks TM, Gascon C. Global Biodiversity Conservation: The Critical Role of Hotspots. In: Zachos FE, Habel JC, editors. Biodiversity Hotspots: Distribution and Protection of Conservation Priority Areas. Berlin, Heidelberg: Springer Berlin Heidelberg; 2011. pp. 3–22. doi:10.1007/978-3-642-20992-5_1

28. Ning W, Rawat G, Joshi S, Ismail M, Sharma E, editors. High-altitude rangelands and their interfaces in the Hindu Kush Himalayas. Kathmandu: ICIMOD; 2013.

29. Shrestha UB, Gautam S, Bawa KS. Widespread climate change in the Himalayas and associated changes in local ecosystems. PLoS One. 2012;7: e36741. doi:10.1371/journal.pone.0036741

30. Telwala Y, Brook BW, Manish K, Pandit MK. Climate-induced elevational range shifts and increase in plant species richness in a Himalayan biodiversity epicentre. PLoS One. 2013;8: e57103. doi:10.1371/journal.pone.0057103

31. Pandey R, Khadka KK, Ghimire A, Jha P, Pathak U. Elevational distribution of butterflies in the Himalayas: a case study from Langtang National Park, Nepal. J Mt Sci. 2017;14: 1384–1390. doi:10.1007/s11629-017-4360-9

32. Tiwari A, Fan Z-X, Jump AS, Li S-F, Zhou Z-K. Gradual expansion of moisture sensitive Abies spectabilis forest in the Trans-Himalayan zone of central Nepal associated with climate change. Dendrochronologia. 2017;41: 34–43. doi:10.1016/J.DENDRO.2016.01.006

33. Anderson K, Fawcett D, Cugulliere A, Benford S, Jones D, Leng R. Vegetation expansion in the subnival Hindu Kush Himalaya. Glob Chang Biol. 2020; gcb.14919. doi:10.1111/gcb.14919

34. Xu J, Grumbine RE, Shrestha A, Eriksson M, Yang X, Wang Y, et al. The melting Himalayas: cascading effects of climate change on water, biodiversity, and livelihoods.

Conserv Biol. 2009;23: 520–30. doi:10.1111/j.1523-1739.2009.01237.x

35. Singh C., Paniprahy S, Thapliyal A, Kimothi M., Soni P, Parihar J. Monitoring the alpine treeline shift in parts of the Indian Himalayas using remote sensing. Curr Sci. 2012;102: 559–562.

36. Gaire N., Koirala M, Bhuju D., Borgaonkar H. Treeline dynamics with climate change at the central Nepal Himalaya. Clim Past. 2014;10: 1277–1290. Available: http://moeseprints.incois.gov.in/876/1/Treeline dynamics.pdf

37. Telwala Y. Climate change and alpine flora in Sikkim Himalaya. Climate Change in Sikkim - Patterns, Impacts and Initiatives. 2012. pp. 103–124.

38. Tambe S, Rawat GS. Ecology, economics, and equity of the pastoral systems in the Khangchendzonga National Park, Sikkim Himalaya, India. Ambio. 2009;38: 95–100. doi:10.1579/0044-7447-38.2.95

39. Gupta R, Haider LJ, Österblom H. The theory of cross-scale interactions: an illustration from remote villages in Sikkim, India. Environ Dev Sustain. 2020;22: 3777–3804. doi:10.1007/s10668-019-00329-0

40. Feroze SM, Ray LI, Singh KJ, Singh R. Pastoral yak rearing system is changing with change in climate: an exploration of North Sikkim in Eastern Himalaya. Clim Change. 2018;157: 483–498.

41. Bhagwat S, Diwan M, Venkataramani V. Study of ecological, eocio-economic and livelihood dimensions of grazing exclusion in preotected forests of West Sikkim. Chennai; 2012.

42. Bhagwat S, Diwan M, Venkataramani V. Analysis of Grazing Exclusion Policy through a Climate Change Mitigation Lens: Case Studies from Barsey Rhododendron Sanctuary,West Sikkim. In: Arrawatia ML, Tambe S, editors. Climate Change in Sikkim - Patterns, Impacts and Initiatives. Gangtok: Information and Public Relations Department Government of Sikkim; 2012. pp. 351–368. Available: http://www.sikkimforest.gov.in/climate-change-in-sikkim/21-Chapter_Analysis of Grazing Exclusion Policy through the climate change.pdf

43. Tambe S, Bhutia N, Arrawatia M. People’s opinion on the Impacts of “Ban on Grazing” in Barsey Rhododendron Sanctuary. Sikkim, India. Gangtok, Sikkim; 2005.

44. Singh P, Chauhan A. Plant Diversity in Sikkim Himalaya. In: Hajra P, Mugda V, editors. Plant Diversity and Hotspots in India. Calcutta: BSI; 1997. pp. 137–162.

45. Ingty T, Bawa KS. Climate change and indigenous people. In: Arrawatia ML, Tambe S, editors. Climate Change in Sikkim Patterns, Impacts and Initiatives. Information and Public Relations Department,Government of Sikkim, Gangtok; 2012. pp. 275–290.

46. Briggs JM, Knapp AK. Interannual variability in primary production in tallgrass prairie: climate, soil moisture, topographic position, and fire as determinants of aboveground biomass. Am J Bot. 1995;82: 1024–1030.

47. Smith MD, Knapp AK. Dominant species maintain ecosystem function with non-random

species loss. Ecol Lett. 2003;6: 509–517.

48. Rodgers WA, Panwar HS. Planning a wildlife protected area network in India. Wildlife Institute of India, Dehradun; 1988. Available: http://agris.fao.org/agris-search/search.do?recordID=XF9093847

49. Government of Sikkim. 19th Livestock Census, 2012. II. Gangtok, Sikkim; 2012. Available: http://www.sikkim-ahvs.gov.in/Download/Report2.pdf

50. Ingty T. High mountain communities and climate change: adaptation, traditional ecological knowledge, and institutions. Clim Change. 2017;145: 41–55. doi:10.1007/s10584-017-2080-3

51. Joshi S, Shrestha L, Bisht N, Wu N, Ismail M, Dorji T, et al. Ethnic and Cultural Diversity amongst Yak Herding Communities in the Asian Highlands. Sustainability. 2020;12: 957. doi:10.3390/su12030957

52. LP DAAC. ASTER Global Digital Elevation Model (GDEM) v2. NASA EOSDIS Land Processes DAAC,. Sioux Falls, South Dakota; 2014.

53. McNaughton SJ, Milchunas DG, Frank DA. How can net Primary Productivity be Measured in Grazing Ecosystems? Ecology. 1996;77: 974–977. doi:10.2307/2265518

54. Leroy B, Petillon J, Gallon R, Canard A, Ysnel F. Improving occurrence- based rarity metrics in conservation studies by including multiple rarity cut- off points. Insect Conserv Divers. 2012;5: 159-168.

55. Guisande C, Heine J, García-Roselló E, González-Dacosta J, Vilas LG, Perez-Schofield BJG. DER: An algorithm for comparing species diversity between assemblages. Ecol Indic. 2017;81: 41–46. doi:https://doi.org/10.1016/j.ecolind.2017.05.049

56. Menhinick E. A Comparison of Some Species- Individuals Diversity Indices Applied to Samples of Field Insects. Ecology. 1964;45: 859–861.

57. McIntosh R. "An index of diversity and the relation of certain concepts to diversity. Ecology. 1967;48: 392–404.

58. Warwick RM, Clarke KR. Taxonomic distinctness and environmental assessment. J Appl Ecol. 1998;35: 532–543.

59. Clarke KR, Warwick RM. A taxonomic distinctness index and its statistical properties. J Appl Ecol. 1998;35: 523–531.

60. R Development Core Team. A Language and Environment for Statistical Computing. R Foundation for Statistical Computing. Vienna, Austria; 2017. Available: http://www.r-project.org/

61. Tyler CL, Kowalewski M. Utility of Marine Benthic Associations as a Multivariate Proxy of Paleobathymetry: A Direct Test from Recent Coastal Ecosystems of North Carolina. PLoS One. 2014;9: e95711. doi:10.1371/JOURNAL.PONE.0095711

62. Dexter E, Rollwagen-Bollens G, Bollens SM. The trouble with stress: A flexible method for the evaluation of nonmetric multidimensional scaling. Limnol Oceanogr Methods.

2018;16: 434–443. doi:10.1002/lom3.10257

63. Oksanen J, Blanchet FG, Kindt R, Legendre P, Minchin P., O'Hara R., et al. Vegan: Community Ecology Package. R Package Version 2.2-0. 2014.

64. Janzen DH. Dispersal of Small Seeds by Big Herbivores: Foliage is the Fruit. Am Nat. 1984;123: 338–353. Available: http://copa.acguanacaste.ac.cr:8080/bitstream/handle/11606/1223/DISPERSAL OF SMALL SEEDS BY BIG HERBIVORES FOLIAGE IS THE FRUIT.pdf?sequence=1

65. Couvreur M, Christiaen B, Kris V, Martin V. Large herbivores as mobile links between isolated nature reserves through adhesive seed dispersal. Appl Veg Sci. 2004;7: 229–236. Available: https://www.biw.kuleuven.be/lbh/lbnl/ecology/pdf-files/pdf-art/martine/Couvreuretal_Epizoochory_2004_AVS7 1 .229-236.pdf

66. Plue J, Aavik T, Cousins SAO. Grazing networks promote plant functional connectivity among isolated grassland communities. van Kleunen M, editor. Divers Distrib. 2019;25: 102–115. doi:10.1111/ddi.12842

67. Pakeman RJ, Digneffe G, Small JL. Ecological Correlates of Endozoochory by Herbivores. Funct Ecol. 2002;16: 296–304. Available: http://www.jstor.org/stable/826582

68. Cocyns E, Delporte A, Lens L, Hoffman M. Germination success of temperate grassland species after passage through ungulate and rabbit guts. J Ecol. 2005;93: 353–361. doi:10.1111/j.0022-0477.2005.00982.x

69. Wu G-L, Li X-P, Cheng J-M, Wei X-H, Sun L. Grazing Disturbances Mediate Species Composition of Alpine Meadow Based on Seed Size. Isr J Ecol Evol. 2009;55: 369–379. doi:10.1560/IJEE.55.4.369

70. Lu X, Kelsey KC, Yan Y, Sun J, Wang X, Cheng G, et al. Effects of grazing on ecosystem structure and function of alpine grasslands in Qinghai-Tibetan Plateau: a synthesis. Ecosphere. 2017;8: e01656. doi:10.1002/ecs2.1656

71. Carran RA, Ball PR, Theobald PW, Collins MEG. Soil nitrogen balances in urine-affected areas under two moisture regimes in Southland. New Zeal J Exp Agric. 1982;10: 377–381. doi:10.1080/03015521.1982.10427902

72. McNaughton SJ. Ecology of a Grazing Ecosystem: The Serengeti. Ecol Monogr. 1985;55: 259–294. doi:10.2307/1942578

73. Steinauer EM, Collins SL. Effects of Urine Deposition on Small-Scale Patch Structure in Prairie Vegetation. Ecology. 1995;76: 1195–1205. doi:10.2307/1940926

74. Liu C, Song X, Wang L, Wang D, Zhou X, Liu J, et al. Effects of grazing on soil nitrogen spatial heterogeneity depend on herbivore assemblage and pre-grazing plant diversity. Wan S, editor. J Appl Ecol. 2016;53: 242–250. doi:10.1111/1365-2664.12537

75. Hamilton EW, Frank DA. Can plants simulate soil microbes and their own nutrient supply? Evidence from a grazing tolerant grass. Ecology. 2001;82: 2397–2402. doi:10.1890/0012-9658(2001)082[2397:CPSSMA]2.0.CO;2

76. Wilson CH, Strickland MS, Hutchings JA, Bianchi TS, Flory SL. Grazing enhances belowground carbon allocation, microbial biomass, and soil carbon in a subtropical grassland. Glob Chang Biol. 2018;24: 2997–3009. doi:10.1111/gcb.14070

77. Pandey CB, Singh JS. Rainfall and Grazing Effects on Net Primary Productivity in a Tropical Savanna, India. Ecology. 1992;73: 2007–2021. doi:10.2307/1941451

78. Fay PA, Carlisle JD, Knapp · Alan K., Blair JM, Collins SL. Productivity responses to altered rainfall patterns in a C4-dominated grassland. Oecologia. 2003;137: 245–251. Available: https://www.researchgate.net/profile/John_Blair2/publication/10674171_Productivity_responses_to_altered_rainfall_patterns_in_a_C_4_-dominated_grassland/links/0fcfd50855628aff47000000.pdf

79. Castro SM, Sanchez-Azofeifa GA, Sato H. Effect of drought on productivity in a Costa Rican tropical dry forest. Environ Res Lett. 2018;13: 045001. Available: https://iopscience.iop.org/article/10.1088/1748-9326/aaacbc/pdf

80. Hector A. The Effect of Diversity on Productivity: Detecting the Role of Species Complementarity. Oikos. 1998;82: 597. doi:10.2307/3546380

81. Kelemen A, Török P, Valkó O, Deák B, Tóth K, Tóthmérész B. Both facilitation and limiting similarity shape the species coexistence in dry alkali grasslands. Ecol Complex. 2015;21: 34–38. doi:10.1016/J.ECOCOM.2014.11.004

82. Guerrero-Ramírez NR, Craven D, Reich PB, Ewel JJ, Isbell F, Koricheva J, et al. Diversity-dependent temporal divergence of ecosystem functioning in experimental ecosystems. Nat Ecol Evol. 2017;1: 1639–1642. doi:10.1038/s41559-017-0325-1

83. Sonkoly J, Kelemen A, Valkó O, Deák B, Kiss R, Tóth K, et al. Both mass ratio effects and community diversity drive biomass production in a grassland experiment. Sci Rep. 2019;9: 1848. doi:10.1038/s41598-018-37190-6

84. Reich PB, Tilman D, Isbell F, Mueller K, Hobbie SE, Flynn DFB, et al. Impacts of Biodiversity Loss Escalate Through Time as Redundancy Fades. Science (80-). 2012;336: 589 LP – 592. doi:10.1126/science.1217909

85. Stachowicz JJ. Mutualism, Facilitation, and the Structure of Ecological CommunitiesPositive interactions play a critical, but underappreciated, role in ecological communities by reducing physical or biotic stresses in existing habitats and by creating new habitats on which many species depend. Bioscience. 2001;51: 235–246. doi:10.1641/0006-3568(2001)051[0235:mfatso]2.0.co;2

86. Huston MA. Hidden treatments in ecological experiments: re-evaluating the ecosystem function of biodiversity. Oecologia. 1997;110: 449–460. doi:10.1007/s004420050180

87. Loreau M. Separating Sampling and Other Effects in Biodiversity Experiments. Oikos. 1998;82: 600–602. Available: https://www.jstor.org/stable/pdf/3546381.pdf?casa_token=Tyj4lVMsMtYAAAAA:nQeur7AJMI64xit1hB_n_XPixj3AEELNPF4PmPgtHD1KvXtkMnKTZgAfh-FSOZ4MYlzvwdkrfc8FvwFn323FiQNq8r_WKQtDz7BvpaM_M9MS8pyeWA

88. Haynes MA, Fang Z, Waller DM. Grazing impacts on the diversity and composition of alpine rangelands in Northwest Yunnan. J Plant Ecol. 2013;6: 122–130. doi:10.1093/jpe/rts021

89. Li X, Liu Z, Wang Z, Wu X, Li X, Hu J, et al. Pathways of Leymus chinensis Individual Aboveground Biomass Decline in Natural Semiarid Grassland Induced by Overgrazing: A Study at the Plant Functional Trait Scale. Wang W, editor. PLoS One. 2015;10: e0124443. doi:10.1371/journal.pone.0124443

90. Pulungan MA, Suzuki S, Gavina MKA, Tubay JM, Ito H, Nii M, et al. Grazing enhances species diversity in grassland communities. Sci Rep. 2019;9: 11201. doi:10.1038/s41598-019-47635-1

91. Brundage AL, Petersen WE. A Comparison Between Daily Rotational Grazing and Continuous Grazing. J Dairy Sci. 1952;35: 623–630. doi:10.3168/jds.S0022-0302(52)93747-8

92. Teague WR, Dowhower SL. Patch dynamics under rotational and continuous grazing management in large, heterogeneous paddocks. J Arid Environ. 2003;53: 211–229. doi:10.1006/JARE.2002.1036

93. Vecchio MC, Bolaños VA, Golluscio RA, Rodríguez AM. Rotational grazing and exclosure improves grassland condition of the halophytic steppe in Flooding Pampa (Argentina) compared with continuous grazing. Rangel J. 2019;41: 1–12. Available: https://doi.org/10.1071/RJ18016

94. Jacobo EJ, Rodríguez AM, Bartoloni N, Deregibus VA. Rotational Grazing Effects on Rangeland Vegetation at a Farm Scale. Rangel Ecol Manag. 2006;59: 249–257. doi:10.2111/05-129R1.1

95. Sharma G, Tambe S, Rawat GS, Arrawatia M. Yak Herding and Associated Transboundary Issues in the Sikkim Himalaya, India. Transboundary challenges and opportunities for yak raising in a changing Hindu Kush Himalayan region. Kathmandu, Nepal: ICIMOD; 2016. pp. 93–112.

96. NBSAP. Sikkim State Biodiversity Strategy and Action Plan. Gangtok, Sikkim; 2002.

97. Lewis M. Cattle and Conservation at Bharatpur: A Case Study in Science and Advocacy. Conserv Soc. 2003;1: 1–21. Available: https://www.jstor.org/stable/26396448?seq=1#metadata_info_tab_contents

98. Dong SK, Gao HW, Xu GC, Hou XY, Long RJ, Kang MY, et al. Farmer and professional attitudes to the large-scale ban on livestock grazing of grasslands in China. Environ Conserv. 2007;34: 246–254. Available: http://www.jstor.org/stable/44521238

99. Gurung B, Nelson KC, Smith JLD. Impact of Grazing Restrictions on Livestock Composition and Husbandry Practices in Madi Valley, Chitwan National Park, Nepal. Environ Conserv. 2009;36: 338–347. Available: https://www.jstor.org/stable/44520031?seq=1#metadata_info_tab_contents

100. Swiss Federal Office for Agriculture. Swiss Agricultural Policy: Objectives, tools, prospects. Bern, Switzerland; 2004. Available: https://www.cbd.int/financial/pes/swiss-

pesagriculturalpolicy.pdf

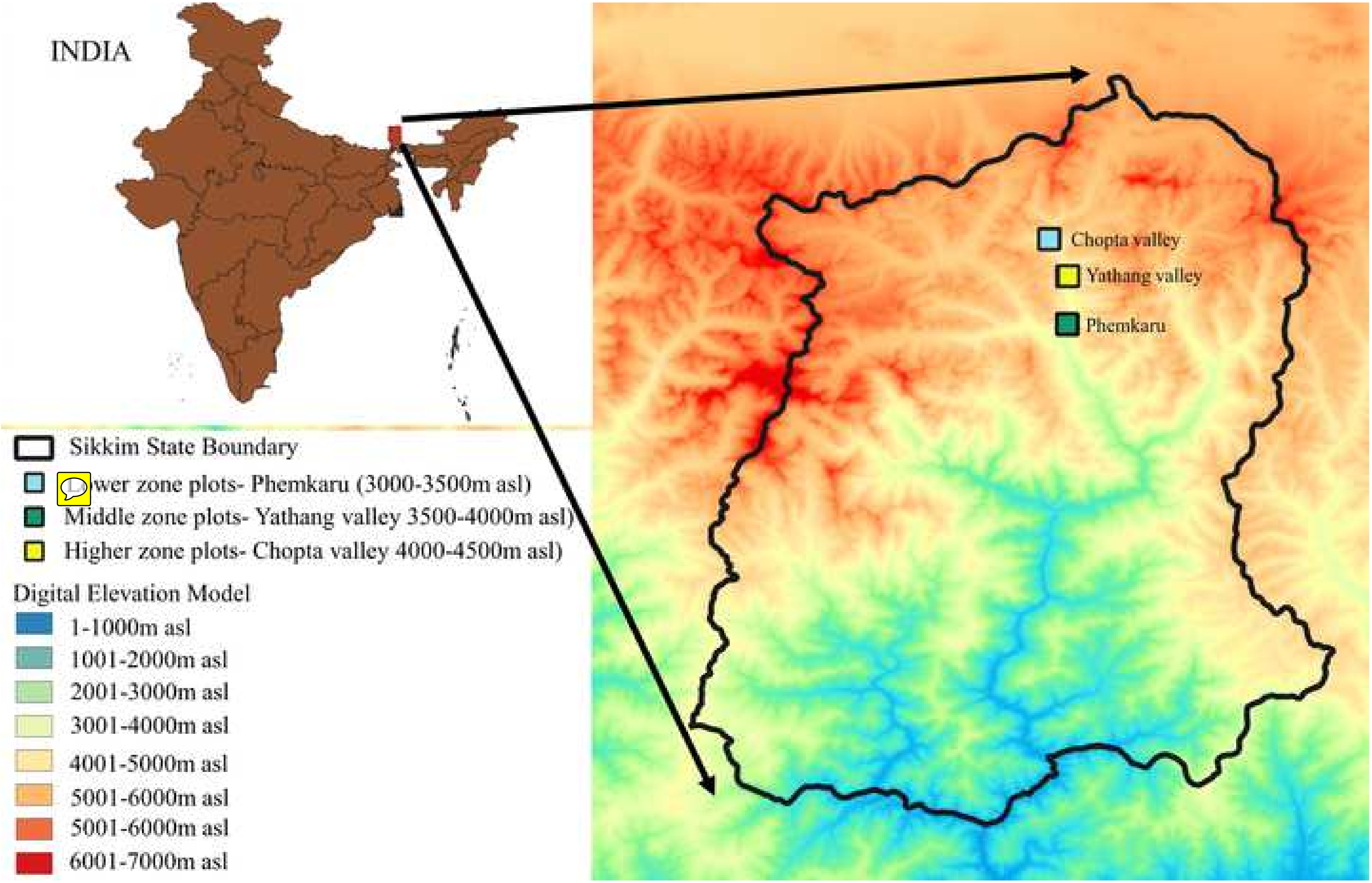

INDIA
Sikkim State Boundary
wer zone plots- Phemkaru (3000-3500m asl)
Middle zone plots- Yathang valley 3500-4000m asl)
Higher zone plots- Chopta valley 4000-4500m asl)
Digital Elevation Model
1-1000m asl
1001-2000m asl
2001-3000m asl
3001-4000m asl
4001-5000m asl
5001-6000m asl
5001-6000m asl
6001-7000m asl
Chopta valley
Yathang valley
Phemkaru

Fig 2

Click here to access/download;Figure;FIG2.tif

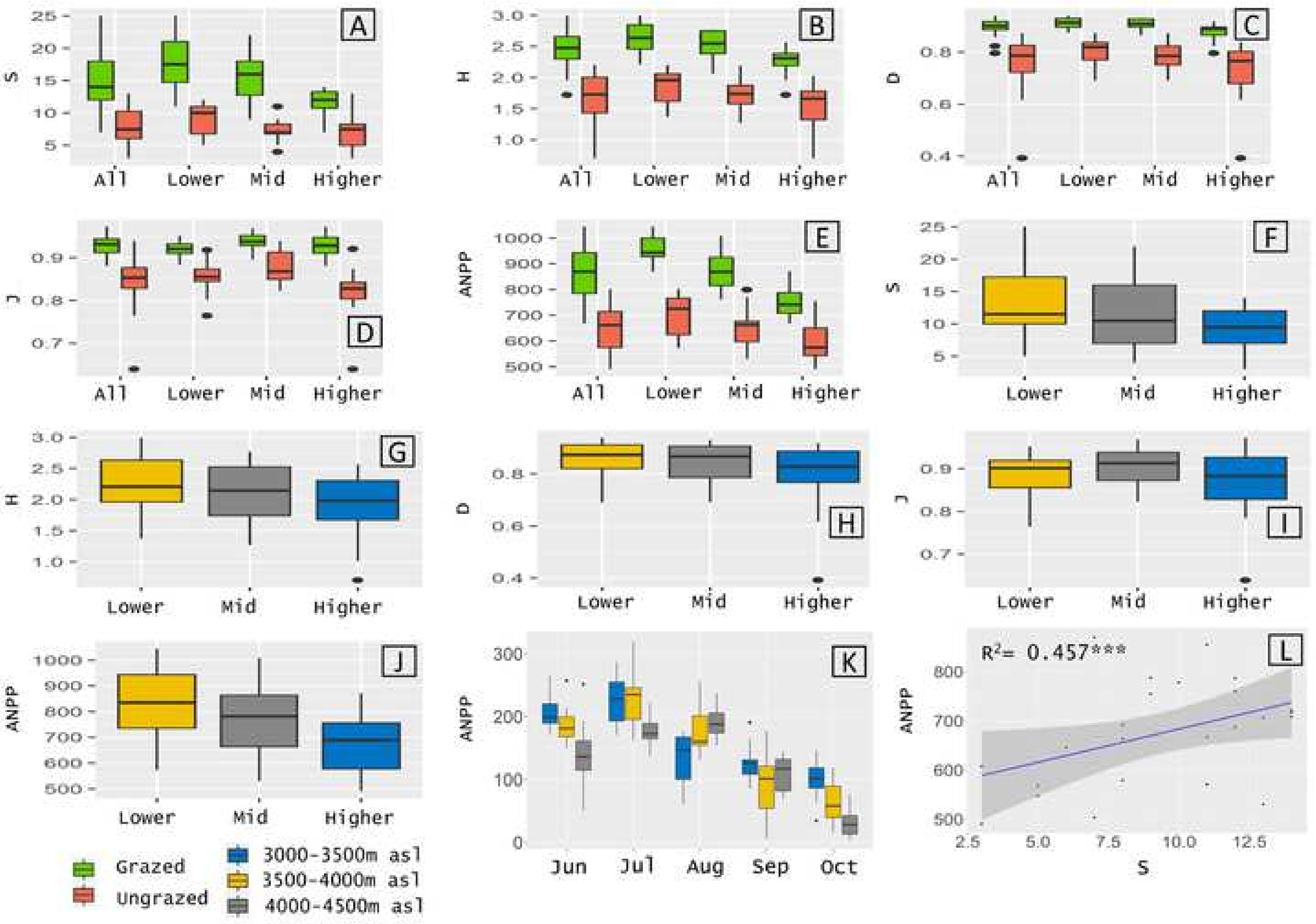

Fig 3

Click here to access/download;Figure;FIG3.tif

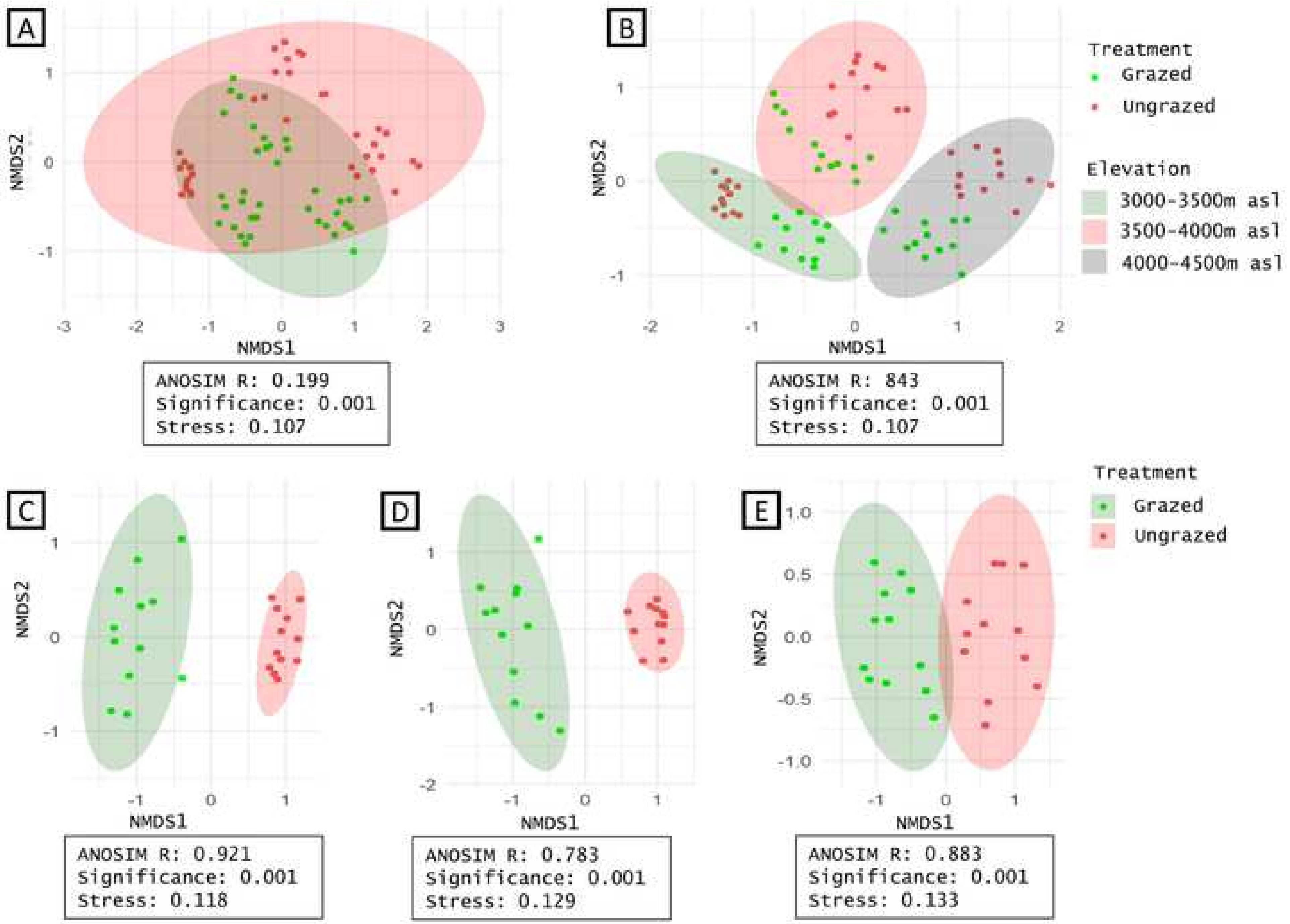

Figure 4

Click here to access/download;Figure;Fig 4.jpg

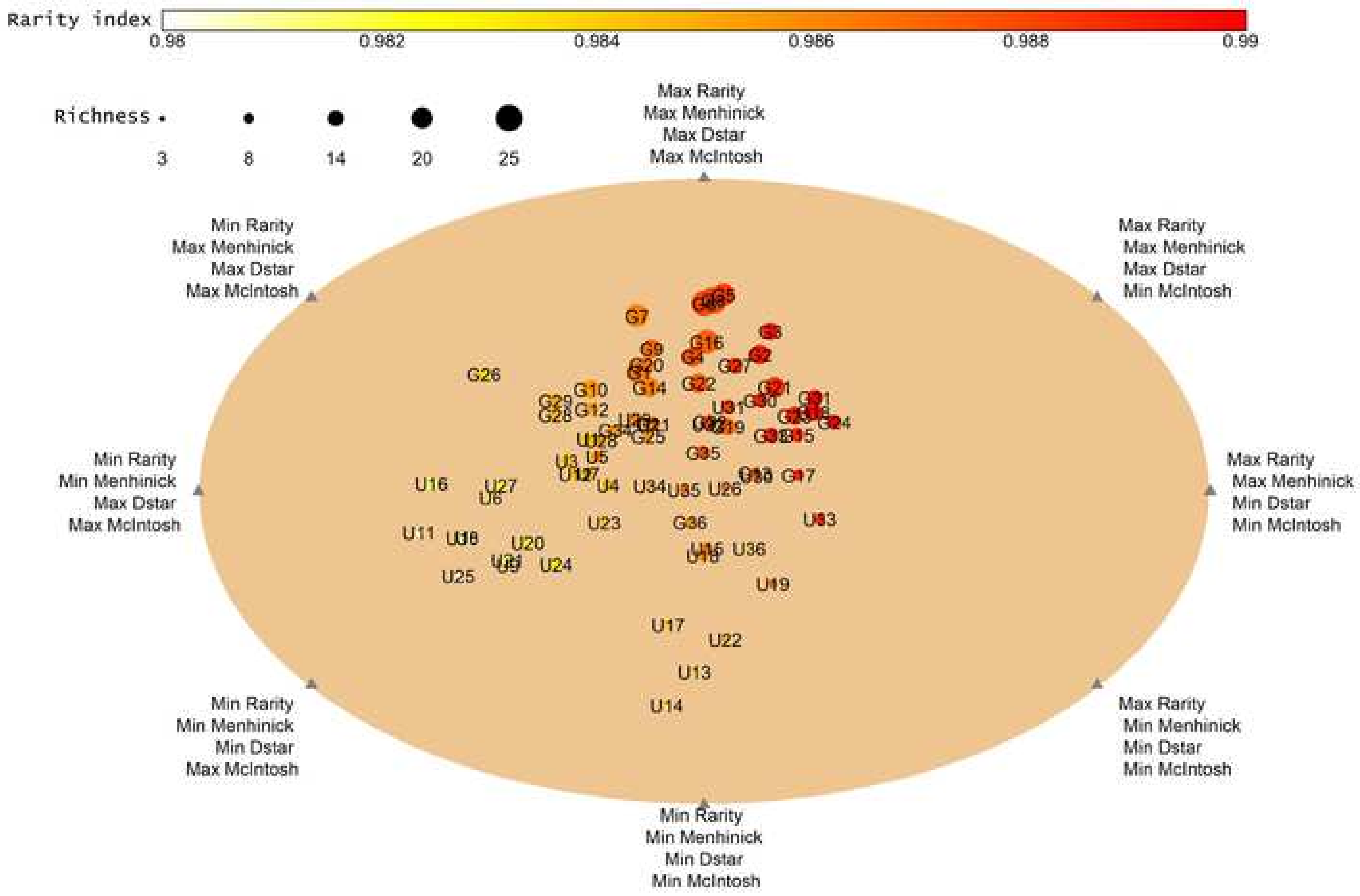

Supplementary material

Click here to access/download
Supporting Information
Supplementary material.docx

Pastoralism in the highest peaks: Role of the traditional grazing systems in maintaining biodiversity and ecosystem function in the alpine Himalaya.

Tenzing Ingty[1]
[1]*Department of Biology, University of Massachusetts, Boston, MA*

tenzingingty@gmail.com

Abstract

Rangelands cover around half of the planet's land mass and provide vital ecosystem services to over a quarter of humanity. The Himalayan rangelands, part of a global biodiversity hotspot is among the most threatened regions in the world. In rangelands of many developing nations policies banning grazing in protected areas is common practice. In 1998, the Indian state of Sikkim, in the Eastern Himalaya, enacted a grazing ban in response to growing anthropogenic pressure in pastures and forests that was presumably leading to degradation of biodiversity. Studies from the region demonstrate the grazing ban has had some beneficial results in the form of increased carbon stocks and regeneration of some species of conservation value but the ban also resulted in negative outcomes such as reduced household incomes, increase in monocultures in lowlands, decreased manure production in a state that exclusively practices organic farming, spread of gregarious species, and a perceived increase in human wildlife conflict. This paper explores the impact of the traditional pastoral system on high elevation plant species in Lachen valley, one of the few regions of Sikkim where the grazing ban was not implemented. Experimental plots were laid in along an elevation gradient in grazed and ungrazed areas. Ungrazed areas are part of pastures that have been fenced off (preventing grazing) for over a decade and used by the locals for hay formation. I quantified plant species diversity (Species richness, Shannon index, Simpson diversity index, and Pielou evenness index) and ecosystem function (above ground net primary productivity ANPP). The difference method using movable exlosure cages was used in grazing areas to account for plant ANPP eaten and regrowth between grazing periods). The results demonstrate that grazing significantly contributes to greater plant species diversity (Species richness, Shannon index, Simpson diversity index, and Pielou evenness index) and ecosystem function (using above ground net primary productivity as an indicator). The multidimensional scaling and ANOSIM (Analysis of Similarities) pointed to significant differences in plant species assemblages in grazed~~ing~~ and ungrazed areas($P<0.001$). Further, ecosystem function is controlled by grazing, rainfall and elevation. Thus, the traditional transhumant pastoral system may enhance biodiversity and ecosystem function. I argue that a complete restriction of open grazing meet neither conservation nor socioeconomic goals. Evidence based policies are required to conserve the rich and vulnerable biodiversity of the region.

Formatted: Font: 18 pt
Formatted: Centered
Formatted: Superscript
Formatted: Left
Formatted: Superscript
Formatted: Font: (Default) Times New Roman, 18 pt, Bold, Font color: Auto
Formatted: Font: (Default) Times New Roman, 18 pt, Bold
Formatted: Heading 1, Adjust space between Latin and Asian text, Adjust space between Asian text and numbers

Keywords

grazing ban policy, rangelands, species diversity, productivity, Himalaya, alpine pastures

Introduction

Rangelands cover approximately half of earth's land surface [1,2] and over a quarter of humanity depend on these land types for their livelihoods and numerous ecosystem services [3,4]. The ecosystem services include provisioning services like food, fiber and water, and supporting services such as carbon storage (roughly 30% of global soil carbon) among many others [5–8]. Rangelands are also recognized for their high biodiversity, including numerous endangered and endemic species, as well as habitat connectivity between protected forests [6,7,9,10]. In much of the developing world, rangelands are inhabited by pastoral communities. Pastoralism that has existed for millennia has impacted the evolution of many rangelands, from the biodiverse rich African savanna [11] to the highest elevations in the Tibetan steppe [12]. Pastoralists and the rangelands they inhabit thus form complex socio-ecological systems that have co-evolved across different geographies, biodiversity and climates [11,13]. Many pastoralists have been blamed, often wrongly, for rangeland degradation [14,15] and targeted for "*modernization*" programs such as sedenterization. While some of these programs have been beneficial [6,16–18], most have shown to have socially and ecologically disastrous impacts resulting in a decline in wildlife, decreased grassland diversity and productivity, and increased desertification [14,19,20]. Moreover, the abandonment of rangelands by pastoralists and climate change is leading to rapid shrub invasion, further increasing habitat loss [21,22]. Grazing by herbivores such as the semi domesticated reindeer has been shown to counteract the effect of warming on shrub expansion [23–26]. This suggests that grazing management is an important factor to maintain alpine areas in response to climate change driven shrub invasion.

Rangelands in the Himalaya, a global biodiversity hotspot [27], form the largest land use system and provides numerous ecosystem services to pastoral communities [28]. The Himalaya is the source of 10 of the largest rivers in Asia whose basins feed about one fifth of humanity. The alpine rangelands of the Himalaya thus play a vital role in retaining critical ecosystem services such as carbon sequestration, water storage and provisioning, ~~and~~ maintaining biodiversity, and food security and livelihoods. Despite its importance a report on high ~~altitude~~ elevation rangelands of the Himalaya [28] underline numerous threats such as invasive species, inappropriate management and development policies, overgrazing and climate change. Studies show that climate change has significantly impacted the Himalaya. These include an increase in average temperatures three times higher than the global average, altered phenology and shifts in distribution ranges of biodiversity [29–33]. Upward shifts in treelines and shrubs have led to woody ingression into alpine pastures [34–36].

The alpine rangelands of the tiny state of Sikkim in the Eastern Himalaya are among the most biodiverse regions in the world. They occupy just 0.006% of global alpine rangelands but harbor as much as 60% of the global alpine plant families and 10% (297 genera) of global alpine genera with more than twice the number of vascular plants than the Alps and Rockies [37]. In 1998 the government of Sikkim adopted a grazing exclusion policy in response to growing anthropogenic

Formatted: Font: (Default) Times New Roman, 18 pt, Bold, Font color: Auto

Formatted: Heading 1, Adjust space between Latin and Asian text, Adjust space between Asian text and numbers

Formatted: Font: (Default) Times New Roman, 18 pt, Bold

Formatted: Font: (Default) Times New Roman, 18 pt, Bold, Font color: Auto

Formatted: Heading 1

Formatted: Font: (Default) Times New Roman, 18 pt, Bold

pressure on grazing lands and protected forests leading to perceived degradation. Open grazing was phased out over the next six years in all reserved forests with exception of alpine pastures in the Kangchenzonga National Park [38,39], Lachen and Lachung valleys [40].~~ and alpine pastures in the Kangchenzonga National Park~~. The forest cover of the state between 1999 to 2017 increased by about 4% from 3118 km^2 to 3379 km^2. Studies in Barsey Rhododendron Sanctuary in West Sikkim estimate that twelve years after the grazing ban was declared carbon stocks increased by about 585 thousand tonnes and the concurrent increase in regeneration of species of high conservation value such as *Rhododendron barbatum*~~*barbetum*~~ [41,42]. At the same time, Bhagwat et al. [41] also warned of insignificant regeneration of important fodder species and other species of high conservation value such as *Quercus spp.* coupled with a spread of gregarious species such as *Arundinaria maling* and *Viburum cordifolium*. Restriction on free access to forest resources have had significant socio-economic impacts on the herders who were dependent on grazing pastures in the sanctuary. Their average net income from livestock was reduced by more than half the pre-ban period, with the source of majority of the incomes now coming from government subsidies and schemes like the National Rural Employment Guarantee Act programme (MGNREGA) for unskilled labor [41,43]. A study on the perception of sedenterized ex-herders from West Sikkim identified loss of traditional livelihoods leading to decreased income and limited land holdings as their main protestations. In addition to socio-economic impacts to herders the grazing ban has had a cascading impact on non-herders[41]. Perceptions of local villagers indicate an increase in ~~in~~ human wildlife conflict in the form of crop raids and livestock depredation with over 85% of the households experiencing greater losses in agricultural outputs after the grazing ban [41,43].

Here, I explore the impact of the present pastoral system on high elevation plant species in Lachen valley, ~~one of the few regions of~~ Sikkim~~ where the grazing ban was not implemented~~. The region is of significance for two reasons. First, it falls in one of the most biodiverse regions of the world, the alpine zone of the Sikkim Himalaya [37]. Second transhumant pastoralism has been practiced in the region for centuries with the indigenous communities directly dependent on the biodiversity of their surrounding for livestock husbandry, water storage and provisioning, and the collection of medicinal, aromatic, and edible plants [38,44,45].

I address 2 broad questions: ~~in the alpine pastures of the Himalaya.~~

1. What is the impact of the traditional grazing system on plant ~~bio~~diversity and ecosystem function (using aboveground net primary productivity as an integrative measure of ecosystem function[46,47]) in the high-~~altitude~~ elevation alpine Himalaya?
2. How does grazing maintain biodiversity and ecosystem function in the region?

I first analyze the impact of grazing on ~~species~~ diversity and productivity. I then discuss the role of grazing in maintaining high biodiversity and ecosystem function. Finally, I conclude by identifying further knowledge needs and propose recommendations for an improved grazing policy to protect the rich yet vulnerable biodiversity of the region.

~~The results demonstrate that grazing enhances species diversity and productivity indicating that the pastures in the region have benefited from grazing by livestock. The results are significant in two respects. First, the results suggest that a complete ban on grazing, a common practice in protected areas of developing countries [46–48] may not meet its primary objective of conserving biodiversity. Second, the socioeconomic losses because of the grazing ban can be~~

reduced by reintroducing grazing after sufficient research on altered herd sizes and the impacts of specific livestock and grazing intensities. The results suggest that a management strategy that is mutually beneficial for conservation and livelihoods is not just possible but desirable to maintain higher local and regional biodiversity.

Materials and methods

Study area

(Field Research. Permit number: 03592280402 Department of Forest, Environment & Wildlife Management, Government of Sikkim, India.)

: The study was conducted in Lachen valley in the North district of the state of Sikkim in India. Sikkim, in the Eastern Himalayan region (figFig. 1), is the second smallest (7096 km2) and the least populous state of India. The alpine zone of Sikkim has unique biodiversity since it also falls in the transition zone of 2 biogeographic regions, namely the central Himalaya region and the southern fringe of the Palearctic region [48]. Lachen valley lies within the alpine zone of Sikkim and shares an international border with the Tibet Autonomous Region (TAR) of China. The valley is among the few places where open grazing is permitted in the state of Sikkim. It is inhabited by two pastoral communities— the yak and sheep herders, the Dokpas and the agro-pastoral Lachenpas. According to the states 2012 livestock census the communities rear 951 heads of cattle, 2588 yaks, 141 horses, 230 sheep (indigenous variety) and 90 goats[49]. Historically both communities conducted trade and practiced pastoralism [50,51]. They used to take their yak and sheep upto Khambazong in TAR, China in summer and traded oil, sugar and fir planks for wool, barley flour (*tsampa*), salt, carpets, mutton and sheep fat. After the 1962 Sino-Indian war the international border was closed off. Today, both communities practice seasonal transhumance. The Lachenpas move to the high elevation pastures in the summer and retreat to lower altitudes elevations in the winter, while the Dokpas do the opposite, moving onto the Tibetan Plateau where they find wind-blown, snow-free pastures in winter. Both communities rely on common property resources (pastures, forests, and natural waters) and some pastures on state forest department and army military property.

Fig 1: Map of study site with experimental plots. Elevation classes displayed were prepared using Digital Elevation Model (DEM) derived from ASTER data[52].

Vegetation sampling:

Plant Herb and graminoid species species were sampled in grazing pastures and ungrazed areas. Grazed areas were defined as pastures most often in use and that have been grazed for atleast two decades. The ungrazed areas were defined as areas with a history of no grazing for ateast 10

Formatted: Font: (Default) Times New Roman, 18 pt, Bold, Font color: Auto

Formatted: Font: (Default) Times New Roman, 18 pt, Bold

Formatted: Heading 1

Formatted: Heading 2 Char, Font: (Default) Times New Roman, 16 pt, Not Bold, Font color: Text 1

Formatted: Font: 12 pt

Formatted: Font: (Default) Times New Roman, 12 pt

Formatted: Font: (Default) Times New Roman

Formatted: Font: Bold, Font color: Text 1

Formatted: Heading 2 Char, Font: Not Bold

Formatted: Font: Bold

Formatted: Font: (Default) Times New Roman, 12 pt

Formatted: Font: (Default) Times New Roman, 12 pt

Formatted: Font: Bold

Formatted: Font color: Text 1

Formatted: Heading 2 Char, Font: (Default) Times New Roman, 16 pt, Not Bold, Font color: Text 1

Formatted: Heading 2 Char, Font: 16 pt, Not Bold, Font color: Text 1

Formatted: Font: Bold, Font color: Text 1

Formatted: Heading 2 Char, Font: 16 pt

years with permanent fences around them. These areas are used by the locals to make hay. The herbs ~~and gras~~s are left to grow in these areas throughout the year and removed at the end of the growing season. Local pastoralists and village representatives were consulted to select grazed and ungrazed areas. I used a randomized block design with two treatments (grazing and ungrazed) and three replicates (lower zone =3000-3500m; middle zone =3500-400m; and higher zone= 4000-4500m) making a total of six 30mX 30m plots (2 treatments X 3 replicates). ~~I followed a randomized block design with three replicates laid along an elevation gradient (lower elevation zone =3000-3500m; middle elevation zone =3500-400m; and higher elevation zone= 4000-4500m asl). Each block contained both grazed and ungrazed areas. Within each block a 30x30 m plot was demarcated.~~ All experimental data was collected over a two-year period starting from the growing season of 2013 to the end of the growing season in 2015. It was difficult to separate the effects of different grazers since there was much overlap in the grazing areas shared by the different livestock and wild species. Cows, bulls, goats, horses and a few yak shared the same pastures in the lower ~~elevation~~ zone (3000-3500 m asl). Sheep, yak, cows, bulls, goats, and horses shared the middle ~~elevation~~ zones (3500-4000 m asl) and yak, sheep and a few horses grazed in the higher ~~elevation~~ zone (4000-4500 m asl). Thus, I studied the combined effects of different grazing species.

Impacts of grazing on species diversity and above ground net primary productivity (ANPP)

Diversity: In each 30mX30 m plot 12 quadrats (1mX1 m) were randomly laid. We had 36 quadrates in grazed areas (12X 3elevation zones) and 36 in ungrazed areas. Thus, a total of 72 quadrats were laid (24 in each zone/ replicate). Data on species composition and percentage cover was collected from each quadrat~~In each 30X30 m plot 24 quadrates (1X1 m) were randomly laid (12 in grazed areas and 12 in ungrazed areas). Thus, total of 72 1x1 m quadrats with 24 quadrates were laid in each elevation zone. Data on species composition and percentage cover was collected from each quadrat~~ for herb species only (forbs and graminoids). The grazing pastures we sampled had a negligible amount of woody vegetation and none fell in our quadrats. In the ungrazed plots there were few shrubs, only along the edges which we anyway avoided to avoid for edge effect

ANPP*:* I followed the difference method by McNaughton et al. [53] to calculate ANPP in the grazed area. Twelve temporary movable exclosure cages, ~~1x1m~~ 1mX1m in size, were set up in ~~the~~ each grazed ~~area~~plot. Thus a total of 36 movable exclosure cages were setup every month (12 cages in 3 elevation zones). After each sampling effort (monthly) these cages were moved to a new location within the grazed plot~~grazed location~~ to account for biomass growth (over a month). The above ground standing biomass (vegetation) was clipped to ground level and collected ~~of was sampled~~ inside and outside exclosure cages. The collected plant material was oven dried 24 hours at 60°C and then weighed. The ANPP of grazing plots was calculated using the following formula [53] ANPP= W1g+(W2u-W1g)+(W3u-W2g)+(W4u-W3g)+(W5u-W4g)

where Wi is the dry mass weight of above ground standing biomass at sample time ti (i=1, 2, 3, 4, 5: beginning of June, July, August, September, October respectively), ~~.~~ u (ungrazed) and g (grazed) represent samplings inside and outside the exclosure cages, respectively. In ungrazed

Formatted: Font color: Text 1

Formatted: Font: Not Italic, Font color: Text 1

Formatted: Font color: Text 1

Formatted: Font: 16 pt, Bold, Not Italic, Font color: Auto

Formatted: Heading 2

Formatted: Font color: Text 1

Formatted: Font: Italic

areas twelve ~~1x1m~~ 1mX1m quadrats were laid and biomass was collected at peak standing crop (annually).

Data analysis~~:~~

Diversity indices—species richness (S), shannon index (H) , simpson diversity index (D) and pielous's evenness (J) were calculated for plant species in grazed and ungrazed ~~quadrates~~quadrats in each elevation zone and for all ~~quadrates~~quadrats (all elevations and grazing treatments combined). Proportional dominance (PD) of a species was calculated as a function of relative density and frequency (cover). ~~PD = (Ni/N) *fi~~ $PD = \left(\frac{Ni}{N}\right) * fi$

where PD is the proportional dominance of species i, Ni is the percent cover of species i in all locations, N is the percent cover of all species in all locations, and fi is the frequency of plots at which species i occurs. The ~~Welch's two Sample t-~~Kruskal-Wallis test was used to calculate significance for difference between diversity indices and ANPP in grazed and ungrazed areas and different elevation zones. ~~(table 1). An analysis of variance (ANOVA) was used to determine the effect of grazing and elevation on species diversity indices and ANPP (table 2). The ANOVA was conducted as a complete randomized block design with grazing treatment, elevation and their interaction taken as fixed effects. The Shapiro-Wilk test and Levene's test were used to check the residuals of analysis for normality and for homogeneity, respectively.~~

To compare the differences between assemblages in terms of rarity (Leroy's rarity index [54,55]), heterogeneity of the grassland community(Menhinick index [56]), evenness (McIntosh index [57])and taxonomic distinctness [58,59] (Dstar) the four the components of diversity were combined into a single measurement using the DER_algorithm in the EcoIndR package in R [60]. The algorithm calculates the polar coordinates of all samples with all possible combinations for the four indices and then calculates the convex hull and mean Euclidean distances between samples. Each circle represents a quadrat (1mX1m), the size of the circles represents species richness and the color rarity.

To explore the response of species composition to grazing treatments and elevation a triangular similarity matrix was constructed using Bray Curtis similarity coefficients based on cover data. The similarity matrix was displayed in an ordination plot using non-metric multidimensional scaling (NMDS). The stress function assesses how well the two-dimensional plot represent actual sample relationships calculated. A lower value (<0.2 commonly accepted) represents a more accurate ordination [61,62].

The Analysis of similarity (ANOSIM) was used to check the degree of dissimilarity of species across grazing treatments, elevation zones, and treatments within each elevation zone. ANOSIM based on the Bray Curtis similarity matrix was used to check significance levels using a permutation test with 999 simulations. R values close to 1 suggest sites within a group are similar to each other and dissimilar to sites in other groups. While an R value close to "0" suggests an even distribution of high and low ranks within and between groups. A linear regression was used to examine the relationship between diversity and productivity.

All analyses were performed on R. The NMDS and ANOSIM analysis were performed using the vegan ~~function~~ package in R [63].

Formatted: Heading 2 Char, Font: 16 pt, Not Bold, Font color: Auto

Formatted: Heading 2, Left

Formatted: Font: 16 pt

Formatted: Font:

Formatted: Justified

Formatted: Font color: Text 1

Formatted: Font color: Text 1

Formatted: Font color: Text 1

Formatted: Font color: Text 1

Formatted: Font color: Text 1

Formatted: Font color: Text 1

Formatted: Font color: Text 1

Formatted: Font color: Text 1

Formatted: Font color: Text 1

Formatted: Font color: Text 1

Formatted: Font color: Text 1

Results

Species diversity indices

~~: ~~A total of 153 plant species between 3000-4500 m asl were sampled. This included the 66 (S=13.38 ±5.71),56 (S= 11.42 ±5.01) and 47 (S= 9.38 ±3.41) species found in the lower, middle, and higher ~~elevation~~ zones, respectively. A significantly (p<0.001) higher number of species was found in grazed areas (114 species) than in ungrazed areas (61 species). I sampled 51,48 and 30 species ~~were sampled~~ in grazed areas and 22,19 and 22 species in ungrazed areas in lower, middle, and higher ~~elevation~~ zones, respectively. The analysis showed all diversity indices (S,H,D and J) were significantly higher (p<0.001) in grazed ~~quadrates~~quadrats than ungrazed ~~quadrates~~quadrats in all elevation zones combined and in each elevation zone (~~fig~~ Fig 2A-D, table1). Grazing had a significant (~~ANOVA~~ p<0.001) effect on all diversity indices in each elevation zone and all elevation zones combined (table ~~2~~1). ~~-~~Unlike grazing, elevation did not have a significant effect on diversity indices (Fig 2F-I))~~Unlike grazing, elevation did not have a significant effect (ANOVA p<0.05)diversity indices (fig. 2G-I, table 2) except S (fig. 2F, table 2).~~

Formatted: Font: (Default) Times New Roman, 18 pt, Bold, Font color: Auto

Formatted: Font: (Default) Times New Roman, 18 pt, Bold

Formatted: Heading 1

Formatted: Heading 2 Char, Font: 16 pt, Not Bold, Font color: Text 1

Formatted: Heading 2 Char, Font: 16 pt, Not Bold, Font color: Text 1

Formatted: Font: Bold, Font color: Text 1

Formatted: Heading 2 Char, Font: 16 pt, Not Bold

Formatted: Font: (Default) Times New Roman, 12 pt

Formatted: Font: (Default) Times New Roman, 12 pt

Table 1: Diversity indices and above ground net primary productivity (average per quadrate ±standard deviation) for plots in grazed and ungrazed areas in the three elevation zones and all elevations combined. P-values (represented by asterixis) indicate difference between grazed and ungrazed areas based on the Kruskal-Wallis. S= Species richness; H= Shannon index; D=Simpsons diversity index; J= Pielou's evenness; ANPP= Above ground net primary productivity.

Formatted: Font: Bold

Formatted: Font: 12 pt

**Elevation Zone**	**Quadrates**	**S**	**H**	**D**	**J**	**ANPP**
All elevations combined	All	11.39 ±5.01	2.08 ±0.5	0.83 ±0.09	0.89 ±0.06	756.46 ± 143.33
	Grazed (G)	14.92 ±4.3	2.47 ±0.28	0.9 ±0.03	0.93 ±0.02	861 ±105.63
	Ungrazed (U)	7.86 ±2.61	1.70 ±0.35	0.77 ±0.09	0.84 ±0.05	651.92 ±89.65
	Difference (G-U)	**7.06*****	**0.77*****	**0.13*****	**0.08*****	**209.08*****
Lower elevations	All	13.38 ±5.71	2.23 ±0.49	0.85 ±0.07	0.89 ±0.05	829.77 ±144.73
	Grazed (G)	17.83 ±4.3	2.63 ±0.26	0.91 ±0.02	0.92 ±0.02	955.27 ±52.24
	Ungrazed (U)	8.92 ±2.5	1.84 ±0.3	0.80 ±0.58	0.86 ±0.04	704.26 ± 81.87
	Difference (G-U)	**8.91*****	**0.79*****	**0.11*****	**0.06*****	**251.01*****
Middle elevations	All	11.42 ±5.01	2.13 ±0.48	0.85 ±0.07	0.90 ±0.04	764.68 ±135.45
	Grazed (G)	15.33 ±3.8	2.53 ±0.24	0.90 ±0.02	0.94 ±0.03	873.76 ±79.28
	Ungrazed (U)	7.50 ±2.11	1.73 ±0.28	0.79 ±0.06	0.88 ±0.04	655.60 ±78.18
	Difference (G-U)	**7.83*****	**0.8*****	**0.11*****	**0.06*****	**218.16*****
Higher elevations	All	9.38 ±3.41	1.90 ±0.49	0.80 ±0.12	0.87 ±0.08	674.93 ±107.29
	Grazed (G)	11.58 ±2.15	2.26 ±0.23	0.88 ±0.04	0.93 ±0.03	753.96 ±64.13
	Ungrazed (U)	7.17 ±3.01	1.54 ±0.41	0.72 ±0.13	0.82 ±0.07	595.90 ±79.56
	Difference (G-U)	**4.41*****	**0.72*****	**0.16*****	**0.11*****	**158.06*****

P values- <0.001 "***" <0.01 '**' <0.05 '*'.

Formatted: Font: 12 pt

Results of the ANOSIM indicate statistically significant differences in species composition. There is a significant difference in species composition between grazing treatments ~~with~~in each elevation zone (Lower: R=0.0.911 p<0.001;mid: R= 0.786 p<0.001; higher R=0.878 p<0.001) (~~fig~~Fig~~.~~ 3C-E). As would be expected elevation plays a significant role in species composition. Species composition (grazed and ungrazed combined) in different elevation zones showed a significant difference (R=0.844 p<0.001) (~~fig~~Fig~~.~~ 3B). The ANOSIM R value for grazing treatment across all elevations was relatively lower (R=0.199 p<0.001) suggesting comparatively more overlap in species compositions in grazed and ungrazed areas (~~fig~~Fig~~.~~ 3). Overall, grazed and ungrazed plots had 14.38% (22) species in common. In the lower, middle and higher zones they shared 7(10.61%), 11 (19.64%) and 5 (10.64%) species, respectively.

In addition to S,H,D and J grazing also affected the other components of diversity (Fig 4). Figure 4. shows high dispersion between grazed quadrats and ungrazed quadrats. Grazed quadrat number 5 in the lower zone (G5) showed the highest values of rarity, heterogeneity, evenness

and taxonomic distinctness whereas U14 – ungrazed quadrat number 14 in the mid zone showed the lowest values for all indices (Fig 4). As is evidenced from the size of the circle G5 has markedly higher species richness than U14. The color and size of the circles indicate species richness and rarity among grazed plots compared to ungrazed plots. Rarity and richness was higher in grazed quadrats (G) than ungrazed quadrats (Fig 4).

Fig 2. A-J Boxplots of diversity indices and above ground net primary productivity (ANPP) for 153 species sampled from 72 plots. A-E diversity indices and ANPP in grazed vs ungrazed plot in all elevations combined (All) and in each elevation zone (lower, middle and higher). F-J diversity indices and ANPP in each elevation zone for all species. K -ANPP in each elevation zone per month (June to October). L -Linear relationship between species richness (quadrate scale) and ANPP in lower, middle and higher elevation zone quadrates. R^2 values indicated. *** represents statistical significance (p<0.01).

Fig 3: Non-metric multidimensional scaling (NMDS) based on Bray Curtis similarity matrix of plant communities. Analysis of similarity (ANOSIM) statistic R and its significance are indicated in boxes. Groups are represented by ellipses (95% CI around centroids). NMDS ordination of 153 species grouped according to grazing treatment (A) and elevation gradient (B); NMDS ordination of species in lower (C), middle (D), and higher elevation (E) zones respectively grouped according to grazing treatment.

Fig 4: Spatial distribution of 72 quadrates based on four diversity indices (rarity- Leroy's rarity index, Menhinick -heterogeneity, Dstar-taxonomic distinctness and McIntosh index-evenness). The size of the circles increases with richness, and a color-code (gradient) is used to indicate the rarity index. Circles are labelled G for grazed quadrates and U for ungrazed quadrates. Numbers in circles represent quadrate numbers (1-12 Lower zone, 13-24 mid zone, 25-36 Higher zone)

Species proportional dominance~~:~~

All plots were dominated by Graminoid species (Cyperaceae, Juncaceae and Poaceae). They represented 70.58%, 44.34% and 78.19% of PD in all ~~quadrates~~quadrats combined, grazed ~~quadrates~~quadrats and ungrazed ~~quadrates~~quadrats, respectively. These included sixteen species (Cyperaceae n=7, Juncaceae n=2, Poaceae n=7) in total. In grazed ~~quadrates~~quadrats I found thirteen graminoid species (Cyperaceae n=6, Juncaceae n=2, Poaceae n=5) and ten graminoid species (Cyperaceae n=4, Juncaceae n=1, Poaceae n=5) in ungrazed ~~quadrates~~quadrats. Forbs showed high PD in grazed ~~quadrates~~quadrats than ungrazed ~~quadrates~~quadrats with Polygonaceae, Primulaceae, Rosaceae, Asteraceace and Orchidaceae families representing 37.75% of PD.

Formatted: Font: Bold

Formatted: Font: Bold

Formatted: Font: Bold

Formatted: Font: (Default) Arial, Pattern: Clear (White)

Formatted: Heading 2

Formatted: Font: (Default) Times New Roman, 16 pt, Bold

At the species level *Kobresia nepalensis* (Cyperaceae) was the most dominant species in grazed and ungrazed plots. The ~~5~~ five most dominant species in all ~~quadrates~~quadrats combined represented 55.95% of PD. In grazed and ungrazed ~~quadrates~~quadrats the five most dominant species represented 48.09% and 61.18% of dominance, respectively. These included graminoid species in all ~~quadrates~~quadrats combined and ungrazed plots. In grazed plots *Rumex nepalensis* and *Primula dendiculata* were the only forbs representing the five most dominant species the remaining ~~3~~ three were represented by graminoid species *Kobresia nepalensis*, *Festuca coelestis* (Poaceae) and *Juncus sp* (Juncaceae).

ANPP~~:~~

~~-~~The mean ANPP in all ~~quadrates~~quadrats combined was 756.46 g/m^2 (table 1). Productivity showed a decreasing trend with elevation with the maximum (829.77 g/m^2) in lower ~~elevation~~ zones and minimum (674.93 g/m^2) in the higher elevation ~~quadrates~~quadrats (Fig 2E, table 1). The analysis ~~for variance~~ showed that elevation had a significant (p<0.001) role on ~~(ANOVA p<0.001) effect on~~ ANPP. ~~(table 2)~~.~~-~~The mean ANPP was highest in July for all grazed quadrats in lower zone, middle zone and all zones combined but it was highest in August in higher zone (Fig 2K).

~~The mean ANPP for all grazed quadrates (all elevation zones combined), lower and middle elevation zone grazed quadrates~~quadrats ~~was highest in July (Fig 2K) and in the higher elevation zone grazed quadrates~~quadrats ~~in August (Fig 2K).~~

Productivity was significantly higher in grazed areas than in ungrazed areas (table 1). Overall, the average ANPP in grazed areas was 32.07 % higher than in ungrazed areas. Maximum ANPP (955.27 g/m^2) was found in quadrats laid in the grazed areas in the lower ~~elevation~~ zone and minimum (595.9 g/m^2) in ungrazed areas in the higher ~~elevation~~ zone (table 1). Grazing significantly increased ANPP by 35.64%, 33.28% and 26.52% in the lower, middle and higher ~~elevation~~ zones, respectively. The analysis showed that grazing had a statistically significant effect (~~ANOVA p~~P<0.001) on productivity within each elevation zone and all elevation zones combined (table 1 ~~2~~). The linear regression revealed a statistically significant ($p<0.001$, R^2= 0.457) relationship between species richness and ANPP (Fig 2L).

Discussion

The discussion is divided into three parts. The first two parts address the question how does grazing maintain high species diversity and ecosystem function. First, I discuss how grazing may be a driver for higher species diversity~~. The second par~~and secondly I~~t~~ discusses the role of species diversity on productivity. Finally, I discuss the socioecological dynamics in the region that have resulted in fewer families investing in pastoralism.

Impact of grazing on species diversity and ANPP~~:~~

The results demonstrate the importance of the traditional grazing system in maintaining high plant diversity and productivity. Significantly higher diversity indices and productivity in grazed areas suggest plant community composition, structure and function benefit or depend indirectly

Formatted: Heading 2 Char, Font: 16 pt, Not Bold, Font color: Text 1

Formatted: Font: (Default) Times New Roman, 12 pt

Formatted: Font: (Default) Times New Roman, 12 pt

Formatted: Font: (Default) Times New Roman, 18 pt, Bold, Font color: Auto

Formatted: Heading 1

Formatted: Font: (Default) Times New Roman, 18 pt, Bold

Formatted: Heading 2 Char, Font: 16 pt, Not Bold, Font color: Text 1

Formatted: Font color: Text 1

on grazing for their persistence in the high-~~altitude~~ elevation alpine pastures of the Himalaya. Grazing may be a driver for higher species diversity in three ways.

First, in free grazing systems like in the study site, higher diversity could be facilitated by the important role livestock play as seed dispersers. Seed dispersal by grazing animals is important in grasslands throughout the world, being vital for conservation of not only species but genetic diversity [64–66]. Grazing animals are dispersal vectors through epizoochory (transporting outside the body e.g. on fur) or endozoochory (transporting after ingestion). Studies have found higher survival of small seeds in the digestive tract of mammals increasing effectiveness of endozoochory [67–69]. This could explain higher PD of small seeded ~~less palatable~~ species such as Orchidaceae and Asteraceae (especially *Anaphalis lactea* and *Lentopodium leontopodioides)* in grazed plots than ungrazed plots~~.~~

Second, grazing results in the reduction in abundance of dominant species by selectively removing palatable species [70]. This explains higher PD for graminoid species (that are palatable) in ungrazed areas. The results show that graminoid species represent 78.19% of the total PD in ungrazed ~~quadrates~~quadrats as compared to 44.34% in grazed ~~quadrates~~quadrats. Graminoid species of Cyperaceae and Poaceae families were more palatable than Juncaceae which was grazed after most palatable species were eaten. Additionally, higher PD of less palatable families such as Asteraceace, Primulaceae and Rosaceae (dominated by *Potentilla* spp) was found in grazed plots.

Third, grazing can increase spatial heterogeneity thus increasing habitat diversity. Free ranging livestock may modify soil properties directly by recycling nutrients and concentrating it from a larger area into small patches through their dung and urine deposition [71–74]. Grazing may also affect soil properties indirectly through their forage preference and grazing intensity. This may selectively affect the survival of some plants that affect soil properties through their rhizosphere [75,76].

Grazing mediated dispersal, foraging preferences and spatial heterogeneity would increase niche breadth and overlap and may promote patches of rarer species thus increasing diversity.

A strong positive correlation between productivity and rainfall has been well documented across the globe [72,77–79]. In the study site monthly productivity in grazed plots were closely associated with rainfall. The monsoon season in the region starts in May, peaking in July and gradually ending in September and October. Peak ANPP in lower and middle elevation ~~quadrates~~quadrats was reached in July and gradually tapered off with the receding monsoon winds (Fig. 2K). The monsoons gradually travels northwards, towards the higher elevation explaining why the higher elevation ~~quadrates~~quadrats showed a slight delay in peak ANPP (in August) (Fig. 2K). Interestingly the migration pattern of the local communities is closely associated to temporal trends in productivity with the Lachenpas moving up to the lower and middle ~~elevation~~ zones in June and July and the Dokpas moving down to the lower elevations (study design's higher ~~elevation~~ zones) around mid-July. Higher ANPP in grazed plots suggests grazing mediated species diversity is an important driver of ANPP.

Relationship between species diversity and ANPP~~:~~

Formatted: Heading 2 Char, Font: 16 pt, Not Bold, Font color: Text 1

Formatted: Heading 2, Adjust space between Latin and Asian text, Adjust space between Asian text and numbers

A strong positive correlation between diversity and productivity (~~fig~~ Fig. 2L) suggests higher biodiversity is an important factor in driving ecosystem functions. The results demonstrating that biomass production increased with diversity conforms to numerous studies from other parts of the world [80–83]. There are three explanations proposed based on experimental studies to understand the role of diversity on productivity. Firstly, complementarity effects occur when more diverse communities facilitate greater niche partitioning allowing for a more complete utilization of resources resulting in increased biomass production [80,82,84]. Secondly, facilitative effects of some species promote the establishment and survival of other co-occurring species influencing biomass production [81,85]. Thirdly, sampling or selection effects suggesting that more diverse plots have a higher probability of containing species with high biomass[81,83,86,87]. The three effects are not mutually exclusive, rather a combined influence of complementarity, facilitative and selection effect of biodiversity influence biomass in a community.

Studies on the impact of grazing on ecosystems have shown mixed results. Negative effects of grazing have been attributed to high grazing intensity owing to overgrazing and high stocking rates or to low grazing intensity including the abandonment of grazing [2,21,22,72,88,89]. The general consensus is that a light to medium grazing intensity is most beneficial [2,72,90]. This study demonstrates that the pastures in Lachen valley have benefitted from grazing of various livestock suggesting the stocking rates are within or below “carrying capacity”. The traditional rotational grazing system practiced by the communities allow for pasture regrowth and recovery, facilitates resilience and limits degradation. Similar results have been shown by studies from other regions underlining the benefits of rotational grazing [91–94].

Socio ecological systems~~:~~

Formatted: Heading 1 Char, Font: 13 pt, Not Bold, Font color: Auto

Formatted: Heading 2, Adjust space between Latin and Asian text, Adjust space between Asian text and numbers

Formatted: Font: Bold

Driven by external constraints and the benefits of urbanization, the local communities have diversified toward other sectors, particularly the tourism industry and as porters for the Indian army. The most immediate reason for this change was the complete closure of the international border after the war of 1962 between India and China. This not only ended trade with Tibet, but also led to a loss of access to vast areas of pastures in TAR. In addition to this the establishment of large army encampments and areas for land mines further reduced areas for grazing to an estimated 40% of the original area [95]. Previously, the transboundary migration also ensured the interaction of a larger pool of pastoral people resulting in intermarriages from a larger region as well as more opportunities to cross breed livestock. Today, most Dokpa children are sent to schools in the city and few return to the rigors of a pastoralist’s life. Some Dokpas prefer working as porters and laborers for the Indian army stationed in the region or in the tourism industry as guides, porters, and taxi drivers. Lachenpas too are investing more into the growing tourism industry and less into animal husbandry that show diminishing monetary returns. The loss of grazing pastures and access to trade, reduced opportunities for marriage and cross-breeding livestock, and the exposure to modern education and urbanization have resulted in a reduction in Dokpa population living in the region from 30 households (130 persons) in the 1950’s [95,96] to ~~fewer than a dozen families today~~just sixteen families [96]. Fewer Dokpas and Lachenpas investing in pastoralism would eventually result in lesser livestock numbers.

Conclusion

The paper demonstrates that grazing enhances species diversity and productivity indicating that the pastures in the region have benefited from grazing by livestock. The results are significant in two respects. First, it suggests that a complete ban on grazing, a common practice in protected areas of developing countries [97–99] may not meet its primary objective of conserving biodiversity. Second, the socioeconomic losses because of the grazing ban can be reduced by reintroducing grazing after sufficient research on altered herd sizes and the impacts of specific livestock and grazing intensities. The results suggest that a management strategy that is mutually beneficial for conservation and livelihoods is not just possible but desirable to maintain higher local and regional biodiversity.

The Moreover, crucial role of the traditional grazing system on biodiversity and ecosystem function, coupled with disinvestment in pastoralism by the locals poses an imminent threat to the landscape that is an intricate socio-ecological system with a long history of co-adaptation with ruminant animal. Pastoralism is associated with other spillover effects such as illegal timber and fuelwood collection, medicinal plants (often rare and endemic) collection and the negative effects of overgrazing as enumerated by Bhagwat et al. [41]. At the same time this study shows, the absence of grazing may result in loss of biodiversity and reduced ecosystem function. The benefits of the traditional free ranging pastoralism cannot be ignored, and a holistic approach is needed to conserve the rich biodiversity of the region. As studies from the region demonstrate the grazing ban has resulted in reduced incomes [41], increase in monocultures in lowlands, decreased manure production in a state that exclusively practices organic farming [39], spread of gregarious species and perceived increase in human wildlife conflict [41,43].
I conclude with research and policy recommendations that should be part of an improved grazing management policy in the state.

1. Experimental studies reintroducing grazing in small areas in regions where the grazing ban was enacted need to be conducted. Studies with the aim to understand the role of different livestock species and different grazing intensities on biodiversity structure and function need to be conducted.
2. Climate change is among the biggest threats to Himalayan rangelands, a region of immense significance for ecosystem services provided to atleast one fifth of humanity and among the most among the most biodiverse in the world [34,37]. Further research on the role of grazing on combating shrub encroachment in response to climate change that threatens habitat loss is imperative.
3. The present grazing ban policy needs to be revisited with a more holistic approach
   a. To understand the role of altered herd sizes on diversity and ecosystem function. A complete ban of grazers meets neither conservation nor livelihood goals. A comprehensive approach taking into account social, ecological, and economic strategies would be required to meet sustainable goals.
   b. Explore a payment for ecosystem services schemes for the services provided by pastoralists in maintaining biodiversity and ecosystem function. These schemes should be aimed at pastoralists moving away from practicing traditional pastoralism.

Formatted: Font: (Default) Times New Roman, 18 pt, Bold, Font color: Auto

Formatted: Heading 1, Adjust space between Latin and Asian text, Adjust space between Asian text and numbers

Formatted: Font: (Default) Times New Roman, 18 pt, Bold

Similar schemes have been used in Switzerland where "*general direct payments*" are made for upland farmers to encourage grazing livestock in more difficult conditions [100].

c. Explore the link between the grazing ban policy and increasing incidences of human wildlife conflict as reported by Bhagwat et al. [41]. An efficient compensation scheme for human wildlife conflict that is affecting marginalized communities needs to be developed.

d. Enact reforms to improve livelihoods of ex-herders who are products of the grazing ban policy. While the government has made efforts to reduce this economic impact yet Gupta et al. [39] report frustration among ex-herders in response to "*a lack of reform to improve ex-herders' livelihoods*" post ban.

Acknowledgements

I would like to thank Dr. Kamaljit Bawa for offering vital comments and edits and Dr. Mahesh Sankaran for his advice on the experimental design. Dharmendra Lamsal assisted with parts of the data collection. Field work was supported by funding from Department of Biotechnology Government of India, ATREE Small Grants for research in Northeast India and Sanofi Genzyme and Oracle Fellowship. I would like to thank the Lachenpa and Dokpa communities of Lachen valley and the Dzumsa for their support and enthusiasm. I would also like to thank reviewer 3 for suggesting the DER algorithm and offering vital comments.

Formatted: Font: (Default) Times New Roman, 18 pt, Bold, Font color: Auto

Formatted: Heading 1, Adjust space between Latin and Asian text, Adjust space between Asian text and numbers

Formatted: Font: (Default) Times New Roman, 18 pt, Bold

References

1. Williams RE, Allred BW, DeNio RM, Paulsen Jr HA. Conservation, Development, and Use of the World's Rangelands. J Range Manag. 1968;21: 355–360. Available: https://www.jstor.org/stable/pdf/3896081.pdf

2. Zhang R, Wang Z, Han G, Schellenberg MP, Wu Q, Gu C. Grazing induced changes in plant diversity is a critical factor controlling grassland productivity in the Desert Steppe, Northern China. Agric Ecosyst Environ. 2018;265: 73–83. doi:10.1016/J.AGEE.2018.05.014

3. Havstad KM, Peters DC, Allen-Diaz B, Bartolome J, Bestelmeyer BT, Briske D, et al. The western United States rangelands, a major resource. In: Wedin WF, Fales SL, editors. Grassland Quietness and Strength for a New American Agriculture. Madison, WI: American Society of Agronomy Inc., Crop Science Society of America Inc., and Soil Science Society of America Inc; 2009. pp. 75–93.

4. Neely C, Bunning S, Wilkes A. Review of evidence on drylands pastoral systems and climate change. Rome: Food and Agriculture Organization of the United Nations, 2009; 2009.

5. Costanza R, d'Arge R, de Groot R, Farber S, Grasso M, Hannon B, et al. The value of the world's ecosystem services and natural capital. Nature. 1997;387: 253–260. doi:10.1038/387253a0

Formatted: Font: (Default) Times New Roman, 18 pt, Bold, Font color: Auto

Formatted: Heading 1

Formatted: Font: (Default) Times New Roman, 18 pt, Bold

6. Sayre NF, McAllister RR, Bestelmeyer BT, Moritz M, Turner MD. Earth Stewardship of rangelands: coping with ecological, economic, and political marginality. Front Ecol Environ. 2013;11: 348–354. doi:10.1890/120333

7. Farley KA, Walsh K, Levine AS. Opportunities and obstacles for rangeland conservation in San Diego County, California, USA. Ecol Soc. 2017;22: 38.

8. Sala OE, Yahdjian L, Havstad K, Aguiar MR. Rangeland ecosystem services: nature's supply and humans' demand. In: Briske DD, editor. Rangeland systems: Processes, management and challenges. Springer International Publishing, Cham; 2017. pp. 467–489.

9. Brunson MW, Huntsinger L. Ranching as a Conservation Strategy: Can Old Ranchers Save the New West? Rangel Ecol Manag. 2008;61: 137–147. doi:10.2111/07-063.1

10. Alkemade R, Reid RS, van den Berg M, de Leeuw J, Jeuken M. Assessing the impacts of livestock production on biodiversity in rangeland ecosystems. Proc Natl Acad Sci U S A. 2013;110: 20900–5. doi:10.1073/pnas.1011013108

11. Little PD. Pastoralism, biodiversity, and the shaping of savanna landscapes in East Africa. Africa (Lond). 1996;66: 37–51. doi:10.2307/1161510

12. Miller DJ. Nomads of the Tibetan Plateau Rangelands in Western China. Part One: Pastoral History. Rangelands. 1998;20: 24–29.

13. Niamir-Fuller M, Huber-Sannwald E. Pastoralism and Achievement of the 2030 Agenda for Sustainable Development: A Missing Piece of the Global Puzzle. In: Lucatello S, Huber-Sannwald E, Espejel I, Martínez-Tagüeña N, editors. Stewardship of Future Drylands and Climate Change in the Global South. Springer, Cham; 2020. pp. 41–55. doi:10.1007/978-3-030-22464-6_3

14. Niamir-Fuller M. Managing mobility in African rangelands. Niamir-Fuller M, editor. London, UK: Intermediate Technology Publications; 1999.

15. Goldman M, Nadasdy P, Turner M, editors. Knowing nature: conversations at the intersection of political ecology and science studies. Chicago, IL: University of Chicago Press; 2011.

16. Sandford S, Scoones I. Improving the efficiency of opportunism: new directions for pastoral development. In: Scoones I, editor. Living with uncertainty: new directions in pastoral development in Africa. London, UK: Intermediate Technology Publications; 1994.

17. Banks T. Property Rights Reform in Rangeland China: Dilemmas On the Road to the Household Ranch. World Dev. 2003;31: 2129–2142. doi:10.1016/J.WORLDDEV.2003.06.010

18. Liu X, Ma Z, Huang X, Li L. How does grazing exclusion influence plant productivity and community structure in alpine grasslands of the Qinghai-Tibetan Plateau? Glob Ecol Conserv. 2020;23: e01066. doi:10.1016/J.GECCO.2020.E01066

19. Western D, Groom R, Worden J. The impact of subdivision and sedentarization of pastoral lands on wildlife in an African savanna ecosystem. Biol Conserv. 2009;142:

2538–2546. doi:10.1016/J.BIOCON.2009.05.025

20. Weber KT, Horst S. Desertification and livestock grazing: The roles of sedentarization, mobility and rest. Pastor Res Policy Pract. 2011;1: 19. doi:10.1186/2041-7136-1-19

21. Camacho O, Dobremez L, Capillon A. Shrub encroachment in pastures in the Alps. Rev géographie Alp. 2008; 89–100. doi:10.4000/rga.575

22. Zhang W, Miller PA, Smith B, Wania R, Koenigk T, Döscher R. Tundra shrubification and tree-line advance amplify arctic climate warming: results from an individual-based dynamic vegetation model. Environ Res Lett. 2013;8: 034023. doi:10.1088/1748-9326/8/3/034023

23. Post E, Pedersen C. Opposing plant community responses to warming with and without herbivores. Proc Natl Acad Sci U S A. 2008;105: 12353–8. doi:10.1073/pnas.0802421105

24. Olofsson J, Oksanen L, Callaghan T, Hulme P., Oksanen T, Suominen O. Herbivores inhibit climate-driven shrub expansion on the tundra. Glob Chang Biol. 2009;15: 2681–2693. doi:10.1111/j.1365-2486.2009.01935.x

25. Myers-Smith IH, Forbes BC, Wilmking M, Hallinger M, Lantz T, Blok D, et al. Shrub expansion in tundra ecosystems: dynamics, impacts and research priorities. Environ Res Lett. 2011;6: 045509. doi:10.1088/1748-9326/6/4/045509

26. Verma M, Schulte to Bühne H, Lopes M, Ehrich D, Sokovnina S, Hofhuis SP, et al. Can reindeer husbandry management slow down the shrubification of the Arctic? J Environ Manage. 2020;267: 110636. doi:10.1016/J.JENVMAN.2020.110636

27. Mittermeier RA, Turner WR, Larsen FW, Brooks TM, Gascon C. Global Biodiversity Conservation: The Critical Role of Hotspots. In: Zachos FE, Habel JC, editors. Biodiversity Hotspots: Distribution and Protection of Conservation Priority Areas. Berlin, Heidelberg: Springer Berlin Heidelberg; 2011. pp. 3–22. doi:10.1007/978-3-642-20992-5_1

28. Ning W, Rawat G, Joshi S, Ismail M, Sharma E, editors. High-altitude rangelands and their interfaces in the Hindu Kush Himalayas. Kathmandu: ICIMOD; 2013.

29. Shrestha UB, Gautam S, Bawa KS. Widespread climate change in the Himalayas and associated changes in local ecosystems. PLoS One. 2012;7: e36741. doi:10.1371/journal.pone.0036741

30. Telwala Y, Brook BW, Manish K, Pandit MK. Climate-induced elevational range shifts and increase in plant species richness in a Himalayan biodiversity epicentre. PLoS One. 2013;8: e57103. doi:10.1371/journal.pone.0057103

31. Pandey R, Khadka KK, Ghimire A, Jha P, Pathak U. Elevational distribution of butterflies in the Himalayas: a case study from Langtang National Park, Nepal. J Mt Sci. 2017;14: 1384–1390. doi:10.1007/s11629-017-4360-9

32. Tiwari A, Fan Z-X, Jump AS, Li S-F, Zhou Z-K. Gradual expansion of moisture sensitive Abies spectabilis forest in the Trans-Himalayan zone of central Nepal associated with climate change. Dendrochronologia. 2017;41: 34–43.

doi:10.1016/J.DENDRO.2016.01.006

33. Anderson K, Fawcett D, Cugulliere A, Benford S, Jones D, Leng R. Vegetation expansion in the subnival Hindu Kush Himalaya. Glob Chang Biol. 2020; gcb.14919. doi:10.1111/gcb.14919

34. Xu J, Grumbine RE, Shrestha A, Eriksson M, Yang X, Wang Y, et al. The melting Himalayas: cascading effects of climate change on water, biodiversity, and livelihoods. Conserv Biol. 2009;23: 520–30. doi:10.1111/j.1523-1739.2009.01237.x

35. Singh C., Paniprahy S, Thapliyal A, Kimothi M., Soni P, Parihar J. Monitoring the alpine treeline shift in parts of the Indian Himalayas using remote sensing. Curr Sci. 2012;102: 559–562.

36. Gaire N., Koirala M, Bhuju D., Borgaonkar H. Treeline dynamics with climate change at the central Nepal Himalaya. Clim Past. 2014;10: 1277–1290. Available: http://moeseprints.incois.gov.in/876/1/Treeline dynamics.pdf

37. Telwala Y. Climate change and alpine flora in Sikkim Himalaya. Climate Change in Sikkim - Patterns, Impacts and Initiatives. 2012. pp. 103–124.

38. Tambe S, Rawat GS. Ecology, economics, and equity of the pastoral systems in the Khangchendzonga National Park, Sikkim Himalaya, India. Ambio. 2009;38: 95–100. doi:10.1579/0044-7447-38.2.95

39. Gupta R, Haider LJ, Österblom H. The theory of cross-scale interactions: an illustration from remote villages in Sikkim, India. Environ Dev Sustain. 2020;22: 3777–3804. doi:10.1007/s10668-019-00329-0

40. Feroze SM, Ray LI, Singh KJ, Singh R. Pastoral yak rearing system is changing with change in climate: an exploration of North Sikkim in Eastern Himalaya. Clim Change. 2018;157: 483–498.

41. Bhagwat S, Diwan M, Venkataramani V. Study of ecological, eocio-economic and livelihood dimensions of grazing exclusion in preotected forests of West Sikkim. Chennai; 2012.

42. Bhagwat S, Diwan M, Venkataramani V. Analysis of Grazing Exclusion Policy through a Climate Change Mitigation Lens: Case Studies from Barsey Rhododendron Sanctuary,West Sikkim. In: Arrawatia ML, Tambe S, editors. Climate Change in Sikkim - Patterns, Impacts and Initiatives. Gangtok: Information and Public Relations Department Government of Sikkim; 2012. pp. 351–368. Available: http://www.sikkimforest.gov.in/climate-change-in-sikkim/21-Chapter_Analysis of Grazing Exclusion Policy through the climate change.pdf

43. Tambe S, Bhutia N, Arrawatia M. People's opinion on the Impacts of "Ban on Grazing" in Barsey Rhododendron Sanctuary. Sikkim, India. Gangtok, Sikkim; 2005.

44. Singh P, Chauhan A. Plant Diversity in Sikkim Himalaya. In: Hajra P, Mugda V, editors. Plant Diversity and Hotspots in India. Calcutta: BSI; 1997. pp. 137–162.

45. Ingty T, Bawa KS. Climate change and indigenous people. In: Arrawatia ML, Tambe S,

editors. Climate Change in Sikkim Patterns, Impacts and Initiatives. Information and Public Relations Department,Government of Sikkim, Gangtok; 2012. pp. 275–290.

46. Briggs JM, Knapp AK. Interannual variability in primary production in tallgrass prairie: climate, soil moisture, topographic position, and fire as determinants of aboveground biomass. Am J Bot. 1995;82: 1024–1030.

47. Smith MD, Knapp AK. Dominant species maintain ecosystem function with non-random species loss. Ecol Lett. 2003;6: 509–517.

48. Rodgers WA, Panwar HS. Planning a wildlife protected area network in India. Wildlife Institute of India, Dehradun; 1988. Available: http://agris.fao.org/agris-search/search.do?recordID=XF9093847

49. Government of Sikkim. 19th Livestock Census, 2012. II. Gangtok, Sikkim; 2012. Available: http://www.sikkim-ahvs.gov.in/Download/Report2.pdf

50. Ingty T. High mountain communities and climate change: adaptation, traditional ecological knowledge, and institutions. Clim Change. 2017;145: 41–55. doi:10.1007/s10584-017-2080-3

51. Joshi S, Shrestha L, Bisht N, Wu N, Ismail M, Dorji T, et al. Ethnic and Cultural Diversity amongst Yak Herding Communities in the Asian Highlands. Sustainability. 2020;12: 957. doi:10.3390/su12030957

52. LP DAAC. ASTER Global Digital Elevation Model (GDEM) v2. NASA EOSDIS Land Processes DAAC,. Sioux Falls, South Dakota; 2014.

53. McNaughton SJ, Milchunas DG, Frank DA. How can net Primary Productivity be Measured in Grazing Ecosystems? Ecology. 1996;77: 974–977. doi:10.2307/2265518

54. Leroy B, Petillon J, Gallon R, Canard A, Ysnel F. Improving occurrence- based rarity metrics in conservation studies by including multiple rarity cut- off points. Insect Conserv Divers. 2012;5: 159-168.

55. Guisande C, Heine J, García-Roselló E, González-Dacosta J, Vilas LG, Perez-Schofield BJG. DER: An algorithm for comparing species diversity between assemblages. Ecol Indic. 2017;81: 41–46. doi:https://doi.org/10.1016/j.ecolind.2017.05.049

56. Menhinick E. A Comparison of Some Species- Individuals Diversity Indices Applied to Samples of Field Insects. Ecology. 1964;45: 859–861.

57. McIntosh R. "An index of diversity and the relation of certain concepts to diversity. Ecology. 1967;48: 392–404.

58. Warwick RM, Clarke KR. Taxonomic distinctness and environmental assessment. J Appl Ecol. 1998;35: 532–543.

59. Clarke KR, Warwick RM. A taxonomic distinctness index and its statistical properties. J Appl Ecol. 1998;35: 523–531.

60. R Development Core Team. A Language and Environment for Statistical Computing. R Foundation for Statistical Computing. Vienna, Austria; 2017. Available: http://www.r-

project.org/

61. Tyler CL, Kowalewski M. Utility of Marine Benthic Associations as a Multivariate Proxy of Paleobathymetry: A Direct Test from Recent Coastal Ecosystems of North Carolina. PLoS One. 2014;9: e95711. doi:10.1371/JOURNAL.PONE.0095711

62. Dexter E, Rollwagen-Bollens G, Bollens SM. The trouble with stress: A flexible method for the evaluation of nonmetric multidimensional scaling. Limnol Oceanogr Methods. 2018;16: 434–443. doi:10.1002/lom3.10257

63. Oksanen J, Blanchet FG, Kindt R, Legendre P, Minchin P., O'Hara R., et al. Vegan: Community Ecology Package. R Package Version 2.2-0. 2014.

64. Janzen DH. Dispersal of Small Seeds by Big Herbivores: Foliage is the Fruit. Am Nat. 1984;123: 338–353. Available: http://copa.acguanacaste.ac.cr:8080/bitstream/handle/11606/1223/DISPERSAL OF SMALL SEEDS BY BIG HERBIVORES FOLIAGE IS THE FRUIT.pdf?sequence=1

65. Couvreur M, Christiaen B, Kris V, Martin V. Large herbivores as mobile links between isolated nature reserves through adhesive seed dispersal. Appl Veg Sci. 2004;7: 229–236. Available: https://www.biw.kuleuven.be/lbh/lbnl/ecology/pdf-files/pdf-art/martine/Couvreuretal_Epizoochory_2004_AVS7 1 .229-236.pdf

66. Plue J, Aavik T, Cousins SAO. Grazing networks promote plant functional connectivity among isolated grassland communities. van Kleunen M, editor. Divers Distrib. 2019;25: 102–115. doi:10.1111/ddi.12842

67. Pakeman RJ, Digneffe G, Small JL. Ecological Correlates of Endozoochory by Herbivores. Funct Ecol. 2002;16: 296–304. Available: http://www.jstor.org/stable/826582

68. Cocyns E, Delporte A, Lens L, Hoffman M. Germination success of temperate grassland species after passage through ungulate and rabbit guts. J Ecol. 2005;93: 353–361. doi:10.1111/j.0022-0477.2005.00982.x

69. Wu G-L, Li X-P, Cheng J-M, Wei X-H, Sun L. Grazing Disturbances Mediate Species Composition of Alpine Meadow Based on Seed Size. Isr J Ecol Evol. 2009;55: 369–379. doi:10.1560/IJEE.55.4.369

70. Lu X, Kelsey KC, Yan Y, Sun J, Wang X, Cheng G, et al. Effects of grazing on ecosystem structure and function of alpine grasslands in Qinghai-Tibetan Plateau: a synthesis. Ecosphere. 2017;8: e01656. doi:10.1002/ecs2.1656

71. Carran RA, Ball PR, Theobald PW, Collins MEG. Soil nitrogen balances in urine-affected areas under two moisture regimes in Southland. New Zeal J Exp Agric. 1982;10: 377–381. doi:10.1080/03015521.1982.10427902

72. McNaughton SJ. Ecology of a Grazing Ecosystem: The Serengeti. Ecol Monogr. 1985;55: 259–294. doi:10.2307/1942578

73. Steinauer EM, Collins SL. Effects of Urine Deposition on Small-Scale Patch Structure in Prairie Vegetation. Ecology. 1995;76: 1195–1205. doi:10.2307/1940926

74. Liu C, Song X, Wang L, Wang D, Zhou X, Liu J, et al. Effects of grazing on soil nitrogen spatial heterogeneity depend on herbivore assemblage and pre-grazing plant diversity. Wan S, editor. J Appl Ecol. 2016;53: 242–250. doi:10.1111/1365-2664.12537

75. Hamilton EW, Frank DA. Can plants simulate soil microbes and their own nutrient supply? Evidence from a grazing tolerant grass. Ecology. 2001;82: 2397–2402. doi:10.1890/0012-9658(2001)082[2397:CPSSMA]2.0.CO;2

76. Wilson CH, Strickland MS, Hutchings JA, Bianchi TS, Flory SL. Grazing enhances belowground carbon allocation, microbial biomass, and soil carbon in a subtropical grassland. Glob Chang Biol. 2018;24: 2997–3009. doi:10.1111/gcb.14070

77. Pandey CB, Singh JS. Rainfall and Grazing Effects on Net Primary Productivity in a Tropical Savanna, India. Ecology. 1992;73: 2007–2021. doi:10.2307/1941451

78. Fay PA, Carlisle JD, Knapp · Alan K., Blair JM, Collins SL. Productivity responses to altered rainfall patterns in a C4-dominated grassland. Oecologia. 2003;137: 245–251. Available: https://www.researchgate.net/profile/John_Blair2/publication/10674171_Productivity_responses_to_altered_rainfall_patterns_in_a_C_4_-dominated_grassland/links/0fcfd50855628aff47000000.pdf

79. Castro SM, Sanchez-Azofeifa GA, Sato H. Effect of drought on productivity in a Costa Rican tropical dry forest. Environ Res Lett. 2018;13: 045001. Available: https://iopscience.iop.org/article/10.1088/1748-9326/aaacbc/pdf

80. Hector A. The Effect of Diversity on Productivity: Detecting the Role of Species Complementarity. Oikos. 1998;82: 597. doi:10.2307/3546380

81. Kelemen A, Török P, Valkó O, Deák B, Tóth K, Tóthmérész B. Both facilitation and limiting similarity shape the species coexistence in dry alkali grasslands. Ecol Complex. 2015;21: 34–38. doi:10.1016/J.ECOCOM.2014.11.004

82. Guerrero-Ramírez NR, Craven D, Reich PB, Ewel JJ, Isbell F, Koricheva J, et al. Diversity-dependent temporal divergence of ecosystem functioning in experimental ecosystems. Nat Ecol Evol. 2017;1: 1639–1642. doi:10.1038/s41559-017-0325-1

83. Sonkoly J, Kelemen A, Valkó O, Deák B, Kiss R, Tóth K, et al. Both mass ratio effects and community diversity drive biomass production in a grassland experiment. Sci Rep. 2019;9: 1848. doi:10.1038/s41598-018-37190-6

84. Reich PB, Tilman D, Isbell F, Mueller K, Hobbie SE, Flynn DFB, et al. Impacts of Biodiversity Loss Escalate Through Time as Redundancy Fades. Science (80-). 2012;336: 589 LP – 592. doi:10.1126/science.1217909

85. Stachowicz JJ. Mutualism, Facilitation, and the Structure of Ecological CommunitiesPositive interactions play a critical, but underappreciated, role in ecological communities by reducing physical or biotic stresses in existing habitats and by creating new habitats on which many species depend. Bioscience. 2001;51: 235–246. doi:10.1641/0006-3568(2001)051[0235:mfatso]2.0.co;2

86. Huston MA. Hidden treatments in ecological experiments: re-evaluating the ecosystem

function of biodiversity. Oecologia. 1997;110: 449–460. doi:10.1007/s004420050180

87. Loreau M. Separating Sampling and Other Effects in Biodiversity Experiments. Oikos. 1998;82: 600–602. Available: https://www.jstor.org/stable/pdf/3546381.pdf?casa_token=Tyj4lVMsMtYAAAAA:nQeur7AJMI64xit1hB_n_XPixj3AEELNPF4PmPgtHD1KvXtkMnKTZgAfh-FSOZ4MYlzvwdkrfc8FvwFn323FiQNq8r_WKQtDz7BvpaM_M9MS8pyeWA

88. Haynes MA, Fang Z, Waller DM. Grazing impacts on the diversity and composition of alpine rangelands in Northwest Yunnan. J Plant Ecol. 2013;6: 122–130. doi:10.1093/jpe/rts021

89. Li X, Liu Z, Wang Z, Wu X, Li X, Hu J, et al. Pathways of Leymus chinensis Individual Aboveground Biomass Decline in Natural Semiarid Grassland Induced by Overgrazing: A Study at the Plant Functional Trait Scale. Wang W, editor. PLoS One. 2015;10: e0124443. doi:10.1371/journal.pone.0124443

90. Pulungan MA, Suzuki S, Gavina MKA, Tubay JM, Ito H, Nii M, et al. Grazing enhances species diversity in grassland communities. Sci Rep. 2019;9: 11201. doi:10.1038/s41598-019-47635-1

91. Brundage AL, Petersen WE. A Comparison Between Daily Rotational Grazing and Continuous Grazing. J Dairy Sci. 1952;35: 623–630. doi:10.3168/jds.S0022-0302(52)93747-8

92. Teague WR, Dowhower SL. Patch dynamics under rotational and continuous grazing management in large, heterogeneous paddocks. J Arid Environ. 2003;53: 211–229. doi:10.1006/JARE.2002.1036

93. Vecchio MC, Bolaños VA, Golluscio RA, Rodríguez AM. Rotational grazing and exclosure improves grassland condition of the halophytic steppe in Flooding Pampa (Argentina) compared with continuous grazing. Rangel J. 2019;41: 1–12. Available: https://doi.org/10.1071/RJ18016

94. Jacobo EJ, Rodríguez AM, Bartoloni N, Deregibus VA. Rotational Grazing Effects on Rangeland Vegetation at a Farm Scale. Rangel Ecol Manag. 2006;59: 249–257. doi:10.2111/05-129R1.1

95. Sharma G, Tambe S, Rawat GS, Arrawatia M. Yak Herding and Associated Transboundary Issues in the Sikkim Himalaya, India. Transboundary challenges and opportunities for yak raising in a changing Hindu Kush Himalayan region. Kathmandu, Nepal: ICIMOD; 2016. pp. 93–112.

96. NBSAP. Sikkim State Biodiversity Strategy and Action Plan. Gangtok, Sikkim; 2002.

97. Lewis M. Cattle and Conservation at Bharatpur: A Case Study in Science and Advocacy. Conserv Soc. 2003;1: 1–21. Available: https://www.jstor.org/stable/26396448?seq=1#metadata_info_tab_contents

98. Dong SK, Gao HW, Xu GC, Hou XY, Long RJ, Kang MY, et al. Farmer and professional attitudes to the large-scale ban on livestock grazing of grasslands in China. Environ Conserv. 2007;34: 246–254. Available: http://www.jstor.org/stable/44521238

99. Gurung B, Nelson KC, Smith JLD. Impact of Grazing Restrictions on Livestock Composition and Husbandry Practices in Madi Valley, Chitwan National Park, Nepal. Environ Conserv. 2009;36: 338–347. Available: https://www.jstor.org/stable/44520031?seq=1#metadata_info_tab_contents

100. Swiss Federal Office for Agriculture. Swiss Agricultural Policy: Objectives, tools, prospects. Bern, Switzerland; 2004. Available: https://www.cbd.int/financial/pes/swiss-pesagriculturalpolicy.pdf

1. **Editors comment:**

Check the spelling

Response

Spelling of *Rhododendron barbatum corrected*

2. **Editors comment:**

repetition

Response

Corrected throughout the paper

3. **Editors comment:**

Ecosystem function?

In the paper I use above ground net primary productivity (ANPP) an integrative measure of ecosystem function. This is a well-established practice where ANPP is used as a proxy for ecosystem function.
I had mentioned it in the abstract and have added it to the 2 broad questions in the Introduction section with citations.

4. **Editors comment:**

This part is more appropriate for conclusion rather than Introduction. Place it appropriately
Response

I have made the edit and placed it in the conclusion section

5. **Editors comment:**

Does the notification says so? It is better if you cite the notification stating the relaxation on grazing ban given to Lachen. Can you mention the rationale why Lachen and Lachung were excluded?
Response

I have added citations for two papers discussing grazing being allowed in selected areas of KNP and one citation for grazing in Lachen and Lachung.

I have not seen the exact notification and do not know why Lachen and Lachung have been excluded.

6. **Editors comment:**

Do we have more recent data?

Response

This is the most recent I found. I am assuming the livestock census like the general census is published every decade.

7. **Editors comment:**

But these areas also have effect on species diversity due to hay extraction.

Response

Yes. That is why I explicitly define what I mean by ungrazed areas. These are *"areas with a history of no grazing for ateast 10 years with permanent fences around them. These areas are used by the locals to make hay."*

8. **Editors comment:**

Remove the word elevation and retain lower zone, middle zone and upper zone. Follow it throughout the text

Response

Changes made throughout the manuscript.

9. **Editors comment:**

How big were the blocks? Were they large enough to have sufficient distance between the grazed and ungrazed areas?

I have made changes to the Vegetation sampling subsection to clarify the experimental design. There were six 30mX30m plots. This included 3 in grazed areas (one for each elevation zone) and 3 in ungrazed areas (one for each elevation zone). In each plot twelve 1mX1m quadrats were laid to quantify diversity. Thus, for each elevation zone I had 24 quadrats (12 in grazed and 12 in ungrazed) with a total of 72 quadrats in all elevations and treatments where I collected data for species diversity. This is explained in the edited lines as

"*I used a randomized block design with two treatments (grazing and ungrazed) and three replicates (lower zone =3000-3500m; middle zone =3500-400m; and higher zone= 4000-4500m) making a total of six 30mX 30m plots (2 treatments X 3 replicates*)"

I made further corrections in the subsection- Impacts of grazing on species diversity and above ground net primary productivity (ANPP)

"Diversity: In each 30mX30 m plot 12 quadrats (1mX1 m) were randomly laid. We had 36 quadrates in grazed areas (12X 3elevation zones) and 36 in ungrazed areas. Thus, a total of 72 quadrats were laid (24 in each zone/ replicate). Data on species composition and percentage cover was collected from each quadrat for herb species only (forbs and graminoids)."

10. **Editors comment:**

Within this small area of 30m x 30 m, how it is possible to have grazed and ungrazed areas. It is not convincing because grazing animals are not restricted within 15 m (roughly as per your design) are for grazing

Response

Please see response comment above. The grazed areas were infact much larger than 30mX30m but laid our 12 plots within that plot (30mX30m) only.

11. Editors comment:

You estimated only herbs or woody vegetation were also included in the quadrat? If woody vegetation were included, your quadrat size is too small and if they are not included, how did you avoid them because in pastures also there are few woody species sparsely grown?

Response

Only herbs (forbs and graminoids) species were sampled in each quadrat. I have added this point in the subsection- Impacts of grazing on species diversity and above ground net primary productivity (ANPP). The grazing pastures we sampled (Phemkaru, Yathang and Chopta valley) had a negligible number of woody vegetation and none fell in our quadrats. In the ungrazed plots (fenced areas) there were few woody vegetation and only along the edges which we anyway avoided to avoid for edge effect.

12. Editors comment:

How did you do it in the ungrazed area?

Response

In the ungrazed areas ANPP was collected at peak standing crop once a year. I have edited the last line in the subsection Impacts of grazing on species diversity and above ground net primary productivity (ANPP) to

"In ungrazed areas twelve 1mX1m quadrats were laid and biomass was collected at peak standing crop (annually)."

13. Editors comment:

Was there a facility to do this in Lachen??

Response

No, there was no facility to oven dry in Lachen. The biomass collected was stored with blotting paper and was sent occasionally (about every 1 week to Gangtok where we had an oven to dry it).

14. Editors comment:

Please see the comments of Reviewer 3 and modify the analysis based on the comments

Response

I have conducted the analysis suggested by reviewer 3. I used the DER algorithm comparing the differences between assemblages in in terms of four components of diversity (1) rarity (Leroy's rarity index), (2) heterogeneity of the grassland community(Menhinick index), (3)evenness (McIntosh index)and (4) taxonomic distinctness (Dstar). I appreciate reviewer 3's suggestion and feel the new analysis greatly enhances the paper. I also used the Kruskal-Wallis test to test the difference between grazing treatments and between different elevations zones for diversity indices and ANPP (table1). Table 1. needs no changes since the results (significance categories) are the same. Table 2 has been omitted since the section on ANOVA has been deleted.

15. Editors comment:

Mention the packages used

Response

I have mentioned it at the end of the data analysis subsection. All analyses were performed on R. The NMDS and ANOSIM analysis were performed using the vegan package in R. I corrected my mistake- vegan is a package in R not a function.
I have also mentioned the package EcoIndR for the DER algorithm.

16. Editors comment:

Provide the list of plant species observed during sampling as supplementary material

Response

Added.

17. Editors comment:

Do not start sentence with numbers

Response

Changes made.

18. Editors comment:

In my viewpoint this analysis does not have any meaning. Also see the comments of Reviewer 3.

Response

Please see response to comment 14. In addition to this comparing diversity indices in grazed versus ungrazed plots is a well-established practice as is evidenced from several published

papers for many years and I have compared them using a non-parametric Kruskal-Wallis test as suggested by reviewer 3. The results were the same (significance categories) and table one needs no changes.

19. Editors comment:

This analysis is not clear. Did you compare the composition among among quadrates? If so, what is the relevance to the objective of this study?

Response

Yes, the Analysis of similarity (ANOSIM) was used to check the degree of dissimilarity of species compositions. As a stand alone the results of the analysis are very interesting but in conjunction with the analysis on species richness, other diversity indices and ANPP, it does give a clearer idea of the role of grazing in diversity and ecosystem function. While without the ANOSIM the results show higher diversity and ANPP in grazed areas, the ANOSIM indicates grazing mediated difference in species assemblages/compositions may be driving these differences.

20. Editors comment:

This is an amazing finding. Hope you have highlighted the reason in the discussion section

Response

Please refer to response to comment 28

21. Editors comment:

Fig.

Number below ten are written in words

Response

Changes made throughout the paper

22. Editors comment:

plant diversity??

Response

Change made

23. Editors comment:

Use elevation uniformly throughout the text. Altitude and elevation are represented in different ways

Response

Changes made

24. Editors comment:

This sentence contradicts with your previous sentence

Response

I can see how the words "less palatable" can be confusing in this point. The species are less palatable but not unpalatable. They are still eaten by livestock unlike the species in the ungrazed plots and the PD values of these species are lower in ungrazed plots. The point I am making is small seeded species that studies show have higher survival during endozoochory have higher PD vlues in grazed versus ungrazed plots. I am not comparing it within grazed plots.

I have removed the words "less palatable" to remove any confusion. It can be reinserted if you feel otherwise.

25. **Editors comment:**

This paragraph looks odd here and away from the objectives of the study

Response

The first and second objective both look at the effects of grazing on plant diversity and productivity. This paragraph primarily discusses ANPP. Not acknowledging the role of rainfall in ANPP trends would suggest grazing is the single most important factor and presents an incomplete picture hence this paragraph is important for the discussion.

26. **Editors comment:**

But is is nowhere shown in the result section

Response

Thank you for pointing this out. I have a reference to "Fig 2K" in this section. This paragraph discusses Fig 2K and shows how ANPP is different in different elevation zones and follows the general rainfall patterns. The results section does refer to figure 2K stating that the ANPP is highest in July (in lower, middle, zones and all combined) and August (in higher zone).

While rainfall data is not shown as it was not available for the study site. In the next sentences we do discuss the rainfall pattern for the region.

27. **Editors comment:**

…the Dokpas moving down to the higher elevation zones ..
Response

Sentence edited as below to clarify

"….Dokpas moving down to the lower elevations (study design's higher zones) around mid-July"

28. **Editors comment:**

I did not see any explanation on why the species composition similarity was very low between grazed and ungrazed areas. It is an interesting findings but need through discussion.
Response

I agree that it is a very interesting result. I do not have an in-depth explanation although the three major points in the discussion, subsection- Impact of grazing on species diversity and ANPP touch upon this finding. The second and third point especially puts forth an argument for this. "*grazing results in the reduction in abundance of dominant species by selectively removing palatable species"* and *"grazing can increase spatial heterogeneity thus increasing habitat diversity"*. Higher pressure to reduce dominant species and higher habitat diversity in grazed areas would facilitate species unique to grazed areas. It is also understandable that a plot where no grazing has occurred for over a decade will develop different species compositions.

Showing differences in species composition is not the objective of the study but the result greatly enhances the discussion on grazing impact on diversity and productivity the primary objective of the study.

29. **Editors comment:**

How? *"This not only ended trade with Tibet, but also led to a loss of vast areas of pastures in TAR."*
Response

I have added the word "access" to read better.

"This not only ended trade with Tibet, but also led to a loss of access to vast areas of pastures in TAR"

Here, I refer to the international border being closed after the war thus the local communities lost access to vast pastures across the border in Tibetan Autonomous region that they used before the border was closed.

30. Editors comment:

What could improve the dairy farming and pastoralism in the region? Highlight some points here

Response

I do not think a step towards improving dairy farming should be taken without sufficient knowledge on the impact that may have. In a region with very dynamic social and economic structures in addition to relatively new pressures due to the burgeoning tourism industry, there are numerous unintended consequences a top down approach may have. Will improved dairy farming incentivize the present and future generations of Dokpas from moving away from nomadic pastoralism? Will a financially successful dairy farming design prevent elite capture and deteriorate the social capital in the present cohorts? These are questions that need to be addressed before investing in improved dairy farming techniques.

To protect pastoralism, I have 2 points (that are an urgent need) discussed in the conclusion, reiterated below. In addition to this market access and reinstating pride in a traditional livelihood that is often looked at as a last resort by the present generation of local communities would go a long way.

1. *"payment for ecosystem services schemes for the services provided by pastoralists in maintaining biodiversity and ecosystem function"*. This has been practiced in other parts of the world notably in Switzerland.
2. *"An efficient compensation scheme for human wildlife conflict that is affecting marginalized communities needs to be developed."*

31. Editors comment:
Highlight – in citation

Response

Citation format has been corrected

Reviewer #1: comment

Abstract:
In the abstract you mentioned "This paper explores the impact of the traditional pastoral system on high elevation plant species in Lachen valley, one of the few regions of Sikkim where the grazing ban was not implemented." Clearly mention in the abstract which were the current grazing stands and which were the ungrazed forests or plots or treatments you selected in your study. You have not mentioned this and thus following your previous sentence of the study site in the Lachen Valley your sentence speaking about your result does not match in the subsequent sentences.

Response

I have added 2 lines in the abstract clearing this point. The second line clarifies "*Ungrazed areas are part of pastures that have been fenced off (preventing grazing) for over a decade and used by the locals for hay formation.*"

Reviewer #1: comment

Please give a clear recommendation as to what the policy makers should do. Do they need to revisit their policy….

Response

Conclusion-point 3 explicitly states that the grazing ban policy needs to be revisited. "*The present grazing ban policy needs to be revisited with a more holistic approach*"

Reviewer #1: comment

….develop strategic short-term and long term strategic action plans through government notification and initiate regulated grazing allowing the herders to graze livestock??

Response

As discussed in the conclusion (point 1) "*Experimental studies reintroducing grazing in small areas in regions where the grazing ban was enacted need to be conducted. Studies with the aim to understand the role of different livestock species and different grazing intensities on biodiversity structure and function need to be conducted.*" Further studies built on our research would be needed to gather more data on different grazing intensities and different livestock species before initiating "regulated grazing allowing the herders to graze livestock"

Reviewer #1: comment

Introduction

The last paragraph should go to the discussion section.
Response

Thank you for pointing this out. It has been moved to the conclusion section on the recommendation of the editor.

Reviewer #1: comment

1. The author can develop a GIS based Hyposometric map with the location of the sampled elevations map along the elevation 3000-4500 m with drainage systems, name of the study sites etc. in a DEM.

Response

Thank you very much for the recommendation. Figure 1 has been redone using a Digital Elevation Model (DEM) derived from ASTER data

Reviewer #1: comment

2. How did you differentiate the grazing with the domestic ungulates and the wild ungulates ?

Response

As stated in the sub-section under Vegetation sampling. *"I studied the combined effects of different grazing species"* (wild and domestic).

"*It was difficult to separate the effects of different grazers since there was much overlap in the grazing areas shared by the different livestock and wild species. Cows, bulls, goats, horses and a few yak shared the same pastures in the lower elevation zone (3000-3500 m asl). Sheep, yak, cows, bulls, goats, and horses shared the middle elevation zones (3500-4000 m asl) and yak, sheep and a few horses grazed in the higher elevation zone (4000-4500 m asl). Thus, I studied the combined effects of different grazing species.*"

Reviewer #1: comment
3. When and where was the fencing laid, was that laid in all the study sites ?

Response

The fenced (ungrazed areas) were laid in all 3 elevation zones. Within Lachen valley there are several areas fenced off by the locals (for atleast 10 years before the study was conducted). These areas are used by the locals to make hay. The herbs are left to grow in these areas throughout the year and removed at the end of the growing season

"Local pastoralists and village representatives were consulted to select grazed and ungrazed areas" One fenced area was in Phemkaru (lower zone), Yathang (middle zone) and Chopta (higher zone). The name of places is mentioned in the edited map (figure 1). The details of fencing is described in the MS in the third sentence of the section on Methods-vegetation sampling

"The ungrazed areas were defined as areas with a history of no grazing for ateast 10 years with permanent fences around them. These areas are used by the locals to make hay. The herbs are left to grow in these areas throughout the year and removed at the end of the growing season."

Reviewer #1: comment
4. What was the study period of this research ?

Response

The study was initiated in 2010 but due to the earthquake of 2011 data collection was disrupted. Preliminary data was recollected in 2012 August- October. Finalized data was collected over a three-year period starting from the growing season of 2013 to the end of the growing season in 2015. I have added this to the materials and methods section under the sub-section Vegetation sampling.

Reviewer #1: comment

5. Which were the livestock, domestic as well as wild ungulates, season of grazing, and the season of your study, is not clear.

Response

Domestic livestock is described in detail in the vegetation sampling section (MS quoted below). We did not monitor wild ungulate population. As stated in this section "*I studied the combined effects of different grazing species"* this include the domestic livestock mentioned as well as wild ungulates.

"Cows, bulls, goats, horses and a few yak shared the same pastures in the lower elevation zone (3000-3500 m asl). Sheep, yak, cows, bulls, goats, and horses shared the middle elevation zones (3500-4000 m asl) and yak, sheep and a few horses grazed in the higher elevation zone (4000-4500 m asl). Thus, I studied the combined effects of different grazing species. "

Reviewer #1: comment

6. While the grazed stands were found to be with higher species richness in all the elevation study sites, please give a list of common palatable and unpalatable species found in both the grazed and unglazed sites. This will help policy maker to understand the conservation of important, or the threatened species.

Response

Please refer to reply to comment no. 12

Reviewer #1: comment

7. Please give number of Dokpa households under Lachen Dzumsa (Gurudongmar-Tsholhamu, and Muguthang), as you have said the population is declining. You have mentioned in the last paragraph before the conclusion section that over a dozen Dokpas are there (???).

Response

The number of households has been revised to an older (2002) source with a citation. The number mentioned in the previous version of the manuscript was a result of conversations with Dokpas who mentioned that there were fewer than a dozen families left actively practicing nomadic pastoralism.

Reviewer #1: comment

8. It is important to note the stocking density to identify the impact of grazing, please justify your result by showing the stocking density in the study elevations. Did you come across any location of overstocking ??

Response

While I agree with the reviewer that calculating stocking density would add value to the study we collected data on type of livestock in plots and not number of individual livestock, and wildlife species were also unaccounted for. If we estimated of stocking densities based on government census data (available for the whole valley and not blocks) it would be inaccurate and misleading to policy makers.

Moreover as Laca in *Rangeland Ecol Manage*.(2009) point out productivity will be affected by grazing method, bite rates and the defoliation process in addition to stocking density. My study focuses on the vegetation response to traditional grazing system comparing grazed areas versus ungrazed (traditionally fenced areas)

Reviewer #1: comment

9. Which species were dominant in the three study elevations, as you said the dominant species were reduced due to grazing. Were they all palatable ?

Response

Graminoid species were most dominant with *Kobresia nepalensis* (Cyperaceae) being the most common species in all 3 elevations. Yes, the graminoid species were all palatable.

I have made no changes to the manuscript as this information is present in paragraph 4 of the discussion and paragraph three of the results section.

Reviewer #1: comment

10. Any information on Pray-Predator relationship, impact of grazing on wildlife, livestock depredation etc. will add value to the MS.

Response

I completely agree, unfortunately this was outside the objective and scope of the study and we did not collect information on wildlife.

Reviewer #1: comment

11. There is a notion that the Human-Wildlife conflict has increased after ban on grazing, please justify if any such information could be generated during the course of this study.

Response

I did not come across such information since the study was conducted in areas where grazing was not banned. I have cited papers from parts of Sikkim that have seen a perceived increase in human wildlife conflict after the grazing ban (reference 38[Bhagwat et al.2012], 40 [Tambe et al. 2005], 88 [Gupta et al. 2020])

Reviewer #1: comment

12. What was the species composition in the grazed plots, how did you identify the species as the palatable species are already grazed by animals?

Response

Species were identified as palatable based on literature reviews, consultations with locals and observations. Species were deemed palatable if they met all 3 above criteria.

I sampled herbs (forbs and graminoid species only). (This has been added in the methods section). Based on the 3 criteria above we were able to confirm that graminoid species were indeed palatable. Especially cyperaceae and poaceae. Juncaceae was less palatable but was still grazed when more palatable species were eaten. (This has been added to the Discussion section- Impacts of grazing on species diversity and ANPP- paragraph 3). I did not classify other species as less palatable unless they did not meet all 3 criteria and grazers avoided these species eg. *Anaphalis lactea Lentopodium leontopodioides* and *Morina longifolia*.

Reviewer #1: comment

13. Clearly mention in your result the species composition based on their season of appearances, snowfall period

Response

Data was collected only during the growing season (mid-April to late October) and not during the snowfall period. I have added as supplementary material a table on species composition.

Reviewer #1: comment

14. It would be nice to give high resolution pictures of the stand of both grazed and un-grazed stands.

Reviewer #1: comment
15. What kind anthro0pogenic pressure were there in grazing stands other than grazing ?

Response

Although close to the road the grazing pastures had no other anthropogenic pressure and maintained solely for grazing. Occasional visits by tourists do occur resulting in trampling only but no construction.

Reviewer #1: comment
16. Also show in your result what is the species richness in the three elevations ?

Response

It is present in table 1. I have added it to the results section too.

Reviewer #1: comment
17. How did you measure ANPP in the grazed and ungrzaed plots, as the plant biomass of grazed plots were eaten up by animals ?

Response

As mentioned in the methods section I followed the difference method by McNaughton et al. (1996) (ref.51). This is a standard method that is most preferred to measure the effect of grazing on plants "*since moveable exclosures account for the amount eaten and the amount that regrows between feeding bouts"* (McNaughton et al. 1996). Since this is a highly cited paper there is no need to repeat the details of the method more than what is already detailed in the methods section.

Reviewer #1: comment
18. Which species do you think are dispersed by animals?

Response

This is a very interesting question. Any names here would be only conjecture and a separate study focusing on epizoochory and endozoochory would be needed to confirm. Unfortunately, this was outside the objective and scope of the study. If I would have to guess Asteraceae species that may be principally wind pollinated may be occasionally dispersed long distances by endozoochory.

Reviewer #2: General Comments:

1. Author should give special attention in the spellings of the botanical names of the plant species, grammar and punctuation

Response

Thank you. I have made the edits and corrections

Reviewer #2: comment
2. Repetition of words encountered in places which needs to be checked

Response

Thank you. I have made the edits and corrections

Specific Comments:

Reviewer #2: comment
1. Abstract

• Such long introduction in abstract is not required. Just restrict to one or two sentences.
• Point out the methods applied for the study in one or two sentences

Response

Thank you. Changes made.

Reviewer #2: comment
• Highlight the most significant findings in the result section in one or two sentences instead of generalizing it. For eg., The multidimensional scaling and ANOSIM (Analysis of Similarities) pointed to significant differences in plant species assemblages in grazing and ungrazed areas. Significant at what level?
Response

Thank you. Changes made.

Reviewer #2: comment

2. Introduction

• The author have mentioned in third paragraph (line 6 – 8) that “Open grazing was phased out over the next six years in all reserved forests with exception of Lachen and Lachung valley and alpine pastures in the Kangchenzonga National Park”. Logically, grazing is strictly not allowed

in National Parks. This statement may negatively impact the UNESCO's World Heritage Site status of the KNP. Author should avoid using such statement or should provide supporting references.

Response

I have added citations for two papers discussing grazing being allowed in selected areas of KNP.

Reviewer #2: comment

• The last paragraph in the introduction section looks more like a part of the discussion based on the present study. Hence, move the paragraph to the discussion section.

Response

I have made the edit and placed it in the conclusion section

Reviewer #2: comment

3. Materials and Methods

• Normally, above 2000 m asl elevation are considered as higher elevation. Therefore, it would not be appropriate to label the elevation above 3000 m asl as lower elevation zone, middle elevation zone or high elevation zone. Try to find some suitable terminology or replace it with lower zone , middle zone and upper zone duly mentioning the elevation range in the text as well as in the map.

Response

Thank you for the suggestion, I have made the changes

Reviewer #2: comment

• Method is vague. How is it possible to have both grazed and un-grazed area in the same plot of 30 x 30 m? Needs to be clarified. Further, the unit must be accurate. Either write 30 m x 30 m or replace it with 30 x 30 sq. m.

Response

I have made changes to the Materials and methods section under sub-section vegetation sampling

Reviewer #2: comment

• Author have mentioned the establishment of 12 movable enclosure cages to study the biomass growth. It is not clear weather all the cages were set up in the same plot at a time. If not then, it is important to mention as how many cages were set up in each plot.

Response

I have made changes to the Materials and methods section under sub-section vegetation sampling

Reviewer #2: comment

• Major rewriting of the methods under the sub-heading "Impact of grazing…..", a part of the vegetation sampling section is needed to clear the confusion.

Response

Thank you. Changes made

Reviewer #2: comment

• Under the subheading 1, 2 para, line 4: The standing biomass of was……..what does it mean? please specify or correct the statement.

Response

I have changed it to "*aboveground standing biomass (vegetation)*"

Reviewer #2: comment

• Data analysis: Table1 & 2 is the part of the result section; hence, remove the mention of table 1 & 2 from the data analysis section.

Response

Changes made

Reviewer #2: comment

• What is the logic behind calculating Shannon Index and Simpson diversity index? As both the indexes calculates the diversity taking into account the species richness and the relative abundance of the species. It is better if the author calculates the dominance index.

Response

As suggested by reviewer #3 I compared the differences between assemblages in terms of rarity (Leroy's rarity index), heterogeneity of the grassland community(Menhinick index), evenness (McIntosh index)and taxonomic distinctness (Dstar). The four the components of diversity were combined into a single measurement using the DER_algorithm in the EcoINDR package in R. In addition to this I also calculated proportional dominance.

If permitted, I would prefer to leave both Simpson diversity and Shannon indices if not I would prefer to leave in the Shannon index. Having both indices may be useful as part of a larger meta-analysis. Recent publications (eg. Lu et al. 2017;Ecosphere) have used both indices as part of their meta-analysis. I think meta-analyses play an important role as a policy tools and my primary data would help feed into larger analyses.

Reviewer #2: comment

4. Results

Species diversity indices:

• Why the same result is depicted in table as well as the box plot (Fig 2 A-D, Table 1). Either use table or figure.

Response

I am fine with fig 2A-D being removed. Although I would prefer it being retained since the boxplots give an idea of the spread of data that the standard deviations (in the table) fail to display.

Reviewer #2: comment

• Para 1, line no.: 8 & 9. Author have mentioned that "Grazing had a significant (ANOVA $p<0.001$) effect all diversity indices in each elevation zone and all elevation zones combined but from table 2, it is clear that the effect of grazing is not significant for diversity indices in higher elevation. Address this mistake.

Response

Thank you for catching this. Changes have been made to table 2.

Reviewer #2: comment

• Para 1, last 3 lines. Rewrite the sentence "Unlike grazing, elevation did not have a significant effect (ANOVA $p<0.05$)diversity indices (fig. 2G-I, table 2) except S (fig. 2F, table 2)" as "Unlike grazing, elevation did not have a significant effect on diversity indices (Fig 2G-I) except S ($p<0.05$) [Fig. 2F, table 2)".

Response

Changes made

Reviewer #2: comment

• Para 2. Line no. 2-3. The sentence is unclear whether the author is trying to compare the species composition between grazed and un-grazed treatment in different zones or amongst the replicates of grazing treatment in different zones. Needs rewriting.

Response

Thank you. I have rewritten the 2 lines

Reviewer #2: comment

• ANPP: Rewrite the sentence "The mean ANPP for all grazed quadrates (all elevation zones combined), lower and middle elevation zone grazed quadrates was highest in July (Fig 2K) and in the higher elevation zone grazed quadrates in August (Fig 2K)" as "The mean ANPP was highest in July for all grazed quadrates in lower zone, middle zone and all zones combined but it was highest in August in higher zone (Fig 2 K)".

Response

Changes made

Reviewer #2: comment

5. Discussion

• It should focus more on discussing the result of the present study duly supported by the relevant studies in other Himalayan region. Needs major rewriting especially the first two parts.

Reviewer #3: comment

The manuscript has been written well and the research design appears to be rigorous. However, the description I believe is a little confusing. The way I understood the design is:
Elevation: Low, Middle, High
Blocks: G (grazed) and U (ungrazed)
Replicates: 3/elevation [3x3 = 9]
Plots: 30x30m, one per block [total = 9] This is confusing – was the 30x30m plot equally straddling G and U areas?
Sampling quadrats: 24 per block (12G and 12U) [24, 1x1m quadrats/elevation, and a total of 216 for the study – while implied (perhaps), this should be mentioned in the text]
If this is correct, a simple chronological statement or a schematic of the levels and arrangement will make following the experimental set-up easy for readers.

Response

I have made changes to the Materials and methods section under sub-section vegetation sampling that should clarify the study design

Reviewer #3: comment

One of my major concerns with the manuscript is the analyses carried out with the diversity values. Here the author has used a variety of diversity measures – Shannon entropy, Simpson concentration, and Pielous's evenness. Nothing is alarming about these indices by themselves, but the problem arises once these values are further analyzed, say using an ANOVA. First off, these indices are limited in range – usually between 0 and 1, for Shannon's entropy when normalized, for example; exhibiting a floor-ceiling effect and violating the assumptions of a traditional ANOVA. This often leads to wrong results and comparisons. Details of this problem may be further understood by reviewing the paper by Lou Jost in Oikos 2006 – Diversity and Entropy. There are methods suggested in this paper that makes comparing entropies more meaningful. Therefore, instead of using an ANOVA to tease apart the difference among treatments, I suggest that the author use a more comprehensive and inclusive algorithm such as the DER – for comparing species diversity. See Guisande et al. 2017. This can be done in R (package EcoIndR). These are more contemporary techniques and overcome the flaws of other traditional comparison techniques.

Please see attached an annotated content extracted pdf (lit rev not included) of the manuscript for minor edits that will help the paper read well.

Other than that, the research represented by the manuscript is timely and important in understanding ways of restoring the plant diversity of the alpine regions of the Himalaya and elsewhere, especially using adaptive management policies.

Response

Thank you very much for the suggestion. I have done the analysis using the DER algorithm comparing the differences between assemblages in in terms of rarity (Leroy's rarity index), heterogeneity of the grassland community(Menhinick index), evenness (McIntosh index)and taxonomic distinctness (Dstar). I also used the Kruskal-Wallis test in place of the t-test used earlier. I also used the Kruskal-Wallis test to test the difference between grazing treatment and between different elevations zones for diversity indices and ANPP and removed the ANOVA analysis.
