# Supplementary material for: Pastoralism in the highest peaks: Role of the traditional grazing systems in maintaining biodiversity and ecosystem function in the alpine Himalaya
Source: PLoS One. 2021 Jan 7;16(1):e0245221. doi: 10.1371/journal.pone.0245221 (PMC7790420; doi:10.1371/journal.pone.0245221)
Supplement: Attachment — Submitted filename: Comments.docx [file pone.0245221.s003.docx]

Title: Pastoralism in the highest peaks: Role of the traditional grazing systems in maintaining biodiversity and ecosystem function in the alpine Himalaya.

This study is very interesting to scientifically validate to show the impact of grazing exclusion policy of the Department of Forest and Environment, Government of Sikkim. Grazing in the forests starting from the Sub-tropical, temperate, alpine forests and the Trans Himalayan rangelands was and is a traditional system of animal farming in the Sikkim Himalaya. During 1998-2005 the Department of Forest and Environment, Government of Sikkim imposed “Ban on Grazing” in Protected Areas and Reserved Forests in the sub-tropical, temperate and alpine areas of East, South, West and North Sikkim with the objective of conserving the biodiversity and improvement of degraded forest. Grazing was considered as detrimental to forest, and biodiversity, illegal activities and extraction. While the scientific validation of the impact of grazing on forest and biodiversity deterioration was not available with the policy implementing agencies.

Therefore, although carried out only in a specific location in North Sikkim, this study has provided scientific knowledge and understanding that grazing exclusion will result into loss of species richness and decline ecosystem functioning. Thus, this study can be a basis to the policy implementing agencies for revisiting the “Grazing Ban Policy” and develop a new policy framework. Equally, this study will also provide opportunities for the researcher, scientists and planners to take forward the similar study in other parts of Sikkim in particular or the Himalaya at large.

**Specific comments**

Abstract:

In the abstract you mentioned “This paper explores the impact of the traditional pastoral system on high elevation plant species in Lachen valley, one of the few regions of Sikkim where the grazing ban was not implemented.”

Clearly mention in the abstract which were the current grazing stands and which were the ungrazed forests or plots or treatments you selected in your study. You have not mentioned this and thus following your previous sentence of the study site in the Lachen Valley your sentence speaking about your result does not match in the subsequent sentences.

Please give a clear recommendation as to what the policy makers should do. Do they need to revisit their policy, develop strategic short-term and long term strategic action plans through government notification and initiate regulated grazing allowing the herders to graze livestock??

**Introduction**

The last paragraph should go to the discussion section.

1. The author can develop a GIS based Hyposometric map with the location of the sampled elevations map along the elevation 3000-4500 m with drainage systems, name of the study sites etc. in a DEM.
2. How did you differentiate the grazing with the domestic ungulates and the wild ungulates ?
3. When and where was the fencing laid, was that laid in all the study sites ?
4. What was the study period of this research ?
5. Which were the livestock, domestic as well as wild ungulates, season of grazing, and the season of your study, is not clear.
6. While the grazed stands were found to be with higher species richness in all the elevation study sites, please give a list of common palatable and unpalatable species found in both the grazed and unglazed sites. This will help policy maker to understand the conservation of important, or the threatened species.
7. Please give number of Dokpa households under Lachen Dzumsa (Gurudongmar-Tsholhamu, and Muguthang), as you have said the population is declining. You have mentioned in the last paragraph before the conclusion section that over a dozen Dokpas are there (???).
8. It is important to note the stocking density to identify the impact of grazing, please justify your result by showing the stocking density in the study elevations. Did you come across any location of overstocking ??
9. Which species were dominant in the three study elevations, as you said the dominant species were reduced due to grazing. Were they all palatable ?
10. Any information on Pray-Predator relationship, impact of grazing on wildlife, livestock depredation etc. will add value to the MS.
11. There is a notion that the Human-Wildlife conflict has increased after ban on grazing, please justify if any such information could be generated during the course of this study.
12. What was the species composition in the grazed plots, how did you identify the species as the palatable species are already grazed by animals ?
13. Clearly mention in your result the species composition based on their season of appearances, snowfall period
14. It would be nice to give high resolution pictures of the stand of both grazed and un-grazed stands.
15. What kind anthro0pogenic pressure were there in grazing stands other than grazing ?
16. Also show in your result what is the species richness in the three elevations ?
17. How did you measure ANPP in the grazed and ungrzaed plots, as the plant biomass of grazed plots were eaten up by animals ?
18. Which species do you think are dispersed by animals ?
