# Supplementary material for: Pastoralism in the highest peaks: Role of the traditional grazing systems in maintaining biodiversity and ecosystem function in the alpine Himalaya
Source: PLoS One. 2021 Jan 7;16(1):e0245221. doi: 10.1371/journal.pone.0245221 (PMC7790420; doi:10.1371/journal.pone.0245221)
Supplement: Attachment — Submitted filename: Response to Reviewers.docx [file pone.0245221.s006.docx]

1. **Editors comment:**

Check the spelling

***Response***

Spelling of *Rhododendron barbatum corrected*

2. **Editors comment:**

repetition

***Response***

Corrected throughout the paper

3. **Editors comment:**

Ecosystem function?

In the paper I use above ground net primary productivity (ANPP) an integrative measure of ecosystem function. This is a well-established practice where ANPP is used as a proxy for ecosystem function.

I had mentioned it in the abstract and have added it to the 2 broad questions in the Introduction section with citations.

4. **Editors comment:**

This part is more appropriate for conclusion rather than Introduction. Place it appropriately

***Response***

I have made the edit and placed it in the conclusion section

5. **Editors comment:**

Does the notification says so? It is better if you cite the notification stating the relaxation on grazing ban given to Lachen. Can you mention the rationale why Lachen and Lachung were excluded?

***Response***

I have added citations for two papers discussing grazing being allowed in selected areas of KNP and one citation for grazing in Lachen and Lachung.

I have not seen the exact notification and do not know why Lachen and Lachung have been excluded.

6. **Editors comment:**

Do we have more recent data?

***Response***

This is the most recent I found. I am assuming the livestock census like the general census is published every decade.

7. **Editors comment:**

But these areas also have effect on species diversity due to hay extraction.

***Response***

Yes. That is why I explicitly define what I mean by ungrazed areas. These are *“areas with a history of no grazing for ateast 10 years with permanent fences around them. These areas are used by the locals to make hay.”*

8. **Editors comment:**

Remove the word elevation and retain lower zone, middle zone and upper zone. Follow it throughout the text

***Response***

Changes made throughout the manuscript.

9. **Editors comment:**

How big were the blocks? Were they large enough to have sufficient distance between the grazed and ungrazed areas?

I have made changes to the Vegetation sampling subsection to clarify the experimental design. There were six 30mX30m plots. This included 3 in grazed areas (one for each elevation zone) and 3 in ungrazed areas (one for each elevation zone). In each plot twelve 1mX1m quadrats were laid to quantify diversity. Thus, for each elevation zone I had 24 quadrats (12 in grazed and 12 in ungrazed) with a total of 72 quadrats in all elevations and treatments where I collected data for species diversity. This is explained in the edited lines as

“*I used a randomized block design with two treatments (grazing and ungrazed) and three replicates (lower zone =3000-3500m; middle zone =3500-400m; and higher zone= 4000-4500m) making a total of six 30mX 30m plots (2 treatments X 3 replicates*)”

I made further corrections in the subsection- Impacts of grazing on species diversity and above ground net primary productivity (ANPP)

*“**Diversity:* *In each 30mX30 m plot 12 quadrats (1mX1 m) were randomly laid. We had 36 quadrates in grazed areas (12X 3elevation zones) and 36 in ungrazed areas. Thus, a total of 72 quadrats were laid (24 in each zone/ replicate). Data on species composition and percentage cover was collected from each quadrat for herb species only (forbs and graminoids).*”

10. **Editors comment:**

Within this small area of 30m x 30 m, how it is possible to have grazed and ungrazed areas. It is not convincing because grazing animals are not restricted within 15 m (roughly as per your design) are for grazing

***Response***

Please see response comment above. The grazed areas were infact much larger than 30mX30m but laid our 12 plots within that plot (30mX30m) only.

11. **Editors comment:**

You estimated only herbs or woody vegetation were also included in the quadrat? If woody vegetation were included, your quadrat size is too small and if they are not included, how did you avoid them because in pastures also there are few woody species sparsely grown?

***Response***

Only herbs (forbs and graminoids) species were sampled in each quadrat. I have added this point in the subsection- Impacts of grazing on species diversity and above ground net primary productivity (ANPP). The grazing pastures we sampled (Phemkaru, Yathang and Chopta valley) had a negligible number of woody vegetation and none fell in our quadrats. In the ungrazed plots (fenced areas) there were few woody vegetation and only along the edges which we anyway avoided to avoid for edge effect.

12. **Editors comment:**

How did you do it in the ungrazed area?

***Response***

In the ungrazed areas ANPP was collected at peak standing crop once a year. I have edited the last line in the subsection Impacts of grazing on species diversity and above ground net primary productivity (ANPP) to

*“In ungrazed areas twelve 1mX1m quadrats were laid and biomass was collected at peak standing crop (annually).”*

13. **Editors comment:**

Was there a facility to do this in Lachen??

***Response***

No, there was no facility to oven dry in Lachen. The biomass collected was stored with blotting paper and was sent occasionally (about every 1 week to Gangtok where we had an oven to dry it).

14. **Editors comment:**

Please see the comments of Reviewer 3 and modify the analysis based on the comments

***Response***

I have conducted the analysis suggested by reviewer 3. I used the DER algorithm comparing the differences between assemblages in in terms of four components of diversity (1) rarity (Leroy’s rarity index), (2) heterogeneity of the grassland community( Menhinick index), (3)evenness (McIntosh index)and (4) taxonomic distinctness (Dstar). I appreciate reviewer 3’s suggestion and feel the new analysis greatly enhances the paper. I also used the Kruskal-Wallis test to test the difference between grazing treatments and between different elevations zones for diversity indices and ANPP (table1). Table 1. needs no changes since the results (significance categories) are the same. Table 2 has been omitted since the section on ANOVA has been deleted.

15. **Editors comment:**

Mention the packages used

***Response***

I have mentioned it at the end of the data analysis subsection. All analyses were performed on R. The NMDS and ANOSIM analysis were performed using the vegan package in R. I corrected my mistake- vegan is a package in R not a function.

I have also mentioned the package EcoIndR for the DER algorithm.

16. **Editors comment:**

Provide the list of plant species observed during sampling as supplementary material

***Response***

Added.

17. **Editors comment:**

Do not start sentence with numbers

***Response***

Changes made.

18. **Editors comment:**

In my viewpoint this analysis does not have any meaning. Also see the comments of Reviewer 3.

***Response***

Please see response to comment 14. In addition to this comparing diversity indices in grazed versus ungrazed plots is a well-established practice as is evidenced from several published papers for many years and I have compared them using a non-parametric Kruskal-Wallis test as suggested by reviewer 3. The results were the same (significance categories) and table one needs no changes.

19. **Editors comment:**

This analysis is not clear. Did you compare the composition among among quadrates? If so, what is the relevance to the objective of this study?

***Response***

Yes, the Analysis of similarity (ANOSIM) was used to check the degree of dissimilarity of species compositions. As a stand alone the results of the analysis are very interesting but in conjunction with the analysis on species richness, other diversity indices and ANPP, it does give a clearer idea of the role of grazing in diversity and ecosystem function. While without the ANOSIM the results show higher diversity and ANPP in grazed areas, the ANOSIM indicates grazing mediated difference in species assemblages/compositions may be driving these differences.

20. **Editors comment:**

This is an amazing finding. Hope you have highlighted the reason in the discussion section

***Response***

Please refer to response to comment 28

21. **Editors comment:**

Fig.

Number below ten are written in words

***Response***

Changes made throughout the paper

22. **Editors comment:**

plant diversity??

***Response***

Change made

23. **Editors comment:**

Use elevation uniformly throughout the text. Altitude and elevation are represented in different ways

***Response***

Changes made

24. **Editors comment:**

This sentence contradicts with your previous sentence

***Response***

I can see how the words “less palatable” can be confusing in this point. The species are less palatable but not unpalatable. They are still eaten by livestock unlike the species in the ungrazed plots and the PD values of these species are lower in ungrazed plots. The point I am making is small seeded species that studies show have higher survival during endozoochory have higher PD vlues in grazed versus ungrazed plots. I am not comparing it within grazed plots.

I have removed the words “less palatable” to remove any confusion. It can be reinserted if you feel otherwise.

25. **Editors comment:**

This paragraph looks odd here and away from the objectives of the study

***Response***

The first and second objective both look at the effects of grazing on plant diversity and productivity. This paragraph primarily discusses ANPP. Not acknowledging the role of rainfall in ANPP trends would suggest grazing is the single most important factor and presents an incomplete picture hence this paragraph is important for the discussion.

26. **Editors comment:**

But is is nowhere shown in the result section

***Response***

Thank you for pointing this out. I have a reference to “Fig 2K” in this section. This paragraph discusses Fig 2K and shows how ANPP is different in different elevation zones and follows the general rainfall patterns. The results section does refer to figure 2K stating that the ANPP is highest in July (in lower, middle, zones and all combined) and August (in higher zone).

While rainfall data is not shown as it was not available for the study site. In the next sentences we do discuss the rainfall pattern for the region.

27. **Editors comment:**

…the Dokpas moving down to the higher elevation zones ..

***Response***

Sentence edited as below to clarify

*“….Dokpas moving down to the lower elevations (study design’s higher zones) around mid-July”*

28. **Editors comment:**

I did not see any explanation on why the species composition similarity was very low between grazed and ungrazed areas. It is an interesting findings but need through discussion.

***Response***

I agree that it is a very interesting result. I do not have an in-depth explanation although the three major points in the discussion, subsection- Impact of grazing on species diversity and ANPP touch upon this finding. The second and third point especially puts forth an argument for this. “*grazing results in the reduction in abundance of dominant species by selectively removing palatable species”* and *“grazing can increase spatial heterogeneity thus increasing habitat diversity”.* Higher pressure to reduce dominant species and higher habitat diversity in grazed areas would facilitate species unique to grazed areas. It is also understandable that a plot where no grazing has occurred for over a decade will develop different species compositions.

Showing differences in species composition is not the objective of the study but the result greatly enhances the discussion on grazing impact on diversity and productivity the primary objective of the study.

29. **Editors comment:**

How? *“This not only ended trade with Tibet, but also led to a loss of vast areas of pastures in TAR.”*

***Response***

I have added the word “access” to read better.

*“This not only ended trade with Tibet, but also led to a loss of access to vast areas of pastures in TAR”*

Here, I refer to the international border being closed after the war thus the local communities lost access to vast pastures across the border in Tibetan Autonomous region that they used before the border was closed.

30. **Editors comment:**

What could improve the dairy farming and pastoralism in the region? Highlight some points here

***Response***

I do not think a step towards improving dairy farming should be taken without sufficient knowledge on the impact that may have. In a region with very dynamic social and economic structures in addition to relatively new pressures due to the burgeoning tourism industry, there are numerous unintended consequences a top down approach may have. Will improved dairy farming incentivize the present and future generations of Dokpas from moving away from nomadic pastoralism? Will a financially successful dairy farming design prevent elite capture and deteriorate the social capital in the present cohorts? These are questions that need to be addressed before investing in improved dairy farming techniques.

To protect pastoralism, I have 2 points (that are an urgent need) discussed in the conclusion, reiterated below. In addition to this market access and reinstating pride in a traditional livelihood that is often looked at as a last resort by the present generation of local communities would go a long way.

1. *“payment for ecosystem services schemes for the services provided by pastoralists in maintaining biodiversity and ecosystem function”*. This has been practiced in other parts of the world notably in Switzerland.
2. *“An efficient compensation scheme for human wildlife conflict that is affecting marginalized communities needs to be developed.”*
31. **Editors comment:**

Highlight – in citation

***Response***

Citation format has been corrected

**Reviewer #1: comment**

Abstract:
In the abstract you mentioned “This paper explores the impact of the traditional pastoral system on high elevation plant species in Lachen valley, one of the few regions of Sikkim where the grazing ban was not implemented.” Clearly mention in the abstract which were the current grazing stands and which were the ungrazed forests or plots or treatments you selected in your study. You have not mentioned this and thus following your previous sentence of the study site in the Lachen Valley your sentence speaking about your result does not match in the subsequent sentences.

***Response***

I have added 2 lines in the abstract clearing this point. The second line clarifies “*Ungrazed areas are part of pastures that have been fenced off (preventing grazing) for over a decade and used by the locals for hay formation.*”

**Reviewer #1: comment**

Please give a clear recommendation as to what the policy makers should do. Do they need to revisit their policy….

***Response***

Conclusion-point 3 explicitly states that the grazing ban policy needs to be revisited. “*The present grazing ban policy needs to be revisited with a more holistic approach*”

**Reviewer #1: comment**

….develop strategic short-term and long term strategic action plans through government notification and initiate regulated grazing allowing the herders to graze livestock??

***Response***

As discussed in the conclusion (point 1) “*Experimental studies reintroducing grazing in small areas in regions where the grazing ban was enacted need to be conducted. Studies with the aim to understand the role of different livestock species and different grazing intensities on biodiversity structure and function need to be conducted.*” Further studies built on our research would be needed to gather more data on different grazing intensities and different livestock species before initiating “regulated grazing allowing the herders to graze livestock”

**Reviewer #1: comment**

Introduction

The last paragraph should go to the discussion section.
***Response***

Thank you for pointing this out. It has been moved to the conclusion section on the recommendation of the editor.

**Reviewer #1: comment**

1. The author can develop a GIS based Hyposometric map with the location of the sampled elevations map along the elevation 3000-4500 m with drainage systems, name of the study sites etc. in a DEM.

***Response***

Thank you very much for the recommendation. Figure 1 has been redone using a Digital Elevation Model (DEM) derived from ASTER data

**Reviewer #1: comment**

2. How did you differentiate the grazing with the domestic ungulates and the wild ungulates ?

***Response***

As stated in the sub-section under Vegetation sampling. *“I studied the combined effects of different grazing species”* (wild and domestic).

“*It was difficult to separate the effects of different grazers since there was much overlap in the grazing areas shared by the different livestock and wild species. Cows, bulls, goats, horses and a few yak shared the same pastures in the lower elevation zone (3000-3500 m asl). Sheep, yak, cows, bulls, goats, and horses shared the middle elevation zones (3500-4000 m asl) and yak, sheep and a few horses grazed in the higher elevation zone (4000-4500 m asl). Thus, I studied the combined effects of different grazing species.*”

**Reviewer #1: comment**
3. When and where was the fencing laid, was that laid in all the study sites ?

***Response***

The fenced (ungrazed areas) were laid in all 3 elevation zones. Within Lachen valley there are several areas fenced off by the locals (for atleast 10 years before the study was conducted). These areas are used by the locals to make hay. The herbs are left to grow in these areas throughout the year and removed at the end of the growing season

*“Local pastoralists and village representatives were consulted to select grazed and ungrazed areas”* One fenced area was in Phemkaru (lower zone), Yathang (middle zone) and Chopta (higher zone). The name of places is mentioned in the edited map (figure 1). The details of fencing is described in the MS in the third sentence of the section on Methods-vegetation sampling

“*The ungrazed areas were defined as areas with a history of no grazing for ateast 10 years with permanent fences around them. These areas are used by the locals to make hay. The herbs are left to grow in these areas throughout the year and removed at the end of the growing season.”*

**Reviewer #1: comment**
4. What was the study period of this research ?

***Response***

The study was initiated in 2010 but due to the earthquake of 2011 data collection was disrupted. Preliminary data was recollected in 2012 August- October. Finalized data was collected over a three-year period starting from the growing season of 2013 to the end of the growing season in 2015. I have added this to the materials and methods section under the sub-section Vegetation sampling.

**Reviewer #1: comment**

5. Which were the livestock, domestic as well as wild ungulates, season of grazing, and the season of your study, is not clear.

***Response***

Domestic livestock is described in detail in the vegetation sampling section (MS quoted below). We did not monitor wild ungulate population. As stated in this section “*I studied the combined effects of different grazing species”* this include the domestic livestock mentioned as well as wild ungulates.

*“Cows, bulls, goats, horses and a few yak shared the same pastures in the lower elevation zone (3000-3500 m asl). Sheep, yak, cows, bulls, goats, and horses shared the middle elevation zones (3500-4000 m asl) and yak, sheep and a few horses grazed in the higher elevation zone (4000-4500 m asl). Thus, I studied the combined effects of different grazing species. “*

**Reviewer #1: comment**

6. While the grazed stands were found to be with higher species richness in all the elevation study sites, please give a list of common palatable and unpalatable species found in both the grazed and unglazed sites. This will help policy maker to understand the conservation of important, or the threatened species.

***Response***

Please refer to reply to comment no. 12

**Reviewer #1: comment**

7. Please give number of Dokpa households under Lachen Dzumsa (Gurudongmar-Tsholhamu, and Muguthang), as you have said the population is declining. You have mentioned in the last paragraph before the conclusion section that over a dozen Dokpas are there (???).

***Response***

The number of households has been revised to an older (2002) source with a citation. The number mentioned in the previous version of the manuscript was a result of conversations with Dokpas who mentioned that there were fewer than a dozen families left actively practicing nomadic pastoralism.

**Reviewer #1: comment**

8. It is important to note the stocking density to identify the impact of grazing, please justify your result by showing the stocking density in the study elevations. Did you come across any location of overstocking ??

***Response***

While I agree with the reviewer that calculating stocking density would add value to the study we collected data on type of livestock in plots and not number of individual livestock, and wildlife species were also unaccounted for. If we estimated of stocking densities based on government census data (available for the whole valley and not blocks) it would be inaccurate and misleading to policy makers.

Moreover as Laca in *Rangeland Ecol Manage*.(2009) point out productivity will be affected by grazing method, bite rates and the defoliation process in addition to stocking density. My study focuses on the vegetation response to traditional grazing system comparing grazed areas versus ungrazed (traditionally fenced areas)

**Reviewer #1: comment**

9. Which species were dominant in the three study elevations, as you said the dominant species were reduced due to grazing. Were they all palatable ?

***Response***

Graminoid species were most dominant with *Kobresia nepalensis* (Cyperaceae) being the most common species in all 3 elevations. Yes, the graminoid species were all palatable.

I have made no changes to the manuscript as this information is present in paragraph 4 of the discussion and paragraph three of the results section.

**Reviewer #1: comment**

10. Any information on Pray-Predator relationship, impact of grazing on wildlife, livestock depredation etc. will add value to the MS.

***Response***

I completely agree, unfortunately this was outside the objective and scope of the study and we did not collect information on wildlife.

**Reviewer #1: comment**

11. There is a notion that the Human-Wildlife conflict has increased after ban on grazing, please justify if any such information could be generated during the course of this study.

***Response***

I did not come across such information since the study was conducted in areas where grazing was not banned. I have cited papers from parts of Sikkim that have seen a perceived increase in human wildlife conflict after the grazing ban (reference 38[Bhagwat et al.2012], 40 [Tambe et al. 2005], 88 [Gupta et al. 2020])

**Reviewer #1: comment**

12. What was the species composition in the grazed plots, how did you identify the species as the palatable species are already grazed by animals?

***Response***

Species were identified as palatable based on literature reviews, consultations with locals and observations. Species were deemed palatable if they met all 3 above criteria.

I sampled herbs (forbs and graminoid species only). (This has been added in the methods section). Based on the 3 criteria above we were able to confirm that graminoid species were indeed palatable. Especially cyperaceae and poaceae. Juncaceae was less palatable but was still grazed when more palatable species were eaten. (This has been added to the Discussion section- Impacts of grazing on species diversity and ANPP- paragraph 3). I did not classify other species as less palatable unless they did not meet all 3 criteria and grazers avoided these species eg. *Anaphalis lactea* *Lentopodium leontopodioides* and *Morina longifolia.*

**Reviewer #1: comment**

13. Clearly mention in your result the species composition based on their season of appearances, snowfall period

***Response***

Data was collected only during the growing season (mid-April to late October) and not during the snowfall period. I have added as supplementary material a table on species composition.

**Reviewer #1: comment**
14. It would be nice to give high resolution pictures of the stand of both grazed and un-grazed stands.

**Reviewer #1: comment**
15. What kind anthro0pogenic pressure were there in grazing stands other than grazing ?

***Response***

Although close to the road the grazing pastures had no other anthropogenic pressure and maintained solely for grazing. Occasional visits by tourists do occur resulting in trampling only but no construction.

**Reviewer #1: comment**
16. Also show in your result what is the species richness in the three elevations ?

***Response***

It is present in table 1. I have added it to the results section too.

**Reviewer #1: comment**
17. How did you measure ANPP in the grazed and ungrzaed plots, as the plant biomass of grazed plots were eaten up by animals ?

***Response***

As mentioned in the methods section I followed the difference method by McNaughton et al. (1996) (ref.51). This is a standard method that is most preferred to measure the effect of grazing on plants “*since moveable exclosures account for the amount eaten and the amount that regrows between feeding bouts”* (McNaughton et al. 1996). Since this is a highly cited paper there is no need to repeat the details of the method more than what is already detailed in the methods section.

**Reviewer #1: comment**
18. Which species do you think are dispersed by animals?

***Response***

This is a very interesting question. Any names here would be only conjecture and a separate study focusing on epizoochory and endozoochory would be needed to confirm. Unfortunately, this was outside the objective and scope of the study. If I would have to guess Asteraceae species that may be principally wind pollinated may be occasionally dispersed long distances by endozoochory.

**Reviewer #2:** General Comments:

1. Author should give special attention in the spellings of the botanical names of the plant species, grammar and punctuation

***Response***

Thank you. I have made the edits and corrections

**Reviewer #2: comment**
2. Repetition of words encountered in places which needs to be checked

***Response***

Thank you. I have made the edits and corrections

Specific Comments:

**Reviewer #2: comment**
1. Abstract

• Such long introduction in abstract is not required. Just restrict to one or two sentences.
• Point out the methods applied for the study in one or two sentences

***Response***

Thank you. Changes made.

**Reviewer #2: comment**
• Highlight the most significant findings in the result section in one or two sentences instead of generalizing it. For eg., The multidimensional scaling and ANOSIM (Analysis of Similarities) pointed to significant differences in plant species assemblages in grazing and ungrazed areas. Significant at what level?
***Response***

Thank you. Changes made.

**Reviewer #2: comment**

2. Introduction

• The author have mentioned in third paragraph (line 6 – 8) that “Open grazing was phased out over the next six years in all reserved forests with exception of Lachen and Lachung valley and alpine pastures in the Kangchenzonga National Park”. Logically, grazing is strictly not allowed in National Parks. This statement may negatively impact the UNESCO’s World Heritage Site status of the KNP. Author should avoid using such statement or should provide supporting references.

***Response***

I have added citations for two papers discussing grazing being allowed in selected areas of KNP.

**Reviewer #2: comment**

• The last paragraph in the introduction section looks more like a part of the discussion based on the present study. Hence, move the paragraph to the discussion section.

***Response***

I have made the edit and placed it in the conclusion section

**Reviewer #2: comment**

3. Materials and Methods

• Normally, above 2000 m asl elevation are considered as higher elevation. Therefore, it would not be appropriate to label the elevation above 3000 m asl as lower elevation zone, middle elevation zone or high elevation zone. Try to find some suitable terminology or replace it with lower zone , middle zone and upper zone duly mentioning the elevation range in the text as well as in the map.
***Response***

Thank you for the suggestion, I have made the changes

**Reviewer #2: comment**

• Method is vague. How is it possible to have both grazed and un-grazed area in the same plot of 30 x 30 m? Needs to be clarified. Further, the unit must be accurate. Either write 30 m x 30 m or replace it with 30 x 30 sq. m.

***Response***

I have made changes to the Materials and methods section under sub-section vegetation sampling

**Reviewer #2: comment**

• Author have mentioned the establishment of 12 movable enclosure cages to study the biomass growth. It is not clear weather all the cages were set up in the same plot at a time. If not then, it is important to mention as how many cages were set up in each plot.

***Response***

I have made changes to the Materials and methods section under sub-section vegetation sampling

**Reviewer #2: comment**
• Major rewriting of the methods under the sub-heading “Impact of grazing…..”, a part of the vegetation sampling section is needed to clear the confusion.
***Response***

Thank you. Changes made

**Reviewer #2: comment**

• Under the subheading 1, 2 para, line 4: The standing biomass of was……..what does it mean? please specify or correct the statement.
***Response***

I have changed it to “*aboveground standing biomass (vegetation)*”

**Reviewer #2: comment**

• Data analysis: Table1 & 2 is the part of the result section; hence, remove the mention of table 1 & 2 from the data analysis section.

***Response***

Changes made

**Reviewer #2: comment**

• What is the logic behind calculating Shannon Index and Simpson diversity index? As both the indexes calculates the diversity taking into account the species richness and the relative abundance of the species. It is better if the author calculates the dominance index.

***Response***

As suggested by reviewer #3 I compared the differences between assemblages in terms of rarity (Leroy’s rarity index), heterogeneity of the grassland community( Menhinick index), evenness (McIntosh index)and taxonomic distinctness (Dstar). The four the components of diversity were combined into a single measurement using the DER_algorithm in the EcoINDR package in R. In addition to this I also calculated proportional dominance.

If permitted, I would prefer to leave both Simpson diversity and Shannon indices if not I would prefer to leave in the Shannon index. Having both indices may be useful as part of a larger meta-analysis. Recent publications (eg. Lu et al. 2017;Ecosphere) have used both indices as part of their meta-analysis. I think meta-analyses play an important role as a policy tools and my primary data would help feed into larger analyses.

**Reviewer #2: comment**

4. Results

Species diversity indices:

• Why the same result is depicted in table as well as the box plot (Fig 2 A-D, Table 1). Either use table or figure.

***Response***

I am fine with fig 2A-D being removed. Although I would prefer it being retained since the boxplots give an idea of the spread of data that the standard deviations (in the table) fail to display.

**Reviewer #2: comment**

• Para 1, line no.: 8 & 9. Author have mentioned that “Grazing had a significant (ANOVA p<0.001) effect all diversity indices in each elevation zone and all elevation zones combined but from table 2, it is clear that the effect of grazing is not significant for diversity indices in higher elevation. Address this mistake.

***Response***

Thank you for catching this. Changes have been made to table 2.

**Reviewer #2: comment**

• Para 1, last 3 lines. Rewrite the sentence “Unlike grazing, elevation did not have a significant effect (ANOVA p<0.05)diversity indices (fig. 2G-I, table 2) except S (fig. 2F, table 2)” as “Unlike grazing, elevation did not have a significant effect on diversity indices (Fig 2G-I) except S (p<0.05) [Fig. 2F, table 2)”.

***Response***

Changes made

**Reviewer #2: comment**

• Para 2. Line no. 2-3. The sentence is unclear whether the author is trying to compare the species composition between grazed and un-grazed treatment in different zones or amongst the replicates of grazing treatment in different zones. Needs rewriting.

***Response***

Thank you. I have rewritten the 2 lines

**Reviewer #2: comment**

• ANPP: Rewrite the sentence “The mean ANPP for all grazed quadrates (all elevation zones combined), lower and middle elevation zone grazed quadrates was highest in July (Fig 2K) and in the higher elevation zone grazed quadrates in August (Fig 2K)” as “The mean ANPP was highest in July for all grazed quadrates in lower zone, middle zone and all zones combined but it was highest in August in higher zone (Fig 2 K)”.
***Response***

Changes made

**Reviewer #2: comment**

5. Discussion

• It should focus more on discussing the result of the present study duly supported by the relevant studies in other Himalayan region. Needs major rewriting especially the first two parts.

**Reviewer #3: comment**

The manuscript has been written well and the research design appears to be rigorous. However, the description I believe is a little confusing. The way I understood the design is:
Elevation: Low, Middle, High
Blocks: G (grazed) and U (ungrazed)
Replicates: 3/elevation [3x3 = 9]
Plots: 30x30m, one per block [total = 9] This is confusing – was the 30x30m plot equally straddling G and U areas?
Sampling quadrats: 24 per block (12G and 12U) [24, 1x1m quadrats/elevation, and a total of 216 for the study – while implied (perhaps), this should be mentioned in the text]
If this is correct, a simple chronological statement or a schematic of the levels and arrangement will make following the experimental set-up easy for readers.

***Response***

I have made changes to the Materials and methods section under sub-section vegetation sampling that should clarify the study design

**Reviewer #3: comment**

One of my major concerns with the manuscript is the analyses carried out with the diversity values. Here the author has used a variety of diversity measures – Shannon entropy, Simpson concentration, and Pielous’s evenness. Nothing is alarming about these indices by themselves, but the problem arises once these values are further analyzed, say using an ANOVA. First off, these indices are limited in range – usually between 0 and 1, for Shannon’s entropy when normalized, for example; exhibiting a floor-ceiling effect and violating the assumptions of a traditional ANOVA. This often leads to wrong results and comparisons. Details of this problem may be further understood by reviewing the paper by Lou Jost in Oikos 2006 – Diversity and Entropy. There are methods suggested in this paper that makes comparing entropies more meaningful. Therefore, instead of using an ANOVA to tease apart the difference among treatments, I suggest that the author use a more comprehensive and inclusive algorithm such as the DER – for comparing species diversity. See Guisande et al. 2017. This can be done in R (package EcoIndR). These are more contemporary techniques and overcome the flaws of other traditional comparison techniques.

Please see attached an annotated content extracted pdf (lit rev not included) of the manuscript for minor edits that will help the paper read well.

Other than that, the research represented by the manuscript is timely and important in understanding ways of restoring the plant diversity of the alpine regions of the Himalaya and elsewhere, especially using adaptive management policies.

***Response***

Thank you very much for the suggestion. I have done the analysis using the DER algorithm comparing the differences between assemblages in in terms of rarity (Leroy’s rarity index), heterogeneity of the grassland community( Menhinick index), evenness (McIntosh index)and taxonomic distinctness (Dstar). I also used the Kruskal-Wallis test in place of the t-test used earlier. I also used the Kruskal-Wallis test to test the difference between grazing treatment and between different elevations zones for diversity indices and ANPP and removed the ANOVA analysis.
