# Supplementary material for: Pastoralism in the highest peaks: Role of the traditional grazing systems in maintaining biodiversity and ecosystem function in the alpine Himalaya
Source: PLoS One. 2021 Jan 7;16(1):e0245221. doi: 10.1371/journal.pone.0245221 (PMC7790420; doi:10.1371/journal.pone.0245221)
Supplement: Attachment — Submitted filename: Response to Reviewers.docx [file pone.0245221.s008.docx]

1. Please address this comment

Discussion: It should focus more on discussing the result of the present study duly supported by the relevant studies in other Himalayan region. Needs major rewriting especially the first two parts

I have added a paragraph in the discussion section discussing my results and studies from other parts of the world and the Himalaya.

2. This looks like a phone number rather than a permit number

Thank you for catching this. I have corrected the permit details.

3. Avoid this value in abstract

Value removed

4. Should they be in alphabetical order?

I have added the keywords in order of role/ importance.

5. Delete one dot

Deleted

6. Modify 'after the declaration of grazing ban'

Modified

7. Remove from here and take it to acknowledgments section. The permit number given by you looks like a phone number of the forest department. Provide the permit number

I have fixed the permit number. The journal requires that the permit number is written in the Methods section so I have added it to the Methods paragraph and tried to maintain the flow as below

*“The study was conducted in Lachen valley in the North district of the state of Sikkim in India. Necessary field research permits were obtained from the Department of Forest, Environment & Wildlife Management, Government of Sikkim, India – Letter No. 16/SRO/F dated 20/04/2010 and Ref. No. 101 /PCCF/F dated 03/02/2012.”*

8. Two

Has been fixed throughout the MS

9. You have mentioned in your response sheet that you have quoted the reference paper but I do not see them here. To make your argument strong enough, please quote the reference(s).

Citation added

10. Now??

Accepted change

11. Colour code in the legend and map do not match. Please check and rectify

Thank you for catching this. The error has been rectified

12. You are a single author in the article

We changed to I throughout the manuscript

13. excluded would be a better term

Change accepted

14. Check

Deleted extra word

15. Check the framing of the sentence

*“The stress function assesses how well the two-dimensional plot represent actual sample relationships calculated”* changed to “*The stress function assesses how well the calculated sample relationships are represented in the two-dimensional plot”.*

16. check sentence framing

*“The analysis showed all diversity indices (S,H,D and J) were significantly higher (p<0.001) in grazed quadrats than ungrazed quadrats…”* changed to “*The analysis showed that the diversity indices( S,H,D and J) were significantly higher (p<0.001) in grazed quadrats than ungrazed quadrats..”*

17. small letter 'l', middle, h(igher), T(able)

Changes accepted.

18. Add “,” delete “.”

Changes accepted throughout

19. Check whether there is any overlap between Figure 2 and Table 1. I suggest to keep only one, which is most representative to the data

I would prefer to keep both table 1 and figure 2 for the following reasons

a. Figure 2K & L are not represented in table 1. Hence, figure 2K & L is required.
b. Figure 2A to J show the spread of the data while table 1 with standard deviation values shows the variance from means at quadrats level. The boxplots are important to indicate the symmetry (skewness) of the data and median while table 1 shows the mean and standard deviation but cannot indicate the skewness of the data that is informative.
c. Table 1 also quantifies the differences that is important to underline the difference between treatments that if removed will have to be reproduced in the results section.

I have added the units (g/m^2^) to the ANPP column in table 1.

Based on the above reasons I would prefer to have both table 1 and figure 2 retained in the paper.

20. keep it in bracket

Changes accepted throughout the paper

21. check the sentence framing

*“In grazed plots Rumex nepalensis and Primula dendiculata were the only forbs representing the five most dominant species the remaining three were represented by graminoid species Kobresia nepalensis, Festuca coelestis (Poaceae) andJuncus sp (Juncaceae)”* changed to

“*Rumex nepalensis and Primula dendiculata were the only forbs representing the five most dominant species in grazed plots. The other three species were represented by graminoid species namely Kobresia nepalensis, Festuca coelestis (Poaceae) andJuncus sp (Juncaceae)”*

22. Check

The analysis showed that elevation had a significant (p<0.001) role on ANPP. )

Extra bracket removed

23. check the sentence framing

*“quadratsquadrats Productivity was significantly higher in grazed areas than in ungrazed areas (table1).” Corrected to*

*“Productivity was significantly higher in grazed areas than in ungrazed areas (table1)”*

24. Please remove this part

Removed

25. Repetition

Removed.

26. Do you want to keep this?

Removed

27. Provide the complete reference

Fixed

28. This is an incomplete reference

Fixed

29. Oikos is an established journal. Just give doi, all these details are not needed.

Link removed

30. Updated version is available

I cited a number from this version that is not available in the updated version.

31. Provide doi rather than this link

Link removed

32. doi

Fixed

33. Colour code in the legend and the map does not match. Correct it

Corrected. Thank you.
